# Inhibitors of thiol-mediated uptake†

**DOI:** 10.1039/d0sc05447j

**Published:** 2020-11-18

**Authors:** Yangyang Cheng, Anh-Tuan Pham, Takehiro Kato, Bumhee Lim, Dimitri Moreau, Javier López-Andarias, Lili Zong, Naomi Sakai, Stefan Matile

**Affiliations:** Department of Organic Chemistry, University of Geneva Geneva Switzerland stefan.matile@unige.ch http://www.unige.ch/sciences/chiorg/matile/ +41 22 379 6523

## Abstract

Ellman's reagent has caused substantial confusion and concern as a probe for thiol-mediated uptake because it is the only established inhibitor available but works neither efficiently nor reliably. Here we use fluorescent cyclic oligochalcogenides that enter cells by thiol-mediated uptake to systematically screen for more potent inhibitors, including epidithiodiketopiperazines, benzopolysulfanes, disulfide-bridged γ-turned peptides, heteroaromatic sulfones and cyclic thiosulfonates, thiosulfinates and disulfides. With nanomolar activity, the best inhibitors identified are more than 5000 times better than Ellman's reagent. Different activities found with different reporters reveal thiol-mediated uptake as a complex multitarget process. Preliminary results on the inhibition of the cellular uptake of pseudo-lentivectors expressing SARS-CoV-2 spike protein do not exclude potential of efficient inhibitors of thiol-mediated uptake for the development of new antivirals.

## Abstract 2

Thiol-reactive inhibitors for the cellular entry of cyclic oligochalcogenide (COC) transporters and SARS-CoV-2 spike pseudo-lentivirus are reported.

## Graphical abstract

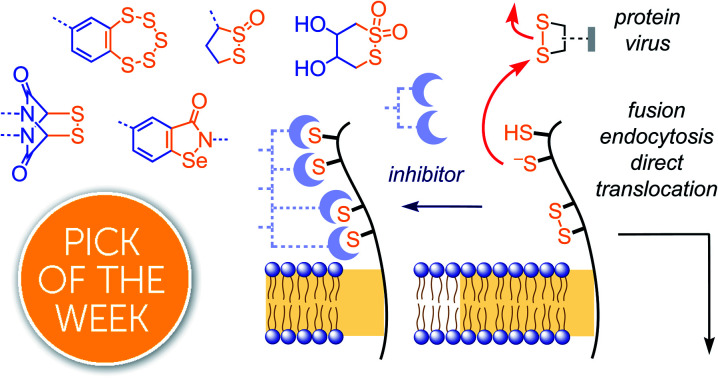

Thiol-mediated uptake^1–10^ has been developed to explain surprisingly efficient cellular uptake of substrates attached to thiol-reactive groups, most notably disulfides. The key step of this mechanism is the dynamic covalent thiol-disulfide exchange between disulfides of the substrates and exofacial thiols on cell surfaces (Fig. 1). The covalently bound substrate then enters the cell either by fusion, endocytosis, or direct translocation across the plasma membrane into the cytosol. Thiol-disulfide exchange has been confirmed to play an essential role in the cellular entry of some viruses^1,11–14^ and toxins.^2^ Indeed, diphtheria toxin and HIV were among the first to be recognized to enter cells *via* thiol-mediated uptake.^1,2^ The involvement of cell-surface thiols in cellular uptake is most often probed by inhibition with Ellman's reagent (DTNB). However, this test is not always reliable, in part due to the comparably poor reactivity of DTNB, and the comparably high reactivity of the disulfide obtained as a product. Thus, the importance of thiol-mediated uptake for viral entry and beyond remains, at least in part, unclear.

**Fig. 1:**
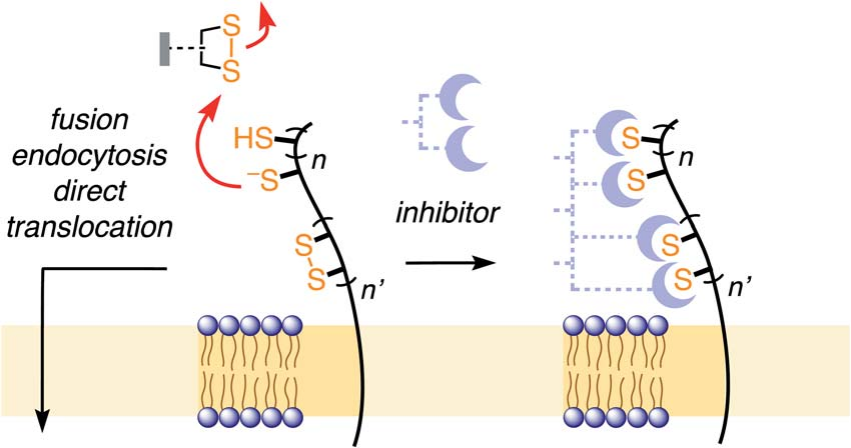
In thiol-mediated uptake, dynamic covalent exchange with thiols on the cell surface precedes entry through different mechanisms. Inhibition of thiol-mediated uptake by removal of exofacial thiols and disulfides could thus afford new antivirals.

We became interested in thiol-mediated uptake^3–5^ while studying the cytosolic delivery of substrates such as drugs, probes and also larger objects like proteins or quantum dots with cell-penetrating poly(disulfide)s.^6^ Our recent focus shifted to cyclic oligochalcogenides (COCs) to increase speed and selectivity of dynamic covalent thiol-oligochalcogenide exchange, and, most importantly, to assure reversibility, *i.e.*, mobility during uptake, with a covalently tethered, intramolecular leaving group.^7^ With increasingly unorthodox COC chemistry, from strained disulfides^7,8^ and diselenides^9^ to adaptive dynamic covalent networks produced by polysulfanes,^10^ uptake activities steadily increased. Their high activities suggested that the same, or complementary, COCs could also function as powerful inhibitors of thiol-mediated uptake that ultimately might perhaps lead to antivirals. In the following, this hypothesis is developed further.

Fluorescently labeled COCs 1^8^ and 2^10^ were selected as reporters for the screening of thiol-mediated uptake inhibitors because of their high activity, their destination in the cytosol, and their different characteristics (Fig. 2). The COC in 1 is an epidithiodiketopiperazine (ETP). With a CSSC dihedral angle ∼0°, ETPs drive ring tension to the extreme.^15,16^ Ring-opening thiol-disulfide exchange is ultrafast, and the released thiols are acidic enough to continue exchanging in neutral water, including ring closure.^8^ This unique exchange chemistry coincides with efficient cellular uptake and poor retention on thiol affinity columns.^8^

**Fig. 2:**
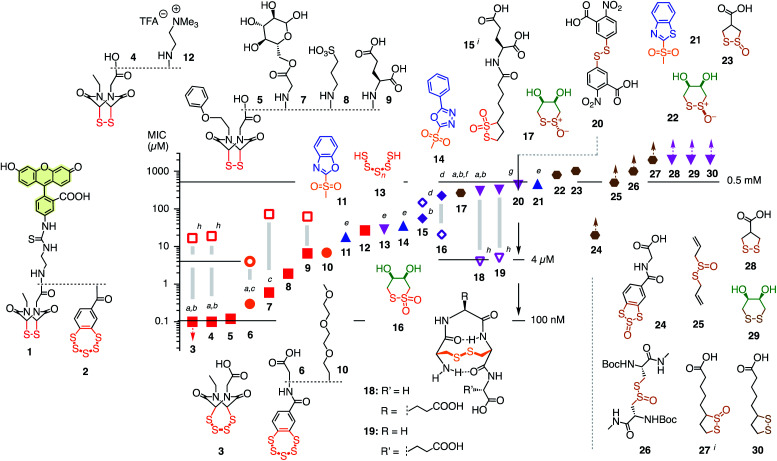
Structure of reporters 1 and 2 and inhibitor candidates 3–30 with their concentrations needed to inhibit by ∼15% (MIC) the uptake of 1 (1 h pre-incubation with inhibitors, 30 min incubation with reporter, filled symbols) and 2 (4 h pre-incubation, empty symbols). Red squares: ETPs; orange circles: BPSs; blue upward triangles: heteroaromatic sulfones; purple diamonds: thiosulfonates; magenta downward triangles: di- and polysulfides; brown hexagons: thiosulfinates. Symbols with upward arrows: MIC not reached at the highest concentration tested. Symbols with downward arrows indicate the lowest concentration tested already exceeds the MIC. (a) Similarly active upon co-incubation of reporters and inhibitor; (b–d) similarly (b), less (c), or more (d) active upon co-incubation in the presence of serum (mostly 6 h); (e) pre-incubation for 15 min; (f) isomerizes into *cis*22; (g) V-shaped DRC (see Fig. 3f); (h) pre-incubation for 30 min, co-incubation with 2; (i) mixture of regioisomers.

The COC in 2 is a benzopolysulfane (BPS). Like ETPs, BPSs occur in natural products and have inspired total synthesis.^17^ Unlike ETPs, BPSs are not strained but evolve into adaptive networks of extreme sulfur species for cells to select from. Uptake efficiencies and retention on thiol affinity columns exceed other COCs clearly.^10,18^

With COCs 1 and 2 as cell-penetrating reporters, a fully automated, fluorescent microscopy image-based high-content high-throughput (HCHT)^19^ inhibitor screening assay was developed. HeLa cells in multiwell plates are incubated with a reporter at constant and inhibitors at varying concentrations and incubation times. Hindered reporter uptake then causes decrease of fluorescence inside of cells (Fig. 3a). Automated data analysis^19^ was established to extract average fluorescence intensity per cell and, at the same time, cell viability from propidium iodide negative nuclei count (Fig. 3 and S3–S6†). Standard assay conditions consisted of pre-incubation of HeLa cells with inhibitors for different periods of time, followed by the removal of inhibitors and the addition of reporters, thus excluding possible interactions between the two in the extracellular environment. In alternative co-incubation conditions, inhibitors were not removed before the addition of reporters to allow for eventual interactions between the two.

**Fig. 3:**
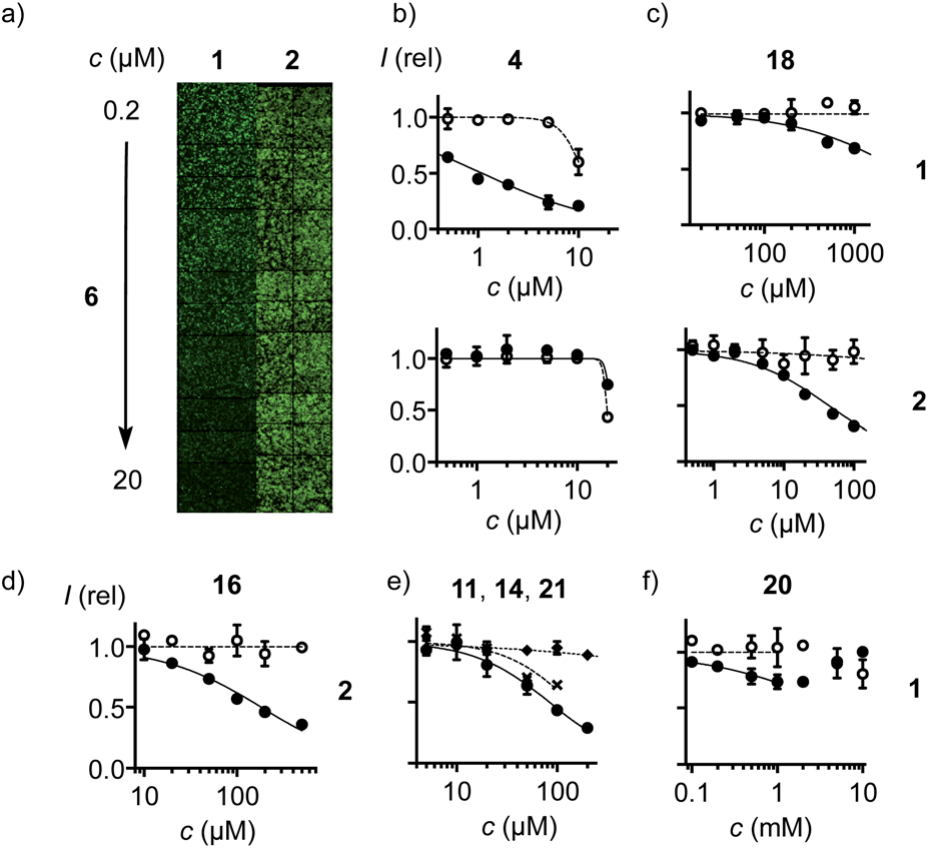
(a) Fluorescence image of HCHT plates (4 images per well) with HeLa cells pre-incubated with 6 (30 min) followed by co-incubation with 1 (left) and 2 (right, 10 μM each) for constant 30 min. (b–f) HCHT data showing relative fluorescence intensity (filled symbols) and cell viability (empty symbols) of HeLa cells after (b) pre-incubation with 4 for 1 h, followed by washing and incubation with 1 (top), or pre-incubation with 4 for 30 min, followed by co-incubation with 4 and 2 (bottom). (c) As in (b) with 18. (d) As in (b) after incubation for 4 h with 16 followed by incubation with 2. (e) As in (b) after pre-incubation with 11 (circles), 14 (crosses), or 21 (diamonds) for 15 min, followed by washing and incubation with 1. (f) As in (b) after pre-incubation with 20 (30 min), followed by washing and incubation with 1.

Among the very high number of thiol-reactive probes, compounds 3–30 were selected based on promise, experience, availability and accessibility. Main focus was on COCs offering increasingly extreme sulfur chemistry because dynamic covalent thiol-oligochalcogenide exchange with different intramolecular leaving groups promises access to different exchange cascades for the intramolecular and, perhaps, also intermolecular crosslinking of the target proteins. More hydrophilic, often anionic COCs were preferred to prevent diffusion into cells and thus minimize toxicity. The expectation was that from such a sketchy outline of an immense chemical space, leads could be identified for future, more systematic exploration. Reporters 1 and 2 and candidates 3–30 were prepared by substantial multistep synthesis (Schemes S1–S11 and Fig. S47–S93,† commercially available: 20, 25, 30). Inhibitors were numbered in the order of efficiency against reporter 1, evaluated by their minimum inhibitory concentrations (MICs), *i.e.*, concentrations that cause a ∼15% reduction of reporter uptake in cells (Fig. 2 and Tables S1–S37†). We chose to use MICs because half-maximal inhibitions could not always be reached due to the onset of toxicity, formally anticooperative, or even V-shaped dose–response curves (DRCs, *e.g.*, Fig. 3b–f, all DRCs can be found in the ESI, Fig. S7–S43†). MICs are usually below the half-maximal cell growth inhibition concentration (GI_50_, Tables S1–S37†).

Among the most potent inhibitors of ETP reporter 1 were ETPs 4 and 5 (Fig. 2, 3b). This intriguing self-inhibition was even surpassed by the expanded cyclic tetrasulfide ETP_4_3 (MIC < 0.1 μM), which was of interest because they are much poorer transporters.^10^ Further formal ring expansion leads to cyclic pentasulfides BPS_5_6 as equally outstanding inhibitors (MIC ≈ 0.3 μM). This trend toward the adaptive networks, reminiscent of elemental sulfur chemistry, did not extend toward inorganic polysulfides 13 (MIC ≈ 20 μM). ETPs 4 and 5 were sensitive to modification of the carboxylate, with the cationic 12 being the worst (MIC ≈ 30 μM) and the neutral glucose hemiacetal 7 the most promising (MIC ≈ 0.5 μM).

Although this study focuses on increasingly extreme dynamic covalent COC chemistry, the inclusion of one example for covalent C–S bond formation was of interest for comparison. The classical iodoacetamides^7^ and maleimides^4^ were more toxic than active (not shown). However, nucleophilic aromatic substitution of heteroaromatic sulfones,^20^ just developed for the efficient bioorthogonal conversion of thiols into sulfides, was more promising. Weaker than dynamic covalent COCs, this irreversible inhibition was best with benzoxazole 11 (MIC ≈ 15 μM) and decreased in accordance with reactivity toward free thiols to oxadiazole 14 and benzothiazole 21 (MIC ≈ 300 μM, Fig. 3e).

At constant pH, Ellman's reagent 20 was confirmed to be erratic also in this assay. The DRC showed minor inhibition up to around 2 mM, which disappeared again at higher concentrations (Fig. 3f). Other cyclic disulfides were inactive as well (28–30). Also disappointing were oxidized disulfides, that is thiosulfinates, including allicin 25, the main odorant component of garlic,^21,22^ oxidized cystine 26 and oxidized lipoic acid 27. Thiosulfinates were of interest because they should selectively target the vicinal thiols of reduced disulfides bridges, producing two disulfides.^23^ The most active *trans* dithioerythrol (DTE) thiosulfinate 17 isomerized with time into the less active, hydrogen-bonded *cis* isomer 22 (Fig. S46†).

Reporter 2 was more difficult to inhibit than 1, as expected from high activity with extreme retention on thiol affinity columns.^10,18^ For instance, BPS 6 was very efficient against ETP 1 but much less active against BPS 2 (Fig. 3a), although longer pre-incubation could lower the MIC down to 4 μM (Fig. 2, S41†). The complementary ETP 4 “self-inhibited” ETP 1 but was also unable to inhibit BPS 2 as efficiently (Fig. 3b). Among the best inhibitors of BPS 2 upon co-incubation were disulfide bridged γ-turn^24^ peptides 18 and 19 (MIC ≈ 5 μM), both less active against 1 (MIC ≈ 300 μM, Fig. 3c). Disulfide-bridged γ-turn CXC peptides consist of an 11-membered ring with significant Prelog strain. They were introduced by Wu and coworkers as transporters for efficient cytosolic delivery.^5^ The cyclic thiosulfonates 15 and 16 showed promising activities against both 1 and 2, and were tolerant toward the presence of serum (Fig. 2d, S33 and S42†). Contrary to thiosulfinate 27, the oxidation of lipoic acid to pure thiosulfonates was not successful so far. However, weakly detectable activity of the lipoyl-glutamate conjugate oxidized to the thiosulfinate (MIC ≈ 350 μM, not shown) compared to the inactive thiosulfinate 27 implied that lipoic acid oxidized to the thiosulfonate would also be less active than the glutamate conjugate 15.

The oxidized DTE 16^25–28^ was particularly intriguing because it was more potent against 2 and could achieve nearly complete inhibition (MIC ∼ 20 μM, Fig. 3d). Highly selective for thiols, the cyclic thiosulfonate 16 was stable for weeks at room temperature, without precaution, in all solvents tested. The disulfides and sulfinates obtained from exchange with thiols were stable as well, and the latter can further react with disulfides^27^ for intramolecular or eventually intermolecular crosslinking of the target proteins.

The overall mismatched inhibition profiles found for reporters 1 and 2 supported that thiol-mediated uptake proceeds through a series of at least partially uncoupled parallel multitarget systems instead of a specific single protein or membrane target. From proteomics studies with cysteine-reactive irreversible probes, it is known that different probes generally target different proteins.^29*b*^ Proteomics analysis^29*a*^ for asparagusic acid derived transporters supports the involvement of many targets beyond the commonly considered protein disulfide isomerases and the confirmed transferrin receptor.^12–14,26–30^ The unusual, formally anti-cooperative (Hill coefficients < 1) DRCs further supported thiol-mediated uptake as complex multitarget systems.

Despite the complexity of these systems, results did not much depend on assay conditions. Compared to the standard protocol of pre-incubation with inhibitors followed by inhibitor removal and incubation with reporters 1 or 2 for detection, the co-incubation protocol, in which pre-incubation with inhibitors is followed by co-incubation with reporters 1 or 2 without inhibitor removal, gave reasonably similar results (Fig. 2). Inhibition characteristics naturally depended on pre-incubation time, with weaker activities at shorter and longer times, reflecting incomplete exchange and cellular response or other ways of inhibitor destruction, respectively. The presence of serum also did not affect the activities much (Fig. 2b–d).

Preliminary studies on antiviral activity were performed with pseudo-lentivectors^31^ that express the D614G mutant^11^ of the SARS-CoV-2 spike protein and code for a luciferase reporter gene, which is expressed by the infected cells.^12^ A549 human lung alveolar basal epithelium cell line constitutively overexpressing ACE2 and TMPRSS2 was selected to facilitate the entry of the SARS-CoV-2 spike pseudo-lentivirus. The most significant activities were found for DTE thiosulfonate 16 with an IC_50_ around 50 μM, while toxicity was detected only at 500 μM (Fig. S44†). The onset of inhibition could be observed for tetrasulfide ETP 3 at 50 μM, but it coincided with the appearance of cytotoxicity. Protease inhibition is less likely to be the mode of action, as similar activity was found with wild type A549 cells transduced with a standard lentivirus expressing vesicular-stomatitis virus G surface protein VSVG (Fig. S45†).^13^ Short incubation times of cells and inhibitors before the addition of viruses disfavored contributions from changes in gene expression. More detailed studies are ongoing.

The lessons learned from this study are that, firstly, thiol-mediated uptake can be inhibited efficiently by thiol-reactive reagents, confirming that thiol-mediated uptake exists and transporters like ETP 1 and BPS 2 do not simply diffuse into cells; the best inhibitors are more than 5000 times better than Ellman's reagent. Secondly, inhibitor efficiencies vary with the transporters, supporting that thiol-mediated uptake operates as a complex multitarget system. The best inhibitors are COCs that operate with fast dynamic covalent exchange, suggesting that the reversibility provided by COCs is important. The inhibition of thiol-mediated uptake might contribute to activities of thiol-reactive antivirals such as 16, ETPs or ebselen, although they have been shown to bind to zinc fingers or inhibit proteases.^16,25,32–34^ Finally, the inhibitors reported here could also be of interest for delivery applications and might be worth investigation with regard to antiviral activity. We currently plan to focus more systematically on the most promising leads within COCs, particularly cyclic thiosulfonates, and to expand the screening campaign toward new attractive motifs.^33–35^

## Experimental section

See ESI.†

## Conflicts of interest

A U.S. Patent application has been filed (No. 63/073863).

## Supplementary Material

SC-012-D0SC05447J-s001
