# Supplementary material for: Inhibitors of thiol-mediated uptake
Source: Chem Sci. 2020 Nov 18;12(2):626–31. doi: 10.1039/d0sc05447j (PMC8179002; doi:10.1039/d0sc05447j)
Supplement: SC-012-D0SC05447J-s001 [file SC-012-D0SC05447J-s001.pdf]

Electronic Supplementary Material (ESI) for Chemical Science.
This journal is © The Royal Society of Chemistry 2020

Electronic Supplementary Information

Inhibitors of Thiol-Mediated Uptake

Yangyang Cheng, Anh-Tuan Pham, Takehiro Kato, Bumhee Lim, Dimitri Moreau, Javier López-Andarias, Lili Zong, Naomi Sakai, and Stefan Matile*

Department of Organic Chemistry, University of Geneva, Geneva, Switzerland

stefan.matile@unige.ch

Table of Contents

1. Materials and Methods S5

2. Synthesis S8

2.1. Synthesis of Reporters S8

2.2. Synthesis of Inhibitors S9

2.2.1. Synthesis of Disulfides S9

2.2.1.1. Synthesis of Epidiketodithiopiperazines (ETP) S9

2.2.1.2. Synthesis of Disulfide Bridged γ-Turn Peptides S15

2.2.1.3. Synthesis of Cyclic Disulfides S23

2.2.2. Synthesis of Benzopolysulfanes (BPS) S24

2.2.3. Synthesis of Heteroaromatic Sulfones S27

2.2.4. Synthesis of Thiosulfinates S28

2.2.5. Synthesis of Thiosulfonates S30

2.2.6. Synthesis of Inorganic Polysulfides (IPS) S33

3. Cell Culture S33

4. High-Content High-Throughput (HCHT) Inhibitor Screening S34

4.1. General Procedure for HCHT Inhibitor Screening S34

4.1.1. Pre-Incubation of Inhibitors S34

4.1.2. Co-Incubation of Inhibitors and Reporters S35

4.2. Data Analysis for HCHT Inhibitor Screening S36

4.3. HCHT Inhibitor Screening with ETP as Reporter S39

4.3.1. Screening with Disulfides as Inhibitors S40

4.3.1.1. Screening with ETP as Inhibitors S40

4.3.1.2. Screening with γ-Turn Peptides as Inhibitors S50

4.3.1.3. Screening with Cyclic Disulfides as Inhibitors S53

4.3.1.4. Screening with DTNB as Inhibitor S56

4.3.2. Screening with BPS as Inhibitors S57

4.3.3. Screening with Heteroaromatic Sulfones as Inhibitors S61

4.3.4. Screening with Thiosulfinates as Inhibitors S64

4.3.5. Screening with Thiosulfonates as Inhibitors S71

4.3.6. Screening with IPS as Inhibitor S74

4.4. HCHT Inhibitor Screening with BPS as Reporter S75

4.4.1. Screening with Disulfides as Inhibitors S76

4.4.1.1. Screening with ETP as Inhibitors S76

4.4.1.2. Screening with γ-Turn Peptides as Inhibitors S81

4.4.2. Screening with BPS as Inhibitors S84

4.4.3. Screening with Thiosulfonates as Inhibitors S85

5. Preliminary Tests on Antiviral Activity S87

6. Supplementary References S90

7. NMR Spectra S92

8. HPLC S116

1. Materials and Methods

As in ref. S1. Briefly, reagents for synthesis and commercially available final compounds were purchased from Sigma-Aldrich, Brunschwig, Alfa Aesar, Merck, TCI, Acros, and Iris Biotech. Phosphate buffered saline (PBS, pH = 7.4), DMEM (GlutaMAX, 4.5 g/L D-glucose, with phenol red) medium, FluoroBrite DMEM (high D-Glucose) medium, Penicillin-Streptomycin, Fetal Bovine Serum, TrypLE Express Enzyme and V96-MicroWell platen were obtained from Thermo Fisher Scientific. μ-Plate 96-Well Black were obtained from Ibidi. Hoechst 33342 (10 mg/mL solution in water) and propidium iodide (PI, 1.0 mg/mL solution in water) were obtained from Invitrogen by Thermo Fisher Scientific.

Column chromatography was carried out on silica gel (SiliaFlash® P60, SILICYCLE, 230 – 400 mesh). Analytical thin layer chromatography (TLC) was performed on silica gel 60 F254 (Merck). Reverse phase flash chromatography (column: SNAP Ultra C18 12 g) and hydrophilic interaction chromatography (column: BGB Scorpius Diol column 20 g) were performed on Biotage Isolera™ Four (eluents: CH_3CN and H_2O with 0.1% TFA). pH values were measured with a Consort C832 multi-parameter analyzer equipped with a VWR glass membrane pH electrode calibrated with Titrisol solution from Merck at pH 4.00 and 7.00. IR spectra were recorded on a Perkin Elmer Spectrum 100 FT-IR spectrometer (ATR, Golden Gate, unless stated) and are reported as wavenumbers v in cm^{-1} with band intensities indicated as br (broad), s (strong), m (medium), w (weak). ^{1}H and ^{13}C spectra were recorded (as indicated) either on a Bruker 300 MHz, 400 MHz or 500 MHz spectrometer and are reported as chemical shifts (δ) in ppm relative to TMS (δ = 0). Spin multiplicities are reported as a singlet (s), doublet (d), triplet (t) and quartet (q) with coupling constants (J) given in Hz, or multiplet (m). Broad peaks are marked as br. ^{1}H and ^{13}C resonances were assigned with the aid of additional information from 1D and 2D NMR spectra (H,H-NOESY, H,H-COSY, DEPT 135, HSQC and HMBC).

ESI-MS for the characterization of new compounds was performed on an ESI API 150EX and are reported as mass-per-charge ratio *m*/*z* (*m*/*z* intensity in %, [assignment]; mobile phase: $AcONH_4$ (2 mM) in MeOH). MALDI MS analysis for the characterization of new compounds was performed using Bruker MALDI Autoflex Speed TOF/TOF and is reported as mass-per-charge ratio *m/z* (matrix solution: DCTB (0.5 mg/0.2 mL) in $CHCl_3$). HR ESI-MS for the characterization of new compounds were performed on Xevo G2-S Tof (Waters) and are reported as mass-per-charge ratio *m/z* calculated and observed. HPLC was performed using JASCO LC-2000 Plus system equipped with quaternary pump (JASCO PU-2089) and UV/Vis detector (JASCO UV-2077 Plus, λ_{abs} at 210 nm for detection). The chromatographic column used was a Phenomenex Gemini 5u C18 110R column (150 × 3 mm, 5 µm particles size), flow 0.3 mL/min with the following conditions: 99% H_2O / 1% CH_3CN + 0.1% TFA from 0.00 to 5.00 min followed by a linear elution gradient from 99% H_2O / 1% CH_3CN + 0.1% TFA to 50% H_2O / 50% CH_3CN + 0.1% TFA from 5.00 min to 13.00 min. HPLC-MS were recorded using a Thermo Scientific Accela HPLC equipped with a Thermo C18 (5 cm x 2.1 mm, 1.9 µm particles) Hypersil gold column coupled with an LCQ Fleet three-dimensional ion trap mass spectrometer (ESI, Thermo Scientific) with a linear elution gradient from 95% H_2O / 5% CH_3CN + 0.01% TFA to 10% H_2O / 90% CH_3CN + 0.01% TFA in 4.0 min at a flow rate of 0.75 mL/min. Retention times (R_t) are reported in minutes. Fluorescence cellular imaging was performed using an IXM-C automated microscope from ImageXpress equipped with a Lumencor Aura III with 5 independently selectable solid-state light sources, bandpass filters and 5 objectives (4x to 60x). Sample preparation and washing on µ-Plate 96-Well Black was performed using a Plate washer Biotek EL406®.

Abbreviations. ACE2: Angiotensin converting enzyme 2; AspA: Asparagusic acid; Boc_2O: Di-*tert*-butyl dicarbonate; BPS: Benzopolysulfane; COCs: Cyclic oligochalcogenides;

DCC: *N*,*N*'-Dicyclohexylcarbodiimide; DCTB: *trans*-2-[3-(4-*tert*-Butylphenyl)-2-methyl-2-propenylidene]malononitrile; DIAD: Diisopropyl azodicarboxylate; DMAP: 4-Dimethylaminopyridine; DMEM: Dulbecco's modified eagle medium; DMF: *N*,*N*-Dimethylformamide; DMSO: Dimethyl sulfoxide; DRCs: Dose response curves; DTNB: 5,5'-Dithiobis(2-nitrobenzoic acid); DTT: 1,4-Dithio-DL-threitol; EDCI: 1-Ethyl-3-(3-dimethylaminopropyl)carbodiimide hydrochloride; EtOAc: Ethyl acetate; ETP: Epidithiodiketopiperazine; FBS: Fetal bovine serum; GI_{50}: Concentration causing 50% cell growth inhibition; HCHT: High-content high-throughput; HILIC: Hydrophilic interaction chromatography; IC_{50}: Half maximal inhibitory concentration; LA: Lipoic acid; *m*-CPBA: *meta*-Chloroperoxybenzoic acid; MICs: Minimum inhibitory concentrations; NHS: *N*-Hydroxysuccinimide; PBS: Phosphate buffer saline; PE: Petroleum ether; PS: Penicillin/Streptomycin; rt: Room temperature; RV: Relative viability; TEA: Triethylamine; TFA: Trifluoroacetic acid; THF: Tetrahydrofuran; TMPRSS2: Transmembrane protease serine 2; TMSCl: Trimethylsilyl chloride; VSVG: Vesicular-somatitis virus G surface protein.

2. Synthesis

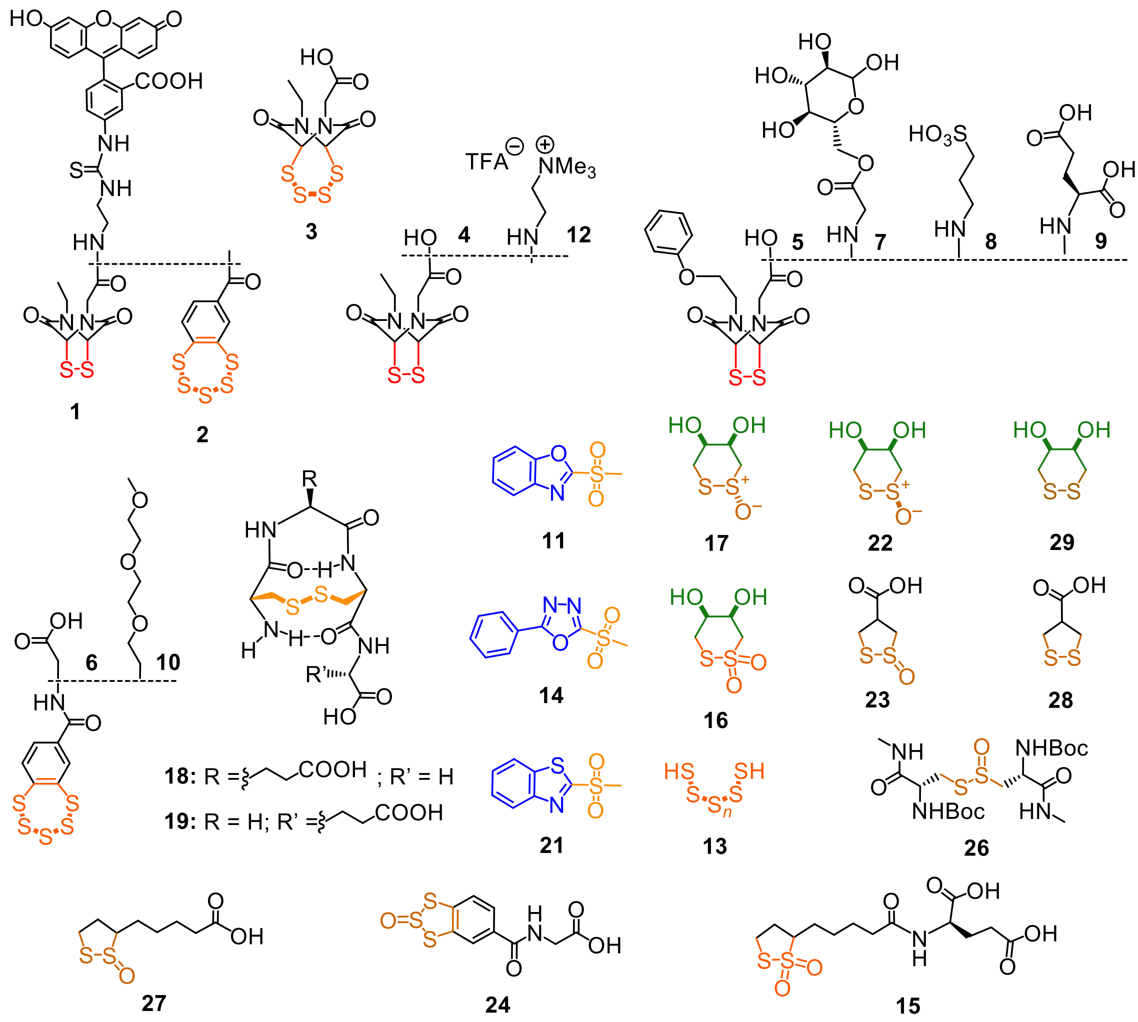

Fig. S1 Structure of synthetic reporters and inhibitor candidates.

2.1. Synthesis of Reporters

Compound 1 was synthesized and purified according to procedures described in ref. S2.

Compound 2 was synthesized and purified according to procedures described in ref. S3.

2.2. Synthesis of Inhibitors

2.2.1. Synthesis of Disulfides

2.2.1.1. Synthesis of Epidiketodithiopiperazines (ETP)

Compound 3 (racemic) was synthesized and purified according to procedures described in ref. S3.

Compound 4 (racemic) was synthesized and purified according to procedures described in ref. S2.

Compound 5 (racemic) was synthesized and purified according to procedures described in ref. S3.

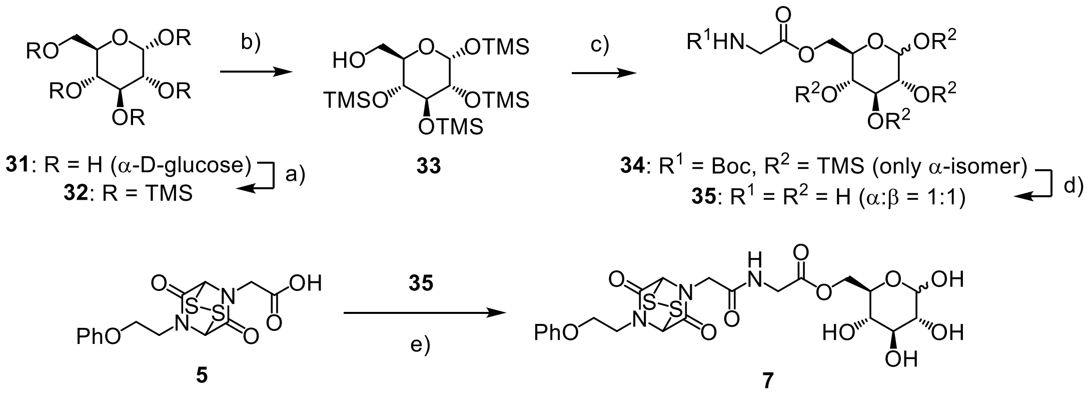

Scheme S1 (a) TMSCl, Et_3N, DMF, rt, 24 h, 96%; (b) NH_4OAc, CH_2Cl_2/MeOH, rt, 25 h, 65%; (c) Boc-Gly-OH, DIAD, PPh_3, THF, rt, 19 h; 40 °C, 24 h, 24%; (d) TFA, CH_2Cl_2, rt, 3.5 h; (e) 1. NHS, DCC, THF, rt, 16 h; 2. **35**, DMF, 32 h, 55% (2 steps from **34**).

Compound 32 was synthesized and purified according to procedures described in ref. S4.

Compound 33 was synthesized and purified according to procedures described in ref. S5.

Compound 34. To a solution of **33** (201 mg, 0.429 mmol), Boc-Gly-OH (184 mg, 1.05 mmol) and PPh_3 (255 mg, 0.972 mmol) in THF (5.0 mL), DIAD (0.20 mL, 1.0 mmol) was added dropwise at 0 °C. After the addition, the mixture was warmed to rt and stirred for 19 h. Then, the mixture was stirred for another 24 h at 40 °C. The mixture was diluted with CH_2Cl_2, washed with $NaHCO_3$ aq. (x2) and brine, dried over Na_2SO_4, filtered, and concentrated under vacuum. The residue was purified by flash column chromatpgraphy (SiO_2, pentane/CH_2Cl_2 5:1 – CH_2Cl_2, R_f (CH_2Cl_2): 0.19) to yield **34** (74.5 mg, 24%) as a colorless oil. $[\alpha]_D^{20}$ +68.2 (*c* 1.00, $CHCl_3$); IR (neat): 2959 (m), 1704 (m), 1514 (m), 1251 (s), 1161 (s), 1081 (s), 972 (m), 844 (s), 752 (m); ^{1}H NMR (400 MHz, CD_2Cl_2): 5.07 – 4.96 (m, 2H), 4.42 (dd, $^2J_{H–H}$ = 11.6, $^3J_{H–H}$ = 2.3 Hz, 1H), 4.07 (dd, $^2J_{H–H}$ = 11.6, $^3J_{H–H}$ = 5.3 Hz, 1H), 3.98 – 3.81 (m, 3H), 3.77 (dd, $^3J_{H–H}$ = 8.9, 8.9 Hz, 1H), 3.46 – 3.32 (m, 2H), 1.43 (s, 9H), 0.16 (s, 9H), 0.15 (s, 9H), 0.15 (s, 9H), 0.14 (s, 9H); ^{13}C NMR (100 MHz, CD_2Cl_2): 170.6 (C), 155.9 (C), 94.3 (CH), 80.1 (C), 74.30 (CH), 74.26 (CH), 72.7 (CH), 70.3 (CH), 64.8 (CH_2), 42.8 (CH_2), 28.4 (CH_3), 1.4 (CH_3), 1.0 (CH_3), 0.5 (CH_3), 0.2 (CH_3); MS (ESI, MeOH): 643 (100, $[M+NH_4]^+$).

Compound 7. To a solution of **34** (10.1 mg, 16.1 μmol) in CH_2Cl_2 (0.75 mL), TFA (0.75 mL) was added. After 3.5 h, the mixture was concentrated under vacuum. MeOH and H_2O was added to the mixture, and the resultant mixture was lyophilized overnight. The residue was dissolved in DMF (0.10 mL), and the solution of **35** was used directly in the next step.

To a solution of **5** (7.2 mg, 20 μmol) and NHS (3.4 mg, 30 μmol) in THF (1.3 mL), DCC (5.7 mg, 28 μmol) was added under N_2 and the mixture was stirred at rt for 18 h. The mixture was filtered to remove precipitates of the urea, and the solution of **35** was added to the filtrate. The resultant mixture was then stirred at rt for another 32 h. THF was removed under vacuum

at 25 °C. The residue was purified by reverse phase flash chromatography (Eluent: H_2O + 0.1% TFA/CH_3CN + 0.1% TFA gradient from 100:0 to 50:50) to yield **7** (4.7 mg, 55% from **34**, mixture of 4 stereoisomers) as a colorless solid. IR (neat): 3318 (br), 1741 (w), 1672 (s), 1598 (w), 1447 (w), 1411 (w), 1198 (s), 1032 (s), 755 (w), 691 (w); ^{1}H NMR (500 MHz, CD_3OD, α- and β-OH anomers): 7.29 – 7.23 (m, 2H, α/β), 6.98 – 6.90 (m, 3H, α/β), 5.99– 5.97 (m, 1H, α/β), 5.83 – 5.79 (m, 1H, α/β), 5.08 (d, $^3J_{H–H}$ = 3.9 Hz, 1H, β), 4.57 – 4.51 (m, 1H, α/β), 4.48 (m, 1H, α), 4.45/4.40/4.28-4.23 (dt, $^2J_{H–H}$ = 11.6, $^3J_{H–H}$ = 2.1 Hz/ddd, $^2J_{H–H}$ = 11.6, $^3J_{H–H}$ = 4.3, 2.3 Hz/m, 2H, α/β), 4.25-4.19/4.16 (m/ddd, $^2J_{H–H}$ = 10.2, $^3J_{H–H}$ = 8.0, 4.1 Hz, 2H, α/β), 4.04 (d, $^2J_{H–H}$ = 16.5 Hz, 1H, α/β), 4.01 – 3.87 (m, 4H), 3.99 – 3.95 (m, 1H, α), 3.69 – 3.63 (m, 1H, α), 3.49 (ddd, $^3J_{H–H}$ = 9.6, 5.8, 2.1 Hz, 1H, β), 3.38 – 3.35 (m, 1H, α), 3.35 – 3.32 (m, 1H, β), 3.30 – 3.25 (m, 1H, α/β), 3.14 (dd, $^3J_{H–H}$ = 9.2, 7.7 Hz, 1H, β); ^{13}C NMR (125 MHz, CD_3OD, α- and β-OH anomers): 171.1/171.0 (C, α/β), 169.9 (C, α/β), 166.1 (C, α/β), 165.9 (C, α/β), 159.9 (C, α/β), 130.5 (CH, α/β), 122.3 (CH, α/β), 115.8 (CH, α/β), 98.3 (CH, β), 94.0 (CH, α), 77.9 (CH, β), 76.2 (CH, β), 75.1 (CH, β), 74.7 (CH, α), 73.8/73.7 (CH, α), 71.84/71.81/71.6/71.5 (CH, α/β), 70.6/70.5 (CH, α), 67.2 (CH, α/β), 67.1 (CH, α/β), 66.9 (CH_2, α/β), 65.72/65.68/65.6/65.5 (CH_2, α/β), 46.4 (CH_2, α/β), 45.3 (CH_2, α/β), 42.1 (CH_2, α/β); MS (ESI, MeOH): 596 (100, $[M+Na]^+$).

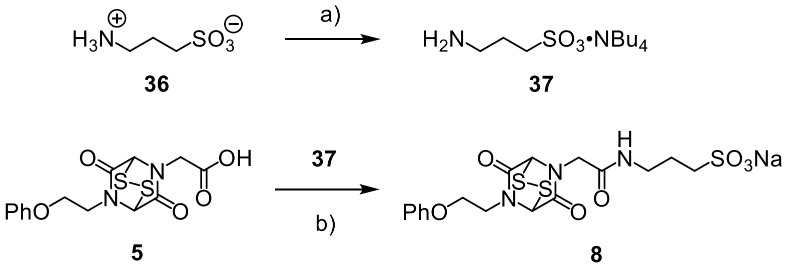

Scheme S2 (a) Bu_4NOH, MeOH, rt; (b) 1. NHS, DCC, THF, rt, 16 h; 2. **37**, rt, 30 min, 37% from **36**.

Compound 8. To a suspension of **36** (13.9 mg, 99.9 μmol) in MeOH, Bu_4NOH (1.0 M in MeOH, 90 μL, 90 μmol) was added. The mixture was sonicated until **36** was completely dissolved. The resultant solution was concentrated, and the crude **37** was dissolved in DMF (0.20 mL), which was used directly in the next step.

To a solution of **5** (7.2 mg, 20 μmol) and NHS (4.0 mg, 35 μmol) in THF (1.3 mL), DCC (5.9 mg, 29 μmol) was added under N_2 and the mixture was stirred at rt overnight. The mixture was filtered to remove precipitates of the urea, and the solution of **37** (32 μL, 16 μmol) was added to the filtrate. After 30 min, the mixture was concentrated under vacuum at 25 °C. The residue was purified by reverse phase flash chromatography (Eluent: H_2O + 0.1% TFA/CH_3CN + 0.1% TFA gradient from 100:0 to 40:60) to yield **8** (2.8 mg, 37% from **36**) as a colorless solid. Mp: 104-106 °C; IR (neat): 3274 (br, w), 2963 (w), 1687 (s), 1444 (w), 1221 (m), 1146 (m), 1031 (m), 752 (w), 688 (w); ^{1}H NMR (500 MHz, CD_3OD): 7.29 – 7.23 (m, 2H), 6.99 – 6.90 (m, 3H), 5.96 (s, 1H), 5.83 (s, 1H), 4.42 (d, $^2J_{H-H}$ = 16.5 Hz, 1H), 4.23 (dt, $^2J_{H-H}$ = 9.7, $^3J_{H-H}$ = 4.5 Hz, 1H), 4.17 (ddd, $^2J_{H-H}$ = 9.7, $^3J_{H-H}$ = 7.7, 4.0 Hz, 1H), 4.02 – 3.87 (m, 3H), 3.32 (m, 2H, overlapped with solvent peak; deduced from HSQC), 2.86 – 2.80 (m, 2H), 2.00 – 1.93 (m, 2H); ^{13}C NMR (125 MHz, CD_3OD): 169.2 (C), 166.1 (C), 165.8 (C), 159.9 (C), 130.5 (CH), 122.2 (CH), 115.8 (CH), 67.33 (CH), 67.27 (CH), 66.9 (CH_2), 50.1 (CH_2), 46.7 (CH_2), 45.3 (CH_2), 39.5 (CH_2), 26.0 (CH_2); MS (ESI, MeOH): 476 (100, $[M+H]^+$).

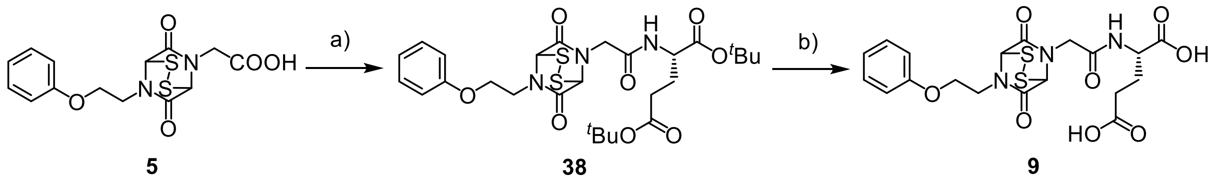

Scheme S3 (a) L-glutamic acid di-*tert*-butyl ester hydrochloride, EDCI, TEA, CH_2Cl_2, rt, overnight, 71%; (b) TFA/CH_2Cl_2 (1:1), rt, 1 h, 91%.

Compound 38. To a solution of **5** (30 mg, 0.080 mmol) in CH_2Cl_2 (800 µL), L-glutamic acid di-*tert*-butyl ester hydrochloride (22 mg, 0.11 mmol), EDCI (22 mg, 0.11 mmol) and TEA (3.0 µl, 0.040 mmol) were added. The reaction mixture was stirred at rt overnight. The solvent was removed under vacuum. The residue was purified by reversed phase flash chromatography (Eluent: H_2O + 0.1% TFA/CH_3CN + 0.1% TFA gradient from 80:20 to 5:95) to yield **38** as a colorless solid (36 mg, 71%, mixture of 2 diastereomers (1:1)). Mp: 54 – 55 °C; IR (neat): 3324 (w), 2981 (w), 2935 (w), 1688 (s), 1600 (w), 1535 (w), 1497 (w), 1441 (w), 1414 (w), 1393 (w), 1368 (w), 1299 (w), 1225 (m), 1148 (s), 1082 (w), 1033 (w), 953 (w), 907 (w), 844 (w), 813 (w), 787 (w), 754 (w), 692 (w), 673 (w), 615 (w); ^{1}H NMR (400 MHz, $CDCl_3$, nn/nn-50:50 diastereomeric peaks, some diastereomeric peaks overlap): 7.31 – 7.26 (m, 2H), 7.01 – 6.95 (m, 1H), 6.94 – 6.88 (m, 2H), 6.77 – 6.64 (m, 1H), 5.78/5.76 (s, 1H), 5.55/5.54 (s, 1H), 4.48 – 4.43 (m, 1H), 4.36 – 4.08 (m, 4H), 4.03 – 3.87 (m, 2H), 2.38 – 2.20 (m, 2H), 2.15 – 2.03 (m, 1H), 1.95 – 1.76 (m, 1H), 1.48 – 1.46 (m, 9H), 1.44 – 1.42 (m, 9H); ^{13}C NMR (100 MHz, $CDCl_3$, nn/nn-diastereomeric peaks, some diastereomeric peaks overlap): 172.3/172.2 (C), 170.5/170.3 (C), 166.5/166.4 (C), 164.0/163.8 (C), 163.3/163.2 (C), 157.9 (C), 129.6 (2xCH), 121.6 (CH), 114.6/114.5 (2xCH), 82.8 (C), 81.01/80.96 (C), 66.4/66.3 (CH), 66.2/66.1 (CH_2), 66.0/65.9 (CH), 52.7/52.6 (CH), 47.7/47.0 (CH_2), 44.33/44.31 (CH_2), 31.4/31.2 (CH_2), 28.10/28.06 (3xCH_3), 27.99/27.96 (3xCH_3), 27.40/27.37 (CH_2); MS (ESI, MeOH): 484 (100, [M-2*t*-Bu+H]$^+$).

Compound 9. Substrate **38** (36.0 mg, 0.063 mmol) was dissolved in CH_2Cl_2/TFA (1.0 mL : 1.0 mL). The reaction mixture was stirred at rt for 1 h. The solvent was removed under vacuum. The residue was purified by reversed phase flash chromatography (Eluent: H_2O + 0.1% TFA/CH_3CN + 0.1% TFA gradient from 95:5 to 30:70) to yield the desired product **9** as a colorless solid (28.0 mg, 91%, mixture of 2 diastereomers). Mp: 181 – 182 °C (decomp.); IR

(neat): 3304 (br), 2988 (br), 2608 (br), 1677 (s), 1599 (m), 1544 (m), 1493 (m), 1442 (m), 1412 (m), 1363 (w), 1336 (w), 1298 (w), 1227 (s), 1191 (s), 1175 (s), 1082 (w), 1033 (w), 953 (w), 906 (w), 815 (w), 788 (w), 755 (m), 691 (w), 673 (w), 614 (w); ^{1}H NMR (400 MHz, CD_3OD, nn/nn-50:50 diastereomeric peaks, some diastereomeric peaks overlap): 7.32 – 7.18 (m, 2H), 7.05 – 6.85 (m, 3H), 5.97/5.97 (s, 1H), 5.81/5.81 (s, 1H), 4.59 – 4.36 (m, 2H), 4.28 – 4.12 (m, 2H), 4.10 – 3.83 (m, 3H), 2.48 – 2.31 (m, 2H), 2.23 – 2.11 (m, 1H), 2.03 – 1.86 (m, 1H); ^{13}C NMR (125 MHz, CD_3OD, nn/nn-50:50 diastereomeric peaks, some diastereomeric peaks overlap): 175.1/174.9 (C); 173.6/173.3 (C); 167.9/167.8 (C), 164.66/164.65 (C), 164.44/164.41, 158.5 (C), 129.1 (2xCH), 120.8 (CH), 114.4 (2xCH), 65.91/65.90 (CH), 65.75/65.73 (CH), 65.5 (CH_2), 52.0 (CH), 45.11/45.14 (CH_2), 43.8 (CH_2), 29.8 (CH_2), 26.72/26.65 (CH_2); MS (ESI, MeOH): 484 (100, $[M+H]^+$).

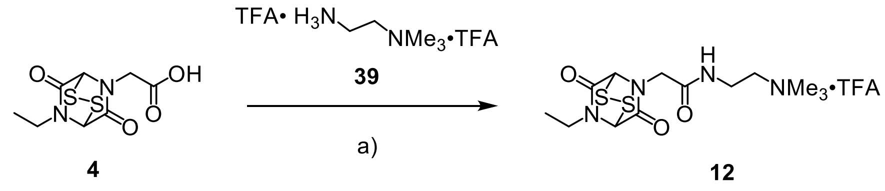

Scheme S4 (a) 1. NHS, DCC, THF/DMF, rt; 2. **39**, DMF, rt, 19%.

Compound 39 was synthesized and purified according to procedures described in ref. S6.

Compound 12. To a solution of **4** (12.9 mg, 0.0492 mmol) and NHS (7.6 mg, 0.066 mmol) in a 3:1 mixture solvent of THF and DMF (1.0 mL) in a 2-necked round bottom flask under N_2 atmosphere, DCC (13.0 mg, 0.0630 mmol) was added. After the reaction was stirred under N_2 for 15 h, the mixture was passed through a cotton plug. Then, a solution of **39** (0.12

M in THF/DMF 3:1, 0.33 mL, 0.040 mmol) was added to the filtrate, and the mixture was stirred for 3 h, followed by the removal of THF under vacuum at 25 °C. The residue was purified by reverse phase flash chromatography twice (Eluent (first time): H_2O + 0.1% TFA/CH_3CN + 0.1% TFA gradient from 80:20 to 25:75; Eluent (second time): H_2O + 0.1 % TFA/MeCN + 0.1 % TFA gradient from 100:0 to 50:50) to yield **12** (3.3 mg, 19%) as a colorless amorphous solid. IR (neat): 3290 (br, w), 2925 (w), 1658 (s), 1427 (m), 1268 (m), 1171 (s), 1120 (s), 951 (w), 829 (w), 799 (w), 718 (w); ^{1}H NMR (300 MHz, CD_3OD): 8.46 (s, N-H), 5.88 (s, 1H), 5.76 (s, 1H), 4.42 (d, $^2J_{H–H}$ = 16.6 Hz, 1H), 4.02 (d, $^2J_{H–H}$ = 16.6 Hz, 1H), 3.81 – 3.52 (m, 4H), 3.48 (t, $^3J_{H–H}$ = 6.6 Hz, 2H), 3.18 (s, 9H), 1.28 (t, $^3J_{H–H}$ = 7.2 Hz, 3H); ^{13}C NMR (125 MHz, CD_3OD): 168.4 (C) 164.6 (C), 164.1 (C), 66.0 (CH), 64.4 (CH), 64.1 (CH_2), 52.4 (t, $^1J_{C–N}$ = 3.8 Hz, CH_3), 45.5 (CH_2), 39.8 (CH_2), 33.3 (CH_2), 11.5 (CH_3); ^{19}F NMR (282 MHz, CD_3OD): -77.09; MS (ESI, H_2O/MeCN): 174 (100, $[M\text{-}TFA+H]^{2+}$).

2.2.1.2. Synthesis of Disulfide Bridged γ-Turn Peptides

General procedure A (removal of Fmoc protecting group):

A solution of Fmoc-protected peptide (or amino acid, 0.2 M) in a mixture of diethylamine/CH_2Cl_2 1:1 was stirred at rt for 1 h. The solvent was removed *in vacuo*. The residue was dried under vacuum for 30 min and then used for the coupling reaction without further purification.

General procedure B (amide formation with EDCI):

To a 0.1 M solution of *N*-deprotected peptide (or amino acid) in CH_2Cl_2, DMAP (1.2 equiv.) was added, followed by *N*-Fmoc-amino acid-OH (or *N*-Boc-amino acid-OH) (1.2 equiv.) and EDCI (1.2 equiv.). The mixture was stirred at rt overnight. The reaction mixture

was then diluted with CH_2Cl_2 and washed with 10% aqueous solution of citric acid, water, saturated aqueous solution of $NaHCO_3$ and brine. The organic phase was dried over Na_2SO_4 and concentrated *in vacuo*. The residue was purified by flash chromatography (SiO_2, CH_2Cl_2/MeOH) to yield the desired product.

General procedure C (cyclization of peptide):

To a 2.8 mM solution of I_2 (2.0 equiv.) in methanol, 3.5 mM solution of peptide (1.0 equiv.) in methanol was added dropwise. The reaction mixture was stirred at rt for another 4 h. Then the reaction mixture was diluted with CH_2Cl_2. The organic solution was washed 3 times with 10% aqueous solution of KI to remove the excess I_2. The organic layer was dried over Na_2SO_4, filtered and concentrated *in vacuo*. The residue was purified by flash chromatography (SiO_2, CH_2Cl_2/MeOH) to yield the desired product.

General procedure D (removal of Boc and *t*-Bu protecting group with HCl gas):

HCl gas was bubbled through a solution of the protected peptide in EtOAc during 1 h stirring at rt. Then the solvent was removed *in vacuo*. The residue was triturated with Et_2O 3 times, dried under vacuum and purified by HILIC flash chromatography (Eluent: H_2O + 0.1% TFA/CH_3CN + 0.1% TFA gradient from 5:95 to 95:5). Fractions containing the product were lyophilized to yield the desired product.

General procedure E (removal of Boc and *t*-Bu protecting group with TFA):

The protected peptide was dissolved in a mixture of CH_2Cl_2/TFA (1:1) (more than 20 equiv. of TFA per *t*-Bu or Boc protecting groups). The reaction mixture was stirred overnight at rt. The solvent was then removed *in vacuo*. The residue was triturated with Et_2O 3 times, dried under vacuum and purified by HILIC flash chromatography (Eluent: H_2O + 0.1% TFA/CH_3CN + 0.1% TFA gradient from 5:95 to 95:5) or reversed phase flash chromatography

(Eluent: H_2O + 0.1% TFA/CH_3CN + 0.1% TFA gradient from 5:95 to 95:5). Fractions containing the product were lyophilized to yield the desired product.

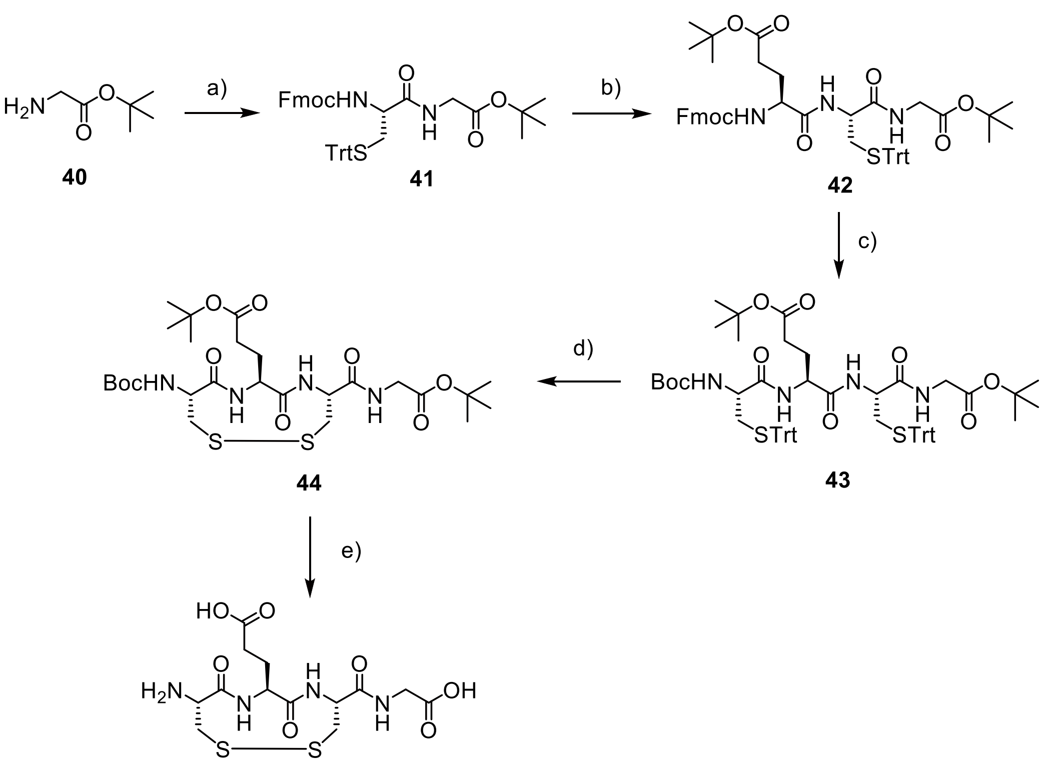

Scheme S5 (a) Fmoc-L-Cys(Trt)-OH, EDCI, DMAP, CH_2Cl_2, rt, overnight, 84%; (b) 1. Et_2NH/CH_2Cl_2 1:1, rt, 1 h; 2. Fmoc-L-Glu(*t*-Bu)-OH, EDCI, DMAP, CH_2Cl_2, rt, overnight, 52%; (c) 1. Et_2NH/CH_2Cl_2 1:1, rt, 1 h; 2. Boc-L-Cys(*t*-Bu)-OH, EDCI, DMAP, CH_2Cl_2, rt, overnight, 67%; (d) I_2, MeOH, 4 h, rt, 39%; (e) HCl gas, EtOAc, rt, 1 h, 96%.

Compound 41 (0.9 g, 84%, colorless solid) was prepared from **40** (0.20 g, 1.5 mmol) and Fmoc-L-Cys(Trt)-OH (1.1 g, 1.8 mmol) following the general procedure B. R_f (CH_2Cl_2/MeOH 25:1): 0.68. Spectroscopic data were consistent with those reported in ref. S7.

Compound 42 (1.0 g, 52%, colorless solid) was prepared from **41** (1.5 g, 2.1 mmol) and Fmoc-L-Glu(*t*-Bu)-OH (1.1 g, 2.5 mmol) following the general procedures A and B. R_f (CH_2Cl_2/MeOH 25:1): 0.46; Mp: 99 – 100 °C; $[\alpha]_D^{20}$ -2.31 (*c* 1.00, $CHCl_3$); IR (neat): 3288 (w), 2877 (w), 1725 (m), 1650 (m), 1515 (w), 1446 (w), 1367 (w), 1226 (m), 1152 (s), 1082 (w), 1036 (w), 841 (w), 740 (m), 700 (m), 620 (w); ^{1}H NMR (400 MHz, $CDCl_3$): 7.77 (d, $^3J_{H–H}$ = 7.5 Hz, 2H), 7.61 – 7.57 (m, 2H), 7.44 – 7.38 (m, 8H), 7.32 – 7.27 (m, 4H), 7.26 – 7.24 (m, 2H), 7.21 – 7.16 (m, 3H), 6.70 (br, 1H), 6.61 (d, $^3J_{H–H}$ = 8.0 Hz, 1H), 5.99 (d, $^3J_{H–H}$ = 6.6 Hz, 1H), 4.45 – 4.31 (m, 2H), 4.24 – 4.07 (m, 3H), 3.99 – 3.86 (m, 1H), 3.81 – 3.69 (m, 1H), 2.89 – 2.75 (m, 1H), 2.61 (dd, $^2J_{H–H}$ = 13.0 Hz, $^3J_{H–H}$ = 5.2 Hz, 1H), 2.51 – 2.27 (m, 2H), 2.14 – 2.02 (m, 1H), 1.99 – 1.86 (m, 1H), 1.46 – 1.39 (m, 18H); ^{13}C NMR (100 MHz, $CDCl_3$): 173.3 (C), 171.2 (C), 169.6 (C), 156.5 (C), 144.4 (3xC), 143.7 (2xC), 141.3 (2xC), 129.6 (6xCH), 128.1 (6xCH), 127.8 (3xCH), 127.1 (2xCH), 126.9 (2xCH), 125.2 (2xCH), 120.0 (2xCH), 82.1 (C), 81.3 (C), 67.3 (CH_2), 65.2 (C), 55.0 (CH), 53.5 (CH), 52.2 (CH), 47.1 (CH), 42.1 (CH_2), 33.3 (CH_2), 31.8 (CH_2), 28.1 ($3xCH_3$), 28.0 ($3xCH_3$), 27.5 (CH_2); MS (MALDI-TOF): 906 ($[M+Na]^+$).

Compound 43 (831 mg, 67%, colorless solid) was prepared from **42** (987 mg, 1.12 mmol) and Boc-L-Cys(Trt)-OH (622 mg, 1.34 mmol) following the general procedures A and B. R_f (CH_2Cl_2/MeOH 25:1): 0.44; Mp: 174 – 175 °C (decomp.); $[\alpha]_D^{20}$ +2.65 (*c* 1.00, $CHCl_3$); IR (neat): 3286 (w), 2977 (w), 2929 (w), 1717 (m), 1645 (m), 1491 (m), 1445 (w), 1391 (w), 1367 (w), 1247 (w), 1153 (s), 1034 (w), 846 (w), 743 (s), 699 (s), 675 (w), 619 (w); ^{1}H NMR (400 MHz, $CDCl_3$): 7.49 (d, $^3J_{H–H}$ = 5.9 Hz, 1H), 7.42 – 7.36 (m, 12H), 7.32 – 7.26 (m, 8H), 7.26 – 7.23 (m, 4H), 7.23 – 7.16 (m, 6H), 6.89 (d, $^3J_{H–H}$ = 8.3 Hz, 1H), 6.76 (br, 1H), 4.85 (d, $^3J_{H–H}$ = 6.1 Hz, 1H), 4.23 – 4.13 (m, 1H), 4.08 – 3.98 (m, 1H), 3.89 – 3.78 (m, 2H), 3.77 – 3.68 (m, 1H), 2.78 – 2.64 (m, 3H), 2.53 (dd, $^2J_{H–H}$ = 12.9 Hz, $^3J_{H–H}$ = 4.8 Hz, 1H), 2.47 – 2.37 (m,

1H), 2.35 – 2.24 (m, 1H), 2.08 – 1.98 (m, 1H), 1.98 – 1.86 (m, 1H), 1.45 – 1.41 (m, 18H), 1.40 (s, 9H); ^{13}C NMR (100 MHz, $CDCl_3$): 173.7 (C), 171.4 (C), 170.8 (C), 169.7 (C), 168.4 (C), 155.8 (C), 144.6 (3xC), 144.2 (3xC), 129.6 (6xCH), 129.5 (6xCH), 128.2 (6xCH), 128.0 (6xCH), 127.0 (3xCH), 126.8 (3xCH), 81.8 (C), 81.2 (C), 80.7 (C), 67.30 (C), 67.28 (C), 54.4 (CH), 53.9 (CH), 52.8 (CH), 42.1 (CH_2), 33.3 (CH_2), 33.1 (CH_2), 31.8 (CH_2), 28.3 ($3xCH_3$), 28.1 ($6xCH_3$), 26.5 (CH_2); MS (MALDI-TOF): 1130 ($[M+Na]^+$).

Compound 44 (122 mg, 39%, colorless solid) was prepared from **43** (557 mg, 0.503 mmol) following the general procedure C. R_f (CH_2Cl_2/MeOH 25:1): 0.24; Mp: 158 – 159 °C; $[\alpha]_D^{20}$ -2.29 (*c* 1.00, $CHCl_3$); IR (neat): 3213 (w), 2977 (w), 1660 (s), 1516 (m), 1367 (m), 1248 (m), 1153 (s), 1052 (w), 847 (w), 754 (w); ^{1}H NMR (400 MHz, $CDCl_3$): 7.96 – 7.64 (m, 2H), 7.11 (br, 1H), 5.56 (br, 1H), 4.80 – 4.62 (m, 1H), 4.62 – 4.50 (m, 1H), 4.50 – 4.35 (m, 1H), 4.02 – 3.86 (m, 2H), 3.68 – 3.10 (br, 3H), 3.09 – 2.79 (m, 1H), 2.51 – 2.33 (m, 2H), 2.21 – 1.99 (m, 2H), 1.46 (s, 9H), 1.44 – 1.42 (m, 18H); ^{13}C NMR (100 MHz, $CDCl_3$): 173.1 (C), 172.4 (C), 169.1 (2xC), 168.5 (C), 154.7 (C), 82.3 (C), 81.4 (C), 80.4 (C), 54.5 (CH), 53.4 (CH), 52.9 (CH), 44.7 ($2xCH_2$), 42.0 (CH_2), 31.7 (CH_2), 28.3 ($3xCH_3$), 28.09 ($3xCH_3$), 28.07 ($3xCH_3$), 23.7 (CH_2); MS (ESI, MeOH): 620 (100, $[M+H]^+$).

Compound 18 (84 mg, 96%, colorless solid) was prepared from **44** (122 mg, 0.197 mmol) following the general procedure D. Mp: 234 – 235 °C (decomp.); $[\alpha]_D^{20}$ -51 (*c* 0.50, MeOH); IR (neat): 2938 (m), 1658 (s), 1531 (m), 1408 (m), 1208 (m), 1039 (w), 855 (w), 664 (w); ^{1}H NMR (500 MHz, CD_3OD): 4.72 – 4.30 (m, 2H), 4.12 (m, 1H), 3.97 – 3.76 (m, 2H), 3.68 – 3.26 (m, 2H), 3.19 – 2.71 (m, 2H), 2.45 – 2.28 (m, 2H), 2.19 – 2.05 (m, 1H), 1.96 – 1.83 (m, 1H); ^{13}C NMR (125 MHz, CD_3OD): 173.5 (C), 171.3 (C), 170.3 (C), 170.2 (C), 169.0 (C), 53.0 (CH), 52.6 (CH), 52.2 (CH), 40.5 ($2xCH_2$), 40.4 (CH_2), 29.4 (CH_2), 23.0 (CH_2); HRMS (ESI, +ve) calcd for $C_{13}H_{20}N_4O_7S_2$ ($[M+H]^+$): 409.0847, found: 409.0846.

Note: Peaks are broad in NMR spectra due to the presence of conformers as described for similar compounds in ref. S8.

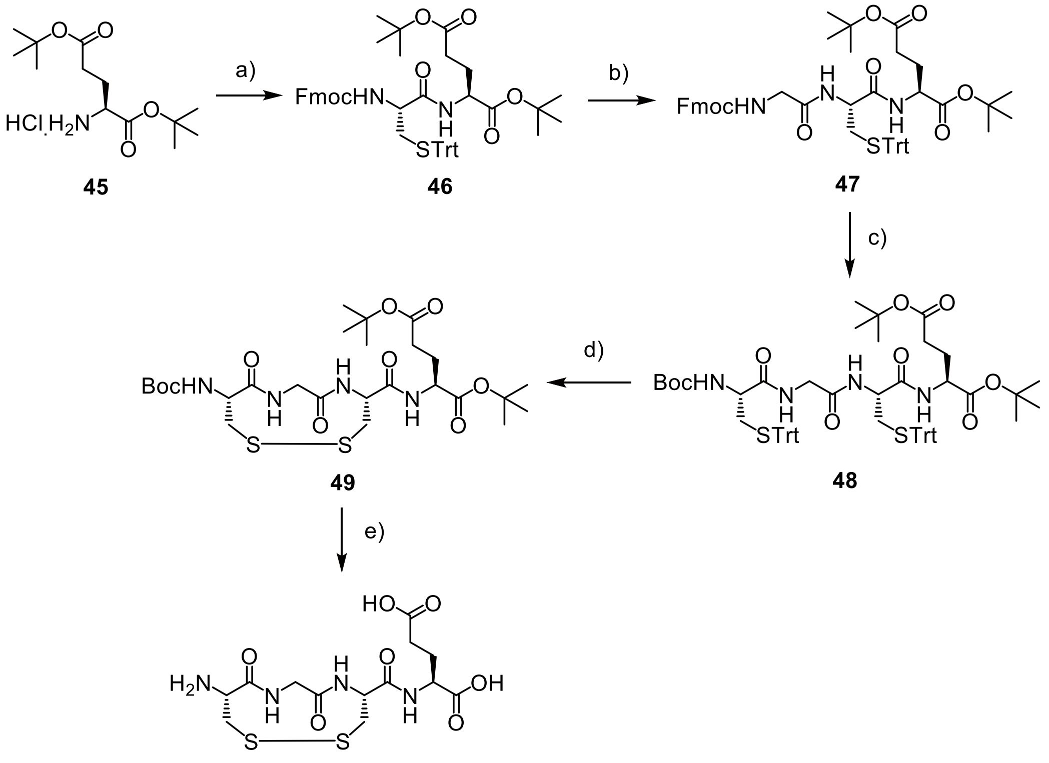

Scheme S6 (a) Fmoc-L-Cys(Trt)-OH, EDCI, DMAP, CH_2Cl_2, rt, overnight, 66%; (b) 1. Et_2NH/CH_2Cl_2 1:1, rt, 1 h; 2. Fmoc-Gly-OH, EDCI, DMAP, CH_2Cl_2, rt, overnight, 47%; (c) 1. Et_2NH/CH_2Cl_2 1:1, rt, 1 h; 2. Boc-L-Cys(*t*-Bu)-OH, EDCI, DMAP, CH_2Cl_2, rt, overnight, 75%; (d) I_2, MeOH, rt, 4 h, 77%; (e) TFA/CH_2Cl_2 1:1, rt, overnight, 92%.

Compound 46 (468 mg, 66%, colorless solid) was prepared from **45** (303 mg, 1.02 mmol) and Fmoc-L-Cys(*t*-Bu)-OH (500 mg, 0.854 mmol) following the general procedures A and B. R_f (CH_2Cl_2/MeOH 25:1): 0.71; Mp: 71 – 72 °C; $[\alpha]_D^{20}$ +14.8 (*c* 1.00, $CHCl_3$); IR (neat): 3318 (w), 2924 (m), 2855 (w), 1726 (s), 1673 (m), 1493 (m), 1448 (m), 1367 (w), 1250 (m),

1149 (s), 1079 (w), 1035 (w), 845 (w), 740 (s), 700 (s), 619 (w); ^{1}H NMR (400 MHz, $CDCl_3$): 7.80 – 7.72 (m, 2H), 7.63 – 7.55 (m, 2H), 7.43 – 7.36 (m, 8H), 7.30 – 7.27 (m, 5H), 7.26 – 7.25 (m, 1H), 7.23 – 7.18 (m, 3H), 6.46 (d, $^3J_{H–H}$ = 7.7 Hz, 1H), 5.03 (d, $^3J_{H–H}$ = 8.1 Hz, 1H), 4.45 – 4.31 (m, 3H), 4.27 – 4.16 (m, 1H), 3.92 – 3.78 (m, 1H), 2.74 (dd, $^2J_{H–H}$ = 12.9 Hz, $^3J_{H–H}$ = 7.3 Hz, 1H), 2.57 (dd, $^2J_{H–H}$ = 12.9, $^3J_{H–H}$ = 5.3 Hz, 1H), 2.33 – 2.16 (m, 2H), 2.14 – 2.05 (m, 1H), 1.92 – 1.81 (m, 1H), 1.45 – 1.38 (m, 18H); ^{13}C NMR (100 MHz, $CDCl_3$): 172.1 (C), 170.3 (C), 169.7 (C), 144.3 (3xC), 143.7 (2xC), 141.3 (2xC), 129.6 (6xCH), 128.1 (6xCH), 127.7 (3xCH), 127.1 (2xCH), 126.9 (2xCH), 125.1 (2xCH), 120.0 (2xCH), 82.4 (C), 80.6 (C), 67.3 (C), 67.1 (CH_2), 54.0 (CH), 52.4 (CH), 47.1 (CH), 34.0 (CH_2), 31.4 (CH_2), 28.1 (3xCH_3), 27.9 (3xCH_3), 27.7 (CH_2); MS (MALDI-TOF): 850 ($[M+Na]^+$).

Compound 47 (1.0 g, 47%, colorless solid) was prepared from **46** (2.0 g, 2.4 mmol) and Fmoc-Gly-OH (0.9 g, 2.9 mmol) following the general procedures A and B. R_f (CH_2Cl_2/MeOH 25:1): 0.47; Mp: 110 – 111 °C; $[\alpha]_D^{20}$ +6.94 (*c* 1.00, $CHCl_3$); IR (neat): 3283 (w), 2978 (w), 1725 (w), 1646 (s), 1531 (m), 1444 (w), 1392 (w), 1368 (w), 1335 (w), 1246 (m), 1149 (s), 1096 (w), 1050 (w), 845 (w), 741 (s), 704 (m), 671 (w); ^{1}H NMR (400 MHz, $CDCl_3$): 7.79 – 7.73 (m, 2H), 7.60 – 7.55 (m, 2H), 7.46 – 7.37 (m, 8H), 7.32 – 7.26 (m, 8H), 7.23 – 7.17 (m, 3H), 6.53 (d, $^3J_{H–H}$ = 7.7 Hz, 1H), 6.15 (d, $^3J_{H–H}$ = 7.7 Hz, 1H), 5.37 (br, 1H), 4.44 – 4.33 (m, 3H), 4.24 – 4.18 (m, 1H), 4.17 – 4.09 (m, 1H), 3.83 – 3.77 (m, 2H), 2.81 (dd, $^2J_{H–H}$ = 13.1 Hz, $^3J_{H–H}$ = 6.9 Hz, 1H), 2.57 (dd, $^2J_{H–H}$ = 13.1 Hz, $^3J_{H–H}$ = 5.7 Hz, 1H), 2.33 – 2.15 (m, 2H), 2.15 – 2.00 (m, 1H), 1.97 – 1.84 (m, 1H), 1.43 (s, 9H), 1.41 (s, 9H); ^{13}C NMR (125 MHz, $CDCl_3$): 169.8 (C), 167.8 (C), 166.9 (C), 166.2 (C), 153.0 (C), 141.9 (3xC), 141.3 (2xC), 138.9 (2xC), 127.1 (6xCH), 125.7 (6xCH), 125.3 (3xCH), 124.7 (2xCH), 124.5 (2xCH), 122.7 (2xCH), 117.6 (2xCH), 80.0 (C), 78.4 (C), 64.9 (CH_2), 50.1 (CH), 49.7 (CH), 44.7 (CH), 42.0 (CH_2),

30.9 (CH_2), 29.0 (CH_2), 25.6 (3xCH_3), 25.5 (3xCH_3), 25.1 (CH_2); MS (MALDI-TOF): 907 ([M+Na]$^+$).

Compound 48 (0.9 g, 75%, colorless solid) was prepared from **47** (1.0 g, 1.1 mmol) and Boc-L-Cys(Trt)-OH (0.6 g, 1.3 mmol) following the general procedures A and B. R_f (CH_2Cl_2/MeOH 25:1): 0.34; Mp: 194 – 195 °C (decomp.); $[\alpha]_D^{20}$ +8.54 (*c* 1.00, $CHCl_3$); IR (neat): 3298 (w), 2977 (w), 1723 (m), 1644 (m), 1491 (m), 1445 (w), 1391 (w), 1367 (w), 1249 (w), 1152 (s), 1082 (w), 1033 (w), 846 (w), 743 (s), 699 (w), 619 (w); ^{1}H NMR (400 MHz, $CDCl_3$): 7.45 – 7.38 (m, 12H), 7.34 – 7.27 (m, 12H), 7.25 – 7.19 (m, 6H), 6.69 – 6.53 (m, 2H), 6.37 (br, 1H), 4.88 (br, 1H), 4.38 – 4.30 (m, 1H), 4.07 – 4.00 (m, 1H), 3.90 – 3.65 (m, 3H), 2.80 – 2.53 (m, 4H), 2.28 – 2.12 (m, 2H), 2.10 – 1.99 (m, 1H), 1.95 – 1.78 (m 1H), 1.45 – 1.38 (m, 27H); ^{13}C NMR (125 MHz, $CDCl_3$): 172.3 (C), 171.2 (C), 170.4 (C), 169.3 (C), 168.2 (C), 155.7 (C), 144.4 (3xC), 144.3 (3xC), 129.61 (6xCH), 129.55 (6xCH), 128.13 (6xCH), 128.07 (6xCH), 127.0 (3xCH), 126.9 (3xCH), 82.2 (C), 80.7 (C), 80.6 (C), 67.4 (C), 67.3 (C), 53.8 (CH), 52.4 (CH), 52.3 (CH), 43.3 (CH_2), 33.4 (CH_2), 33.2 (CH_2), 31.5 (CH_2), 28.3 (3xCH_3), 28.1 (3xCH_3), 28.0 (CH_3), 27.5 (CH_2); MS (MALDI-TOF): 1130 ([M+Na]$^+$).

Compound 49 (190 mg, 77%, colorless solid) was prepared from **48** (440 mg, 0.397 mmol) following the general procedure C. R_f (CH_2Cl_2/MeOH 25:1): 0.16; Mp: 125 – 126 °C; $[\alpha]_D^{20}$-57.1 (*c* 1.00, $CHCl_3$); IR (neat): 3313 (w), 2979 (w), 2935 (w), 1721 (m), 1655 (s), 1517 (m), 1454 (w), 1409 (w), 1367 (w), 1327 (w), 1246 (m), 1151 (s), 1048 (w), 1021 (w), 846 (w), 752 (w), 665 (w), 616 (w); ^{1}H NMR (400 MHz, $CDCl_3$): 8.05 – 7.73 (m, 2H), 7.17 (d, $^3J_{H\text{–}H}$ = 7.8 Hz, 1H), 5.68 (br, 1H), 5.25 – 4.81 (br, 1H), 4.61 – 4.39 (m, 2H), 4.33 – 3.98 (br, 1H), 3.91 – 3.71 (br, 1H), 3.62 – 3.51 (br, 1H), 3.43 – 3.25 (br, 2H), 3.11 – 2.94 (br, 1H), 2.42 – 2.23 (m, 2H), 2.17 – 2.06 (m, 1H), 2.01 – 1.88 (m, 1H), 1.46 (s, 9H), 1.45 – 1.42 (m, 18H); ^{13}C NMR (100 MHz, $CDCl_3$): 172.7 (C), 172.4 (C), 170.6 (C), 168.8 (2xC), 154.9 (C), 82.5 (C), 81.0 (C),

80.9 (C), 55.5 (CH), 52.5 (2xCH), 45.5(CH_2), 45.2 (CH_2), 31.6 (CH_2), 28.3 (3xCH_3), 28.1 (3xCH_3), 28.0 (3xCH_3), 27.7 (CH_2); MS (ESI, $CHCl_3$): 620 (100, $[M+H]^+$).

Compound 19 (83 mg, 92%, colorless solid) was prepared from **49** (190 mg, 0.306 mmol) following the general procedure E. The crude product was purified by HILIC flash chromatography (Eluent: H_2O + 0.1% TFA/CH_3CN + 0.1% TFA gradient from 5:95 to 95:5). Mp: 213 – 214 °C (decomp.); $[\alpha]_D^{20}$ -100 (*c* 0.50, DMSO); IR (neat): 3313 (w), 2970 (w), 1665 (s), 1530 (m), 1409 (w), 1201 (m), 839 (w), 800 (w), 723 (w), 665 (w); ^{1}H NMR (400 MHz, CD_3OD): 4.74 – 4.30 (m, 3H), 4.24 – 3.80 (br, 1H), 3.55 – 3.37 (m, 3H), 3.26 - 2.69 (br, 1H), 2.50 – 2.29 (m, 2H), 2.27 – 2.13 (m, 1H), 2.04 – 1.88 (m, 1H); ^{13}C NMR (125 MHz, CD_3OD): 177.2 (C), 175.3 (C), 172.8 (C), 172.0 (C), 170.2 (C), 55.5 (CH), 54.1 (CH), 53.8 (CH), 46.6 (CH_2), 43.9 (2xCH_2), 32.0 (CH_2), 28.6 (CH_2); HRMS (ESI, +ve) calcd for $C_{13}H_{20}N_4O_7S_2$ ($[M+H]^+$): 409.0847, found: 409.0846.

Note: Peaks are broad in NMR spectra due to the presence of conformers as described for similar compounds in ref. S8.

2.2.1.3. Synthesis of Cyclic Disulfides

Compound 28 was synthesized and purified according to procedures described in ref. S9.

Compound 29 was synthesized and purified according to procedures described in ref. S10.

2.2.2. Synthesis of Benzopolysulfanes (BPS)

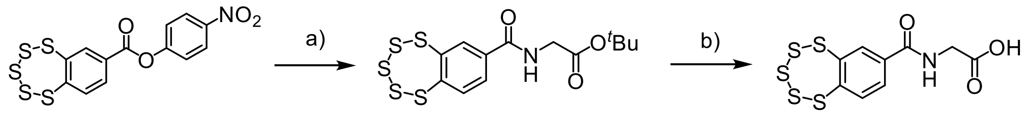

Scheme S7 (a). Glycine *tert*-butyl ester, THF, rt, overnight, 91%; (b) TFA, CH_2Cl_2, rt, overnight, 32%.

Compound 50 was synthesized and purified according to procedures described in ref. S3.

Compound 51. To a solution of **50** (30 mg, 0.075 mmol) in dry THF (3.0 mL) was added a solution of glycine *tert*-butyl ester (21 μL, 0.15 mmol) in dry THF (1.5 mL). The mixture was allowed to stir at rt under N_2 overnight. CH_2Cl_2 was added and the mixture was washed with HCl solution (1 N), saturated aqueous $NaHCO_3$ solution and brine. The organic layer was combined, dried over Na_2SO_4 and concentrated. The residue was purified by flash chromatography (SiO_2, CH_2Cl_2/MeOH 100:1, R_f 0.4) to yield **51** (27 mg, 91%) as a colorless solid. Mp: 147 – 148 °C; IR (neat): 3299 (w), 2925 (w), 2854 (w), 1736 (m), 1644 (m), 1586 (w), 1531 (m), 1448 (w), 1366 (m), 1334 (w), 1291 (w), 1224 (s), 1150 (s), 1030 (w), 1007 (w), 906 (w), 844 (m), 752 (m), 731 (w), 630 (w); ^{1}H NMR (400 MHz, $CDCl_3$): 8.25 (d, $^4J_{H-H}$ = 2.0 Hz, 1H), 7.90 (d, $^3J_{H-H}$ = 8.0 Hz, 1H), 7.73 (dd, $^3J_{H-H}$ = 8.0 Hz, $^4J_{H-H}$ = 2.0 Hz, 1H), 6.73 (t, $^3J_{H-H}$ = 4.8 Hz, 1H), 4.13 (d, $^3J_{H-H}$ = 4.8 Hz, 2H), 1.51 (s, 9 H); ^{13}C NMR (100 MHz, $CDCl_3$):

169.1 (C), 165.0 (C), 147.5 (C), 144.6 (C), 136.3 (CH), 135.6 (C), 134.4 (CH), 128.8 (CH), 83.2 (C), 42.7 (CH_2), 28.2 (CH_3); MS (ESI, $CHCl_3$): 338 (100, [M-*t*-Bu+H]$^+$).

Compound 6 (3.0 mg, 32%) was synthesized from **51** (11.0 mg, 27.9 µmol) following general procedure E in the synthesis of disulfide bridged γ-turn peptides. The crude product was purified by reversed phase flash chromatography (Eluent: H_2O + 0.1% TFA/CH_3CN + 0.1% TFA gradient from 85:15 to 20:80). Mp: 190 – 191 °C (decomp.); IR (neat): 3306 (br), 2920 (br), 1719 (m), 1634 (s), 1585 (m), 1536 (s), 1406 (m), 1310 (m), 1202 (s), 1007 (w), 902 (w), 843 (w), 755 (w), 679 (w); ^{1}H NMR (500 MHz, CD_3OD): 8.38 (d, $^4J_{H-H}$ = 2.0 Hz, 1H), 8.02 (d, $^3J_{H-H}$ = 8.0 Hz, 1H), 7.89 (dd, $^3J_{H-H}$ = 8.0, $^4J_{H-H}$ =2.0 Hz, 1H), 4.11 (s, 2H); ^{13}C NMR (125 MHz, CD_3OD): 171.7 (C), 166.6 (C), 147.1 (2xC), 144.2 (C), 135.9 (CH), 134.4 (CH), 129.0 (CH), 41.1 (CH_2); MS (ESI, $CHCl_3$): 338 (100, [M+H]$^+$).

Compound 10 was synthesized and purified according to procedures described in ref. S3.

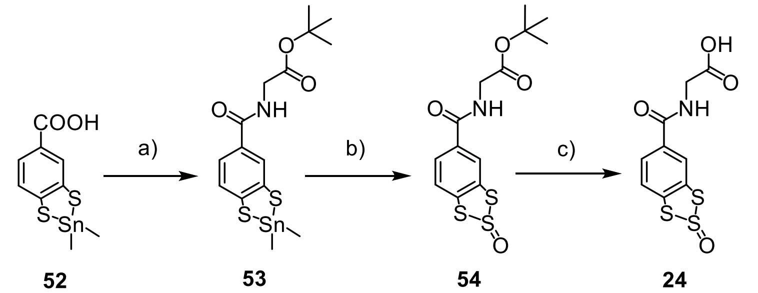

Scheme S8 (a) Glycine *tert*-butyl ester, EDCI, CH_2Cl_2, rt, overnight, 67%; (b) $SOCl_2$, dry THF, -60 °C to -10 °C, 23%; (c) TFA/CH_2Cl_2 (1:1), 45%.

Compound 52 was synthesized and purified according to procedures described in ref. S3.

Compound 53. To a solution of **52** (50.0 mg, 0.150 mmol) in CH_2Cl_2 (1.5 mL), glycine *tert*-butyl ester (24.0 mg, 0.183 mmol) was added followed by the addition of EDCI (36.0 mg, 0.183 mmol). The reaction mixture was stirred at rt overnight, then diluted with CH_2Cl_2. The reaction mixture was washed with 10% aqueous solution of citric acid, water, saturated aqueous solution of $NaHCO_3$ and brine. The organic layer was dried over anhydrous Na_2SO_4. The solvent was removed under vacuum. The residue (45.0 mg, 67%) was used for the next step without further purification.

Compound 54. To a solution of **53** (45.0 mg, 0.100 mmol) in dry THF (774 µL) at -60 °C, fresh $SOCl_2$ (10.0 µL, 0.140 mmol) was added. The reaction mixture was slowly warmed up to -10 °C in 4 h. Then bi-distilled water (387 µL) was added to the reaction mixture to quench the excess of $SOCl_2$. The reaction mixture was extracted using CH_2Cl_2. The organic layer was dried over anhydrous Na_2SO_4, filtered and concentrated under vacuum. The residue was purified by reversed phase flash chromatography (Eluent: H_2O + 0.1% TFA/CH_3CN + 0.1% TFA gradient from 90:10 to 20:80) to yield the product as a colorless solid (8.0 mg, 23%). Mp: 76 – 77 °C; IR (neat): 3321 (w), 2981 (w), 2937 (w), 1739 (m), 1642 (m), 1582 (w), 1530 (m), 1454 (w), 1403 (w), 1394 (w), 1367 (m), 1315 (w), 1227 (s), 1152 (s), 1115 (s), 1032 (w), 1009 (w), 944 (w), 896 (w), 842 (w), 752 (m), 699 (w); ^{1}H NMR (400 MHz, $CDCl_3$): 8.09 (dd, $^4J_{H–H}$ = 1.7, $^5J_{H–H}$ = 0.5 Hz, 1H), 7.77 (dd, $^3J_{H–H}$ = 8.3, $^4J_{H–H}$ = 1.7 Hz, 1H), 7.67 (dd, $^3J_{H–H}$ = 8.3, $^5J_{H–H}$ = 0.5 Hz, 1H), 6.66 (br, 1H), 4.15 (d, $^3J_{H–H}$ = 4.8 Hz, 2H), 1.52 (s, 9H); ^{13}C NMR (100 MHz, $CDCl_3$): 169.0 (C), 165.5 (C), 139.6 (C), 137.0 (C), 133.4 (C), 125.9 (CH), 124.6 (CH), 123.7 (CH), 83.0 (C), 42.7 (CH_2), 28.1 (3xCH_3); MS (ESI, $CHCl_3$): 691 (55, $[2M+H]^+$), 346 (28, $[M+H]^+$), 290 (100, [M-*t*-Bu+H]$^+$).

Compound 24 (3.0 mg, 45%, colorless solid) was synthesized from **54** (8.0 mg, 23 µmol) following general procedure E in the synthesis of disulfide bridged γ-turn peptides. The crude product was purified by reversed phase flash chromatography (Eluent: H_2O + 0.1% TFA/CH_3CN + 0.1% TFA gradient from 95:5 to 30:70). Mp: 112 – 113 °C (decomp.); IR (neat): 3319 (br), 3076 (br), 1723 (m), 1638 (m), 1583 (m), 1531 (m), 1453 (w), 1406 (w), 1312 (w), 1199 (m), 1111 (s), 1030 (w), 1007 (w), 890 (w), 830 (w), 752 (w), 681 (w), 625 (w); ^{1}H NMR (400 MHz, CD_3OD): 8.90 (br, 1H, exchange with CD_3OD), 8.17 (d, $^4J_{H-H}$ = 1.7 Hz, 1H), 7.88 (dd, $^3J_{H-H}$ = 8.3, $^4J_{H-H}$ = 1.7 Hz, 1H), 7.80 (d, $^3J_{H-H}$ = 8.3 Hz, 1H), 4.10 (s, 2H); ^{13}C NMR (125 MHz, CD_3OD): 171.6 (C), 167.3 (C), 139.8 (C), 136.8 (C), 133.3 (C), 126.0 (CH), 124.2 (CH), 123.3 (CH), 40.9 (CH_2); MS (ESI, $CHCl_3$): 290 (100, $[M+H]^+$).

2.2.3. Synthesis of Heteroaromatic Sulfones

Compound 11 was synthesized and purified according to procedures described in ref. S11.

Compound 14 was synthesized and purified according to procedures described in ref. S11.

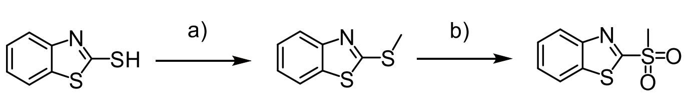

55 56 21

Scheme S9 (a) CH_3I, TEA, CH_2Cl_2, rt, 10 min, 83%; (b) *m*-CPBA, CH_2Cl_2, rt, 2 h, 82%.

Compound 56. To a suspension of **55** (2.0 g, 12 mmol) in CH_2Cl_2 (12.0 mL), a solution of CH_3I (1.1 mL, 18 mmol) in CH_2Cl_2 (4.0 mL) was added, followed by the addition of a solution of TEA (2.5 mL, 18 mmol) in CH_2Cl_2 (4.0 mL). After being clear, the reaction mixture was kept stirring at rt for 10 min. Then the reaction mixture was diluted with CH_2Cl_2, washed with water, brine, and dried over anhydrous Na_2SO_4. The solvent was removed *in vacuo*. The residue was purified by flash chromatography (SiO_2, CH_2Cl_2/pentane 1:1) to yield the desired product as a colorless solid (1.8 g, 83%). R_f (CH_2Cl_2/pentane 1:1): 0.33. Spectroscopic data were consistent with those reported in ref. S12.

Compound 21. To a solution of **56** (0.8 g, 4.4 mmol) in CH_2Cl_2 (20 mL), *m*-CPBA (2.1 g, 9.2 mmol) was added. The reaction mixture was stirred at rt for 2 h. The reaction mixture was diluted with CH_2Cl_2 and washed with brine 3 times. The organic layer was dried over anhydrous Na_2SO_4. The solvent was removed *in vacuo*. The residue was purified by flash chromatography (SiO_2, CH_2Cl_2) to yield the desired product as a colorless solid (0.8 g, 82%). R_f (CH_2Cl_2): 0.41. Spectroscopic data were consistent with those reported in ref. S13.

2.2.4. Synthesis of Thiosulfinates

Compound 17 and **22** was synthesized and purified according to procedures described in ref. S14.

Compound 23 was synthesized and purified according to procedures described in ref. S15.

Compound 27 was synthesized and purified according to procedures described in ref. S16.

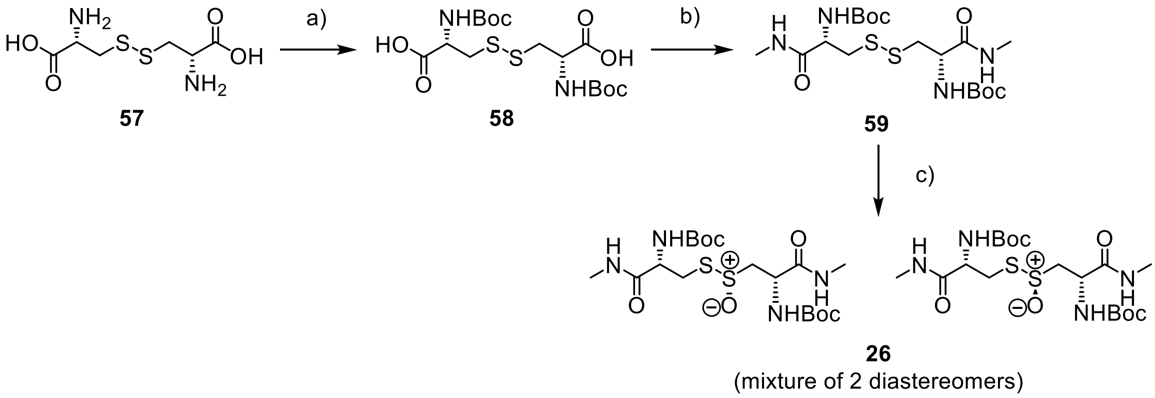

Scheme S10 (a) Boc_2O, TEA, H_2O, rt, 4 h, 94%; (b) Isobutyl chloroformate, CH_3NH_2 (2 M solution in THF), THF, 0 °C to rt, 24 h, 48%; (c) *m*-CPBA, CH_2Cl_2, rt, 0 °C, 1 h, 23%.

Compound 58 was synthesized and purified according to procedures described in ref. S17.

Compound 59 was synthesized and purified according to procedures described in ref. S18.

Compound 26. To a suspension of **59** (250 mg, 0.536 mmol) in CH_2Cl_2 (11.0 mL) at 0 °C, was added *m*-CPBA (132 mg, 0.574 mmol). The reaction mixture was stirred at 0 °C for 1 h, then warmed up to rt. The reaction mixture was diluted with CH_2Cl_2, and washed with saturated aqueous solution of $NaHCO_3$ 3 times. The organic layer was dried over anhydrous Na_2SO_4, filtered and concentrated under vacuum. The residue was purified by flash chromatography (SiO_2, CH_2Cl_2/MeOH 15:1) to yield the desired product as a colorless solid (60 mg, 23%, mixture of 2 diastereomers). R_f (CH_2Cl_2/MeOH 15:1): 0.19; IR (neat): 3324 (m), 2977 (w), 1687 (m), 1652 (s), 1521 (s), 1412 (w), 1366 (m), 1320 (m), 1250 (m), 1163 (s), 1065 (m), 1047 (m), 1023 (m), 870 (w), 779 (w), 648 (w); ^1H NMR (400 MHz, $CDCl_3$): 7.53 – 6.90 (m, 2H), 6.08 – 5.64 (m, 2H), 4.81 – 4.38 (m, 2H), 3.87 – 3.31 (m, 4H), 2.82 – 2.74 (m, 6H),

1.46 – 1.33 (m, 18H); ^{13}C NMR (100 MHz, $CDCl_3$, nn/nn-diastereomeric peaks):170.9 (C), 169.7/169.1 (C), 156.1 (C), 155.4 (C), 81.0/80.8 (C), 80.3 (C), 65.4/41.8 (CH_2), 54.8 (CH), 54.4/49.8 (CH), 47.2 (CH_2), 28.4 ($3xCH_3$), 28.3 ($3xCH_3$), 26.3/26.2 (CH_3), 26.0 (CH_3); MS (ESI, MeOH): 483 (100, $[M+H]^+$).

2.2.5. Synthesis of Thiosulfonates

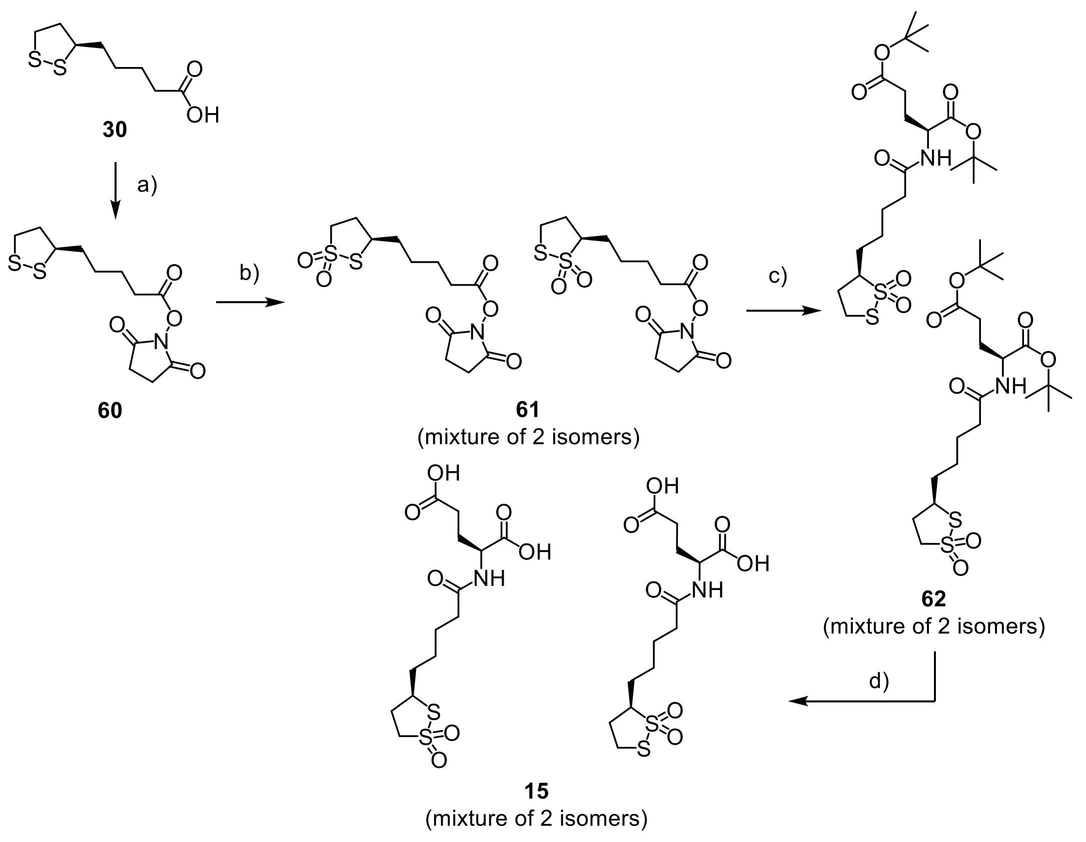

Scheme S11 (a) NHS, EDCI, CH_2Cl_2, rt, overnight, 68%; (b) *m*-CPBA, rt, 24 h, 48%; (c) L-glutamic acid di-*tert*-butyl ester hydrochloride, TEA, CH_2Cl_2, rt, overnight, 51%; (d) TFA/CH_2Cl_2 1:1, rt, 24 h, 100%.

Compound 60. To a solution of **30** (1.0 g, 4.8 mmol) in CH_2Cl_2 (30 mL), NHS (0.6 g, 4.8 mmol) was added, followed by EDCI (1.1 g, 5.3 mmol). The reaction mixture was stirred at rt overnight. Then the mixture was diluted with CH_2Cl_2, washed with water 3 times. The organic layer was dried over anhydrous Na_2SO_4 and concentrated *in vacuo*. The residue was purified by flash chromatography (SiO_2, CH_2Cl_2) to yield the desired product as a yellow solid (1.3 g, 68%). R_f (CH_2Cl_2): 0.23. Spectroscopic data were consistent with those reported in ref. S19.

Compound 61. To a solution of **60** (68 mg, 0.22 mmol) in CH_2Cl_2 (2.0 mL), was added *m*-CPBA (128 mg, 0.556 mmol). The resulting mixture was stirred at rt for 24 h. The reaction mixture was diluted with CH_2Cl_2, washed with saturated aqueous solution of $NaHCO_3$ 3 times. The organic layer was dried over anhydrous Na_2SO_4. The solvent was removed under vacuum. The residue was purified by flash chromatography (SiO_2, CH_2Cl_2/MeOH 18:1) to yield the desired product as a colorless oil (36 mg, 48%, mixture of 2 isomers). R_f (CH_2Cl_2/MeOH 15:1): 0.47; IR (neat): 2941 (w), 1812 (w), 1782 (w), 1734 (s), 1430 (w), 1362 (w), 1302 (m), 1206 (m), 1127 (m), 1068 (m), 995 (w), 814 (w), 708 (w), 650 (w); ^{1}H NMR (500 MHz, $CDCl_3$, nn/nn-55:45 isomeric peaks, some isomeric peaks overlap): 4.23 – 4.16/3.39 – 3.31 (m, 1H), 3.66 – 3.41 (m, 2H), 2.83 (s, 4H), 2.73 – 2.57 (m, 3H), 2.36 – 2.18 (m, 1H), 1.74 – 1.69/2.10 – 1.99 (m, 1H), 1.95 – 1.86 (m, 1H), 1.84 – 1.76 (m, 2H), 1.66 – 1.45 (m, 2H); ^{13}C NMR (100 MHz, $CDCl_3$, nn/nn isomeric peaks, some isomeric peaks could not be assigned due to overlap in ^{1}H NMR): 169.2/168.3 (C), 68.6/55.0 (CH), 59.6/35.9 (CH_2), 33.27 (CH_2), 31.2 (CH_2), 30.63 (CH_2), 30.57 (CH_2), 30.2 (CH_2), 27.3 (CH_2), 27.0 (CH_2), 25.9 (CH_2), 25.6 (CH_2), 24.3/24.1 (CH_2); MS (ESI, MeOH): 336 (100, $[M+H]^+$).

Compound 62. To a solution of **61** (36 mg, 0.11 mmol) in CH_2Cl_2 (150 μL), was added glutamic acid di-*tert*-butyl ester hydrochloride (26 mg, 0.13 mmol), followed by TEA (21 μL,

0.17 mmol). The reaction mixture was kept stirred at rt overnight. The reaction mixture was diluted with CH_2Cl_2, washed with 10% aqueous solution of citric acid, water, saturated solution of $NaHCO_3$ and brine. The organic layer was dried over anhydrous Na_2SO_4. Then the solvent was removed *in vacuo*. The residue was purified by flash chromatography (SiO_2, CH_2Cl_2/MeOH 25:1) to yield the desired product as a colorless oil (26 mg, 51%, mixture of 2 isomers). R_f (CH_2Cl_2/MeOH 25:1): 0.49; IR (neat): 3307 (w), 2977 (w), 2929 (w), 1728 (m), 1652 (m), 1539 (w), 1455 (w), 1368 (w), 1305 (m), 1255 (m), 1152 (s), 1130 (s), 965 (w), 846 (w), 752 (w), 709 (w), 673 (w); ^{1}H NMR (400 MHz, $CDCl_3$, nn/nn-55:45 isomeric peaks, some isomeric peaks overlap): 6.21 (d, $^3J_{H–H}$ = 8.1 Hz, 1H), 4.50 – 4.43 (m, 1H), 4.25 – 4.15/3.41 – 3.32 (m, 1H), 3.67 – 3.42 (m, 2H), 2.74 – 2.63 (m, 1H), 2.40 – 2.18 (m, 5H), 2.16 – 2.01 (m, 2H), 1.98 – 1.83 (m, 2H), 1.79 – 1.64 (m, 3H), 1.63 – 1.52 (m, 1H), 1.48 (s, 9H), 1.45 (s, 9H); ^{13}C NMR (100 MHz, $CDCl_3$, nn/nn isomeric peaks, some isomeric peaks could not be assigned due to overlap in ^{1}H NMR): 172.37/172.35 (C), 172.22/172.18 (C), 171.24/171.21 (C), 82.4/82.3 (C), 80.84/80.82 (C), 68.7/55.2 (CH), 59.6/36.2 (CH_2), 52.3 (CH), 35.9/35.8 (CH_2), 33.2 (CH_2), 31.6 (CH_2), 31.3 (CH_2), 30.2 (CH_2), 28.1 (6xCH_3), 28.0 (6xCH_3), 27.6 (CH_2), 27.5 (CH_2), 27.5 (CH_2), 26.4 (CH_2), 25.0/24.9 (CH_2); MS (ESI, MeOH): 480 (100, $[M+H]^+$).

Compound 15 (20 mg, 100%, colorless oil, mixture of 2 isomers) was prepared from **62** (26 mg, 0.05 mmol) following the general procedure E described in the synthesis of disulfide bridged γ-turn peptides. The crude product was purified by reversed phase flash chromatography (Eluent: H_2O + 0.1% TFA/CH_3CN + 0.1% TFA gradient from 95:5 to 40:60). IR (neat): 3340 (w), 2936 (w), 2507 (w), 1719 (s), 1628 (m), 1548 (w), 1444 (w), 1413 (w), 1296 (m), 1208 (m), 1174 (m), 1124 (s), 1025 (w), 784 (w), 709 (w), 672 (w); ^{1}H NMR (400 MHz, CD_3OD, nn/nn-55:45 isomeric peaks, some isomeric peaks overlap): 4.45 – 4.37 (m, 1H), 4.34 – 4.24/3.48 – 3.41 (m, 1H), 3.72 – 3.50 (m, 2H), 2.78 – 2.64 (m, 1H), 2.44 – 2.34 (m, 2H),

2.32 – 2.13 (m, 4H), 2.01 – 1.75 (m, 3H), 1.71 – 1.40 (m, 4H); ^{13}C NMR (100 MHz, CD_3OD, nn/nn isomeric peaks, some isomeric peaks could not be assigned due to overlap in ^{1}H NMR): 178.8 (C), 178.6 (C), 177.49/177.47 (C), 72.6/59.5 (CH), 63.3/39.8 (CH_2), 55.48/55.46 (CH), 38.9/38.8 (CH_2), 36.9 (CH_2), 35.1 (CH_2), 34.2 (CH_2), 33.8 (CH_2), 31.03/31.02 (CH_2), 30.33/30.31 (CH_2), 29.9 (CH_2), 29.0/28.9 (CH_2); MS (ESI, MeOH): 368 (100, $[M+H]^+$).

Compound 16 was synthesized and purified according to procedures described in ref. S20.

2.2.6. Synthesis of Inorganic Polysulfides (IPS)

Following a reported procedure with modifications,[S21] elemental sulfur (30 mg, 0.12 mmol) was added to a solution of sodium sulfide (32 mg, 0.4 mmol) in water (2.0 mL) and the reaction mixture was stirred for 30 min at 70 °C under inert atmosphere. The corresponding orange solution (0.2 M, Na_2S_n, n = 2–8)[S22] was cooled down to rt and directly used as stock solution of inorganic polysulfides **13**.

3. Cell Culture

Human cervical cancer-derived HeLa Kyoto cells were cultured in DMEM (GlutaMAX, 4.5 g/L D-glucose, with phenol red) medium containing 10% fetal bovine serum (FBS) and 1% Penicillin/Streptomycin (PS). The cells were grown at 37 °C under 5% CO_2 on a 25 cm^3 tissue culture flask (TPD Corporation). Cells were detached by treatment with 1.5 mL of TrypLE Express at 37 °C for 5 min, followed by the addition of 6 mL of DMEM (GlutaMAX, 4.5 g/L D-glucose, with phenol red) medium at 37 °C. The cells were resuspended in DMEM (GlutaMAX, 4.5 g/L D-glucose, with phenol red) medium and plated according to the concentration needed.

4. High-Content High-Throughput (HCHT) Inhibitor Screening

4.1. General Procedure for HCHT Inhibitor Screening

4.1.1. Pre-Incubation of Inhibitors

HeLa Kyoto cells were seeded at 1.2 ×10^{4} cells/well in FluoroBrite DMEM + 10% FBS on µ-Plate 96-well Black ibiTreat sterile and kept at 37 °C with 5% CO_2 overnight. Next day, serial dilutions of the inhibitors in PBS (10x final concentration), reporter **1** or **2** (100 µM in PBS) and a solution of Hoechst 33342 (100 µg/mL) and PI (10 µg/mL) in PBS were prepared freshly in a 96-well V-bottom plate. Then, cells were washed with PBS (3 x 3 mL/well) and the media was exchanged to FluoroBrite DMEM (4 x 150 µL/well) using a plate washer (Biotek EL406®), keeping always a final volume of 135 µL/well. The inhibitor solutions from the V-bottom plate were added to the cells (15 µL/well, 10x final concentration in PBS) using an electronic multichannel pipettes to reach a final volume of 150 µL/well. Cells were incubated for the time of interest (for details of each inhibitors, see **Section 4.3 and 4.4**) at 37 °C with 5% CO_2. After this, cells were washed again using the plate washer and reporter solution **1** or **2** from the V-bottom plate was added (15 µL/well) using an electronic multichannel pipettes to reach a final volume of 150 µL/well and a final concentration of 10 µM, except for the control wells, where only PBS was added (15 µL/well). After 30 min of incubation at 37 °C with 5% CO_2, the plate was washed again using the plate washer. Then, the solution of Hoechst 33342 and PI from V-bottom plate was added (15 µL/well) using an electronic multichannel pipettes to reach a final volume of 150 µL/well. After 15 min of incubation at 37 °C with 5% CO_2, the plate was washed one last time and the cells were kept in clean FluoroBrite DMEM. During imaging, samples were kept at 37 °C with 5% CO_2 in the microscope.

4.1.2. Co-Incubation of Inhibitors and Reporters

HeLa Kyoto cells were seeded at 1.2 $\times 10^4$ cells/well in FluoroBrite DMEM + 10% FBS on µ-Plate 96-well Black ibiTreat sterile and kept at 37 °C with 5% CO_2 overnight. Next day, serial dilutions of the inhibitors in PBS (10x final concentration), reporter **1** or **2** (110 µM in PBS) and a solution of Hoechst 33342 (100 µg/mL) and PI (10 µg/mL) in PBS were prepared freshly in a 96-well V-bottom plate. Then, cells were washed with the plate washer. The inhibitor solutions from the V-bottom plate were added to the cells (15 µL/well, 10x final concentration in PBS) using an electronic multichannel pipettes to reach a final volume of 150 µL/well. Cells were incubated for the time of interest (for details of each inhibitors, see **Section 4.3 and 4.4**) at 37 °C with 5% CO_2. Reporter solution **1** or **2** from V-bottom plate was then added (15 µL/well) using an electronic multichannel pipettes to reach a final volume of 165 µL/well and a final concentration of 10 µM 30 min before the next washing process, except for the control wells, where only PBS was added (15 µL/well). The plate was washed again using the plate washer. The solution of Hoechst 33342 and PI from V-bottom plate was added (15 µL/well) using an electronic multichannel pipettes to reach a final volume of 150 µL/well. After 15 min of incubation at 37 °C with 5% CO_2, the plate was washed one last time and the cells were kept in clean FluoroBrite DMEM. During imaging, samples were kept at 37 °C with 5% CO_2 in the microscope.

Note: For each of both pre-incubation and co-incubation experiment, 4 images at 10x were recorded per well using three channels: blue for Hoechst 33342 (excitation filter: 377/50 nm; emission filter: 477/60 nm), green for FITC reporters (excitation filter: 475/34 nm; emission filter: 536/40 nm) and red for PI (excitation filter: 531/40 nm; emission filter: 593/40 nm), as shown in Figure S2. Dulipicates were performed for each condition.

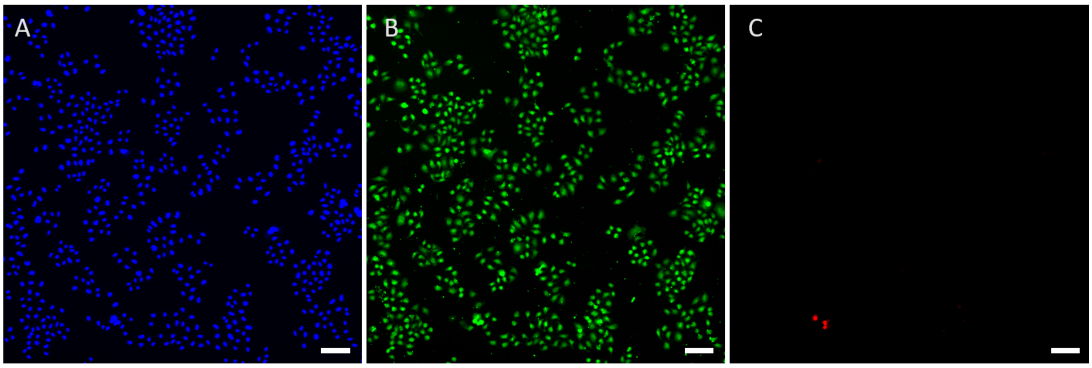

Fig. S2 (A) Blue channel recording the Hoechst 33342 localization in the nuclei. (B) Green channel recording the reporter signal in the cells. (C) Red channel recording the PI signal in the cells. Scale bar: 50 μm.

4.2. Data Analysis for HCHT Inhibitor Screening

To first mask each cell, blue channel (Hoechst 33342) was used to detect the nucleus and the cell body (thanks to background signal in the cytoplasm) (Figure S3).

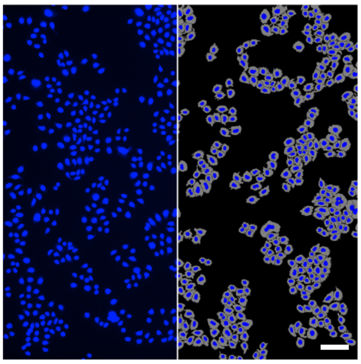

Fig. S3 Segmentation of nucleus and cell body (right) using the blue channels (left). The two parts of the cells are later on associated to create a "cell" mask. Scale bar: 50 μm.

To be able to extract the fluorescent integrated intensity information (sum of the intensities of all the pixels included in the mask) from the green channel for live cell only, the image analysis pipeline contains several steps of cell filtering and selection:

1) Firstly, PI is used to stain any remaining nuclei of dead cell. In the analysis pipeline, all the "dead" nuclei are detected based on their fluorescent intensity in the PI channel (Figure S4).

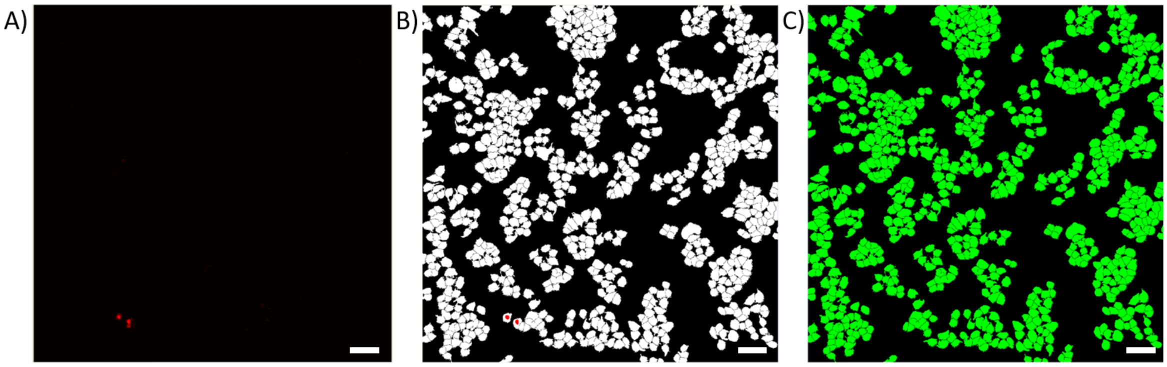

Fig. S4 (A) PI positive nuclei mask (fluorescent intensity threshold of 1000). (B) Overlap image of dead nuclei mask and original all cell body mask. (C) Resulting cell mask after removing any cell marked by a dead nucleus, new mask is called "Live Cell". Scale bar: 50 µm.

2) Elimination of non-full cells from the border of the image is performed, to avoid inaccurate fluorescent integrated intensity quantification (fluorescent integrated intensity = sum of intensity of all the pixel of the object) (Figure S5).

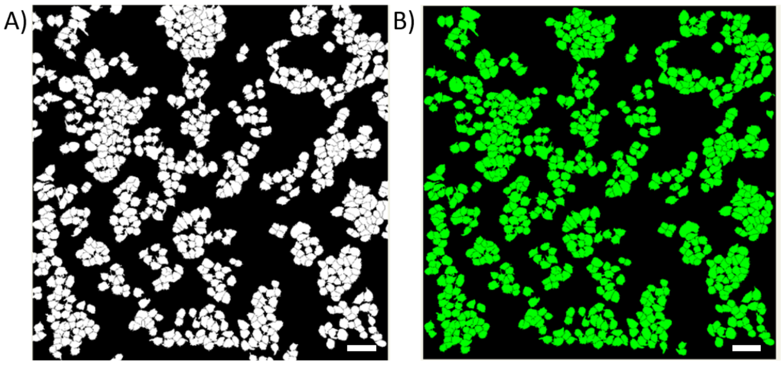

Fig. S5 (A) All live cell mask before to remove non-full border cell. (B) Resulting mask including only full cell. Scale bar: 50 µm.

3) Finally, the analysis pipeline includes a detection of fluorophore aggregates which could highly affect the quantification. The very bright aggregates are detected based on their size and brightness. The resulting object is then slightly grown (5 pixels) and used to remove all cells in the vicinity. The aggregate object is even removed from the master image object which ensure that no fluorescent intensity data can be retrieved from those area (Figure S6).

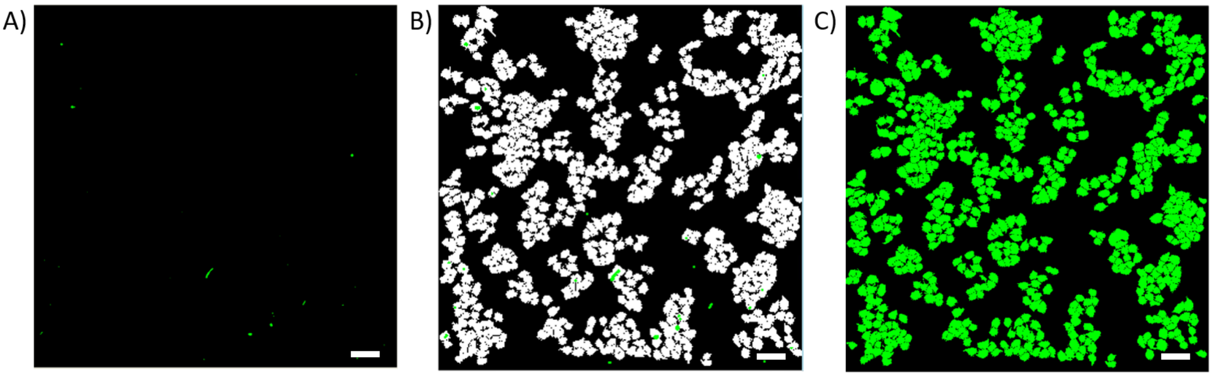

Fig. S6 (A) Bright fluorophore aggregates from green channel. (B) Overlap image of fluorophore aggregates and cell mask including only full live cell. (C) Final live cell mask. Scale bar: 50 µm.

4.3. HCHT Inhibitor Screening with ETP as Reporter

Integrated fluorescent intensity values (per cell) for each condition in the presence of inhibitors were normalized using the value of integrated fluorescent intensity (per cell) with addition of reporter **1**, Hoechst 33342 and PI as maximum signal ($I_{rel} = 1$) and the value of integrated fluorescent intensity (per cell) with only addition of Hoechst 33342 and PI as minimum ($I_{rel} = 0$), for each set of experiments. Duplicates were performed for each condition. The resulting dependence of the relative fluorescent intensity values (I_{rel}) to the concentration of inhibitors ($c_{inhibitor}$) was plotted and fitted with Equation (S1) to retrieve the half maximal inhibitory concentration (IC_{50}) value and the Hill coefficient (n).

$$I_{rel} = 1 / (1 + (IC_{50} / c_{inhibitor})^n) \qquad (S1)$$

Relative cell viability (RV) for each condition in the presence of inhibitors were calculated using the count value of living cells by dividing the count value of living cells with addition of reporter **1**, Hoechst 33342 and PI, for each set of experiments. Duplicates were performed for each condition. The resulting dependence of the relative cell viability (RV_{rel}) to the concentration of inhibitors ($c_{inhibitor}$) was plotted and fitted with Equation (S2) to retrieve the concentration causing 50% cell growth inhibition (GI_{50}) value and the Hill coefficient (n).

$$RV_{rel} = 1 / (1 + (GI_{50} / c_{inhibitor})^n) \qquad (S2)$$

Note: Reporter **1** were not toxic for 1 h at 10 μM.[S2] Also in this study, the count value of living cells with addition of reporter **1**, Hoechst 33342 and PI is always similar to that of living cells with only addition of Hoechst 33342 and PI.

4.3.1. Screening with Disulfides as Inhibitors

4.3.1.1. Screening with ETP as Inhibitors

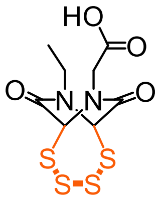

3

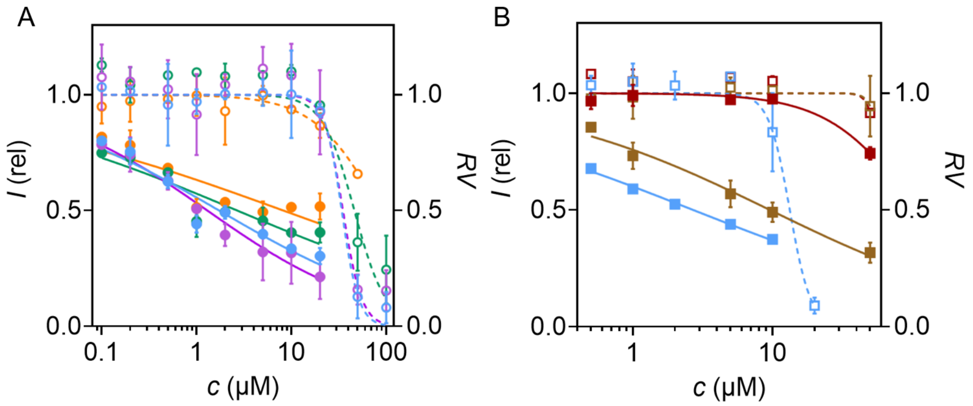

Fig. S7 Automatically analyzed HCHT data showing fluorescence intensity (filled symbols) and relative viability (empty symbols) of HeLa cells after A) pre-incubation with **3** for 30 min (blue circles), 1 h (purple circles), 2 h (green circles) and 4 h (yellow circles) followed by incubation with **1** (10 µM) for 30 min; B) incubation with **3** for 30 min (blue squares), 5.5 h with FBS (brown squares) and 23.5 h with FBS (red squares) followed by co-incubation with **1** (10 µM) for 30 min.

Table S1 Inhibition of cellular uptake of **1** by **3** and cytotoxicity of **3** as a function of assay conditions

Entry	t_{pre}[a] (h)	t_{inc}[b] (h)	t_{co}[c] (h)	MIC[d] (μM)	IC_{50}[e] (μM)	*n* (IC_{50})[f]	GI_{50}[g] (μM)	*n* (GI_{50})[h]
1	0.5	0.5	-	< 0.1	1.7 ± 0.2	-0.41 ± 0.04	34.0 ± 2.9	-4.8 ± 1.0
2	1	0.5	-	< 0.1	1.3 ± 0.2	-0.50 ± 0.05	35.0 ± 4.4	-4.3 ± 1.3
3	2	0.5	-	< 0.1	2.7 ± 0.6	-0.30 ± 0.04	46.1 ± 4.8	-2.6 ± 0.6
4	4	0.5	-	< 0.1	8.2 ± 3.2	-0.26 ± 0.04	> 50	-
5	0.5	-	0.5	< 0.5	2.7 ± 0.1	-0.41 ± 0.01	13.3 ± 0.8	-5.8 ± 1.1
6	5.5[i]	-	0.5	< 0.5	9.6 ± 1.2	-0.50 ± 0.04	> 50	-
7	23.5[i]	-	0.5	30	> 50	-	> 50	-

[a]Pre-incubation time of HeLa cells with **3**. [b]Incubation time with **1** after pre-incubation[a] and removal of **3**. [c]Co-incubation time with **3** and **1** after pre-incubation[a] with **3**. [d]Minimum inhibitory concentration of **3**. [e]Half maximal inhibitory concentration of **3**. [f]Hill coefficient for inhibition of cellular uptake. [g]Concentration of **3** causing 50% cell growth inhibition. [h]Hill coefficient for toxicity. [i]Incubation with FBS.

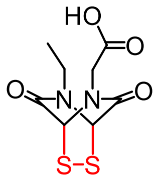

4

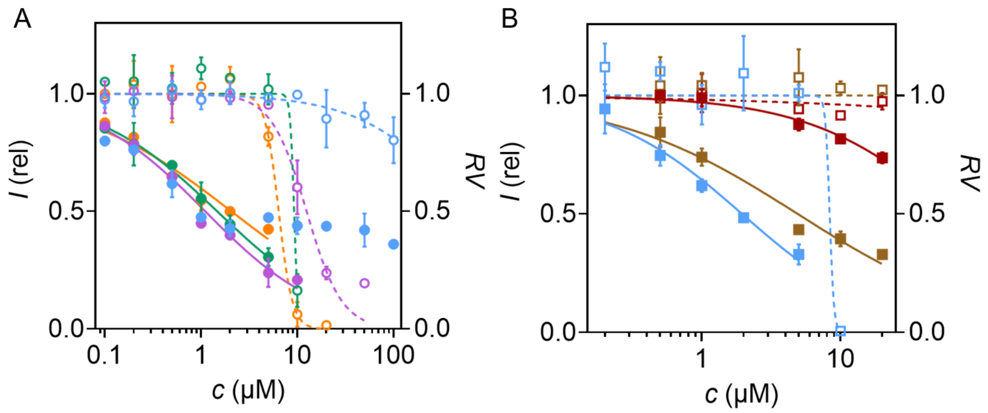

Fig. S8 Automatically analyzed HCHT data showing fluorescence intensity (filled symbols) and relative viability (empty symbols) of HeLa cells after A) pre-incubation with **4** for 30 min (blue circles), 1 h (purple circles), 2 h (green circles) and 4 h (yellow circles) followed by incubation with **1** (10 µM) for 30 min; B) incubation with **4** for 30 min (blue squares), 5.5 h with FBS (brown squares) and 23.5 h with FBS (red squares) followed by co-incubation with **1** (10 µM) for 30 min.

Table S2 Inhibition of cellular uptake of **1** by **4** and cytotoxicity of **4** as a function of assay conditions

Entry	t_{pre}[a] (h)	t_{inc}[b] (h)	t_{co}[c] (h)	MIC[d] (μM)	IC_{50}[e] (μM)	n (IC_{50})[f]	GI_{50}[g] (μM)	n (GI_{50})[h]
1	0.5	0.5	-	< 0.1	4.4 ± 1.3	-0.23 ± 0.03	> 100	-
2	1	0.5	-	0.1	1.1 ± 0.1	-0.71 ± 0.04	12.5 ± 0.7	-2.4 ± 0.3
3	2	0.5	-	0.1	1.5 ± 0.1	-0.67 ± 0.05	~ 9.2	-
4	4	0.5	-	0.1	2.1 ± 0.2	-0.55 ± 0.05	6.4 ± 0.3	-6.1 ± 1.1
5	0.5	-	0.5	0.3	2.0 ± 0.2	-0.88 ± 0.09	~ 8.4	-
6	5.5[i]	-	0.5	0.3	4.9 ± 0.5	-0.64 ± 0.06	> 20	-
7	23.5[i]	-	0.5	8.1	66 ± 14	-0.83 ± 0.10	> 20	-

[a]Pre-incubation time of HeLa cells with **4**. [b]Incubation time with **1** after pre-incubation[a] and removal of **4**. [c]Co-incubation time with **4** and **1** after pre-incubation[a] with **4**. [d]Minimum inhibitory concentration of **4**. [e]Half maximal inhibitory concentration of **4**. [f]Hill coefficient for inhibition of cellular uptake. [g]Concentration of **4** causing 50% cell growth inhibition. [h]Hill coefficient for toxicity. [i]Incubation with FBS.

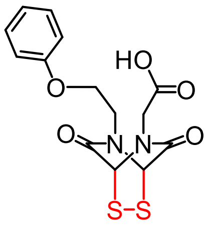

5

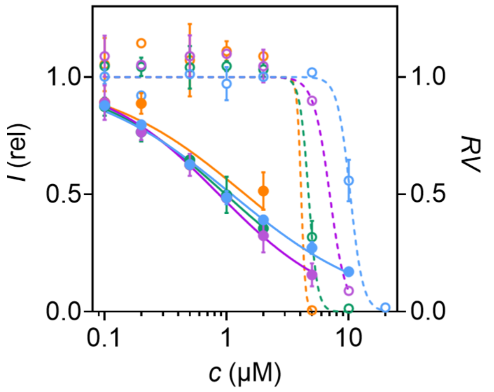

Fig. S9 Automatically analyzed HCHT data showing fluorescence intensity (filled symbols) and relative viability (empty symbols) of HeLa cells after pre-incubation with **5** for 30 min (blue circles), 1 h (purple circles), 2 h (green circles) and 4 h (yellow circles) followed by incubation with **1** (10 μM) for 30 min.

Table S3 Inhibition of cellular uptake of **1** by **5** and cytotoxicity of **5** as a function of assay conditions

Entry	t_{pre}[a] (h)	t_{inc}[b] (h)	MIC[c] (μM)	IC_{50}[d] (μM)	n (IC_{50})[e]	GI_{50}[f] (μM)	n (GI_{50})[g]
1	0.5	0.5	0.1	1.10 ± 0.05	-0.74 ± 0.03	10.3 ± 0.4	-8.2 ± 8.9
2	1	0.5	0.1	0.86 ± 0.06	-0.90 ± 0.06	7.0 ± 0.6	-6.5 ± 1.6
3	2	0.5	0.1	0.98 ± 0.07	-0.81 ± 0.06	~ 4.7	-
4	4	0.5	0.1	1.42 ± 0.26	-0.74 ± 0.13	~ 4.1	-

[a]Pre-incubation time of HeLa cells with **5**. [b]Incubation time with **1** after pre-incubation[a] and removal of **5**. [c]Minimum inhibitory concentration of **5**. [d]Half maximal inhibitory concentration

of **5**. [e]Hill coefficient for inhibition of cellular uptake. [f]Concentration of **5** causing 50% cell growth inhibition. [g]Hill coefficient for toxicity.

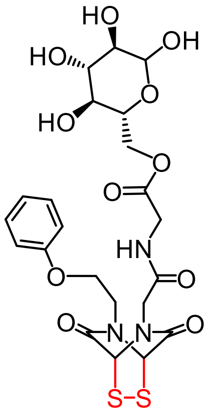

7

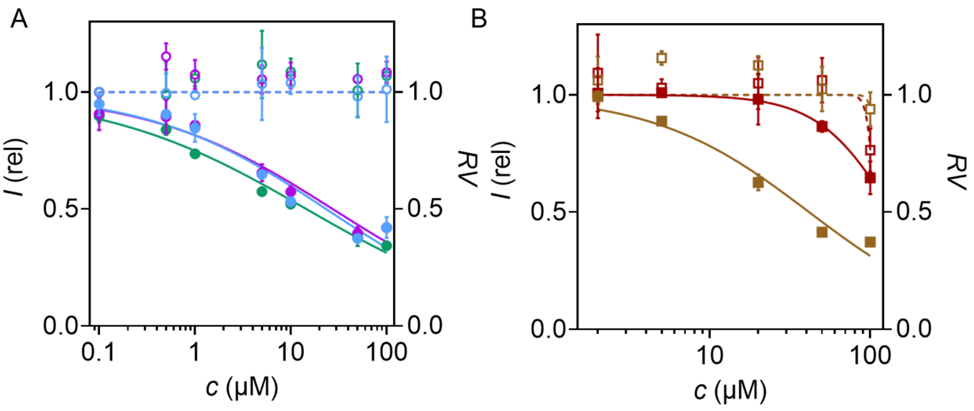

Fig. S10 Automatically analyzed HCHT data showing fluorescence intensity (filled symbols) and relative viability (empty symbols) of HeLa cells after A) pre-incubation with **7** for 30 min (blue circles), 1 h (purple circles) and 2 h (green circles) followed by incubation with **1** (10 µM) for 30 min; B) incubation with **7** for 5.5 h with FBS (brown squares) and 23.5 h with FBS (red squares) followed by co-incubation with **1** (10 µM) for 30 min.

Table S4 Inhibition of cellular uptake of **1** by **7** and cytotoxicity of **7** as a function of assay conditions

Entry	t_{pre}[a] (h)	t_{inc}[b] (h)	t_{co}[c] (h)	MIC[d] (μM)	IC_{50}[e] (μM)	n (IC_{50})[f]	GI_{50}[g] (μM)	n (GI_{50})[h]
1	0.5	0.5	-	0.5	23 ± 4	-0.47 ± 0.04	> 100	-
2	1	0.5	-	0.5	27 ± 4	-0.45 ± 0.04	> 100	-
3	2	0.5	-	0.2	15 ± 1	-0.41 ± 0.02	> 100	-
4	5.5[i]	-	0.5	6	42 ± 4	-0.89 ± 0.08	> 100	-
5	23.5[i]	-	0.5	54	> 100	-	> 100	-

[a]Pre-incubation time of HeLa cells with **7**. [b]Incubation time with **1** after pre-incubation[a] and removal of **7**. [c]Co-incubation time with **7** and **1** after pre-incubation[a] with **7**. [d]Minimum inhibitory concentration of **7**. [e]Half maximal inhibitory concentration of **7**. [f]Hill coefficient for inhibition of cellular uptake. [g]Concentration of **7** causing 50% cell growth inhibition. [h]Hill coefficient for toxicity. [i]Incubation with FBS.

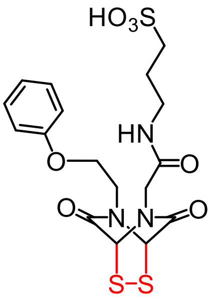

8

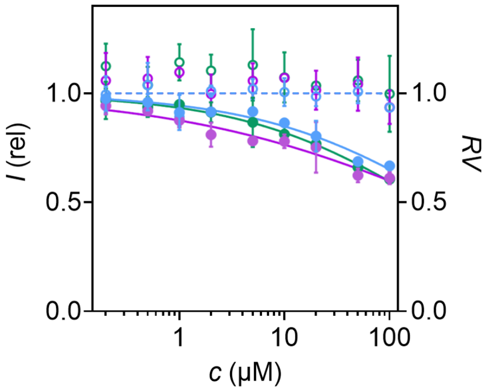

Fig. S11 Automatically analyzed HCHT data showing fluorescence intensity (filled symbols) and relative viability (empty symbols) of HeLa cells after pre-incubation with **8** for 30 min (blue circles), 1 h (purple circles) and 2 h (green circles) followed by incubation with **1** (10 µM) for 30 min.

Table S5 Inhibition of cellular uptake of **1** by **8** and cytotoxicity of **8** as a function of assay conditions

Entry	t_{pre}[a] (h)	t_{inc}[b] (h)	MIC[c] (µM)	IC_{50}[d] (µM)	n (IC_{50})[e]	GI_{50}[f] (µM)	n (GI_{50})[g]
1	0.5	0.5	10	> 100	-	> 100	-
2	1	0.5	2	> 100	-	> 100	-
3	2	0.5	6	> 100	-	> 100	-

[a]Pre-incubation time of HeLa cells with **8**. [b]Incubation time with **1** after pre-incubation[a] and removal of **8**. [c]Minimum inhibitory concentration of **8**. [d]Half maximal inhibitory concentration of **8**. [e]Hill coefficient for inhibition of cellular uptake. [f]Concentration of **8** causing 50% cell growth inhibition. [g]Hill coefficient for toxicity.

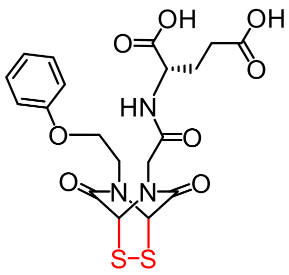

9

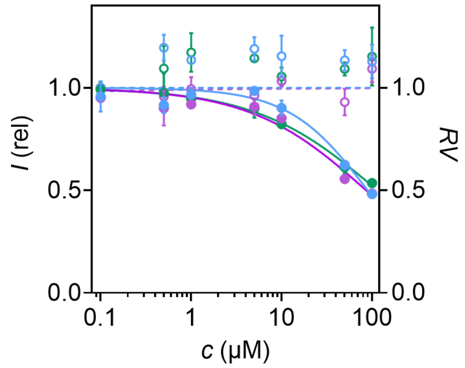

Fig. S12 Automatically analyzed HCHT data showing fluorescence intensity (filled symbols) and relative viability (empty symbols) of HeLa cells after pre-incubation with **9** for 30 min (blue circles), 1 h (purple circles) and 2 h (green circles) followed by incubation with **1** (10 µM) for 30 min.

Table S6 Inhibition of cellular uptake of **1** by **9** and cytotoxicity of **9** as a function of assay conditions

Entry	t_{pre}[a] (h)	t_{inc}[b] (h)	MIC[c] (µM)	IC_{50}[d] (µM)	n (IC_{50})[e]	GI_{50}[f] (µM)	n (GI_{50})[g]
1	0.5	0.5	15	89 ± 9	-1.0 ± 0.1	> 100	-
2	1	0.5	7	86 ± 13	-0.67 ± 0.08	> 100	-
3	2	0.5	7	> 100	-	> 100	-

[a]Pre-incubation time of HeLa cells with **9**. [b]Incubation time with **1** after pre-incubation[a] and removal of **9**. [c]Minimum inhibitory concentration of **9**. [d]Half maximal inhibitory concentration of **9**. [e]Hill coefficient for inhibition of cellular uptake. [f]Concentration of **9** causing 50% cell growth inhibition. [g]Hill coefficient for toxicity.

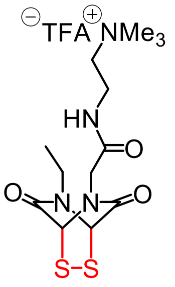

12

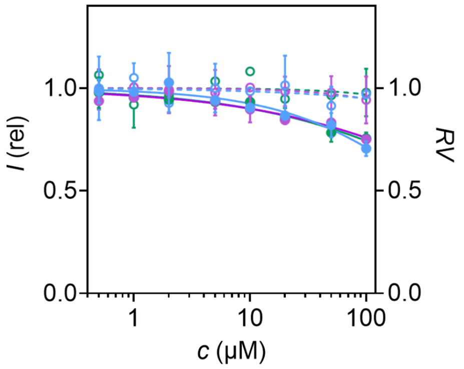

Fig. S13 Automatically analyzed HCHT data showing fluorescence intensity (filled symbols) and relative viability (empty symbols) of HeLa cells after pre-incubation with **12** for 30 min (blue circles), 1 h (purple circles) and 2 h (green circles) followed by incubation with **1** (10 μM) for 30 min.

Table S7 Inhibition of cellular uptake of **1** by **12** and cytotoxicity of **12** as a function of assay conditions

Entry	t_{pre}[a] (h)	t_{inc}[b] (h)	MIC[c] (μM)	IC_{50}[d] (μM)	n (IC_{50})[e]	GI_{50}[f] (μM)	n (GI_{50})[g]
1	0.5	0.5	30	> 100	-	> 100	-
2	1	0.5	30	> 100	-	> 100	-
3	2	0.5	30	> 100	-	> 100	-

[a]Pre-incubation time of HeLa cells with **12**. [b]Incubation time with **1** after pre-incubation[a] and removal of **12**. [c]Minimum inhibitory concentration of **12**. [d]Half maximal inhibitory concentration of **12**. [e]Hill coefficient for inhibition of cellular uptake. [f]Concentration of **12** causing 50% cell growth inhibition. [g]Hill coefficient for toxicity.

4.3.1.2. Screening with γ-Turn Peptides as Inhibitors

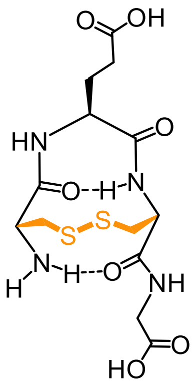

18

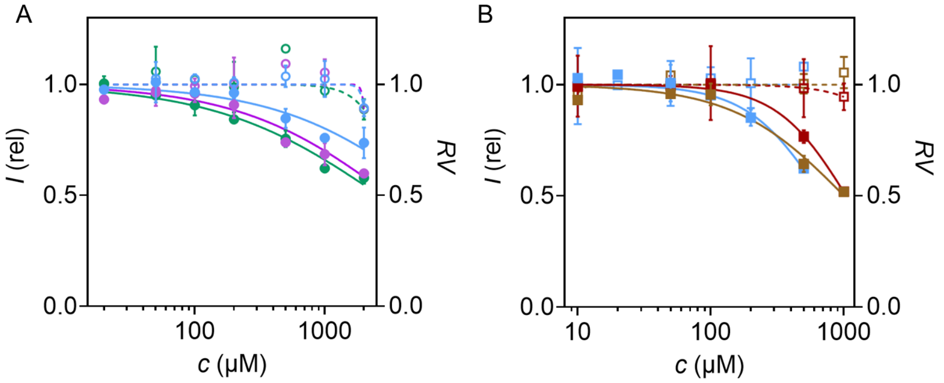

Fig. S14 Automatically analyzed HCHT data showing fluorescence intensity (filled symbols) and relative viability (empty symbols) of HeLa cells after A) pre-incubation with **18** for 30 min (blue circles), 1 h (purple circles) and 2 h (green circles) followed by incubation with **1** (10 µM) for 30 min; B) incubation with **18** for 30 min (blue sqiares), 5.5 h with FBS (brown squares) and 23.5 h with FBS (red squares) followed by co-incubation with **1** (10 µM) for 30 min.

Table S8 Inhibition of cellular uptake of **1** by **18** and cytotoxicity of **18** as a function of assay conditions

Entry	t_{pre}[a] (h)	t_{inc}[b] (h)	t_{co}[c] (h)	MIC[d] (μM)	IC_{50}[e] (mM)	n (IC_{50})[f]	GI_{50}[g] (mM)	n (GI_{50})[h]
1	0.5	0.5	-	600	> 2	-	> 2	-
2	1	0.5	-	300	> 2	-	> 2	-
3	2	0.5	-	200	> 2	-	> 2	-
4	0.5	-	0.5	200	> 0.5	-	> 0.5	-
5	5.5[i]	-	0.5	200	1.00 ± 0.11	-1.0 ± 0.1	> 1	-
6	23.5[i]	-	0.5	400	1.00 ± 0.04	-1.6 ± 0.1	> 1	-

[a]Pre-incubation time of HeLa cells with **18**. [b]Incubation time with **1** after pre-incubation[a] and removal of **18**. [c]Co-incubation time with **18** and **1** after pre-incubation[a] with **18**. [d]Minimum inhibitory concentration of **18**. [e]Half maximal inhibitory concentration of **18**. [f]Hill coefficient for inhibition of cellular uptake. [g]Concentration of **18** causing 50% cell growth inhibition. [h]Hill coefficient for toxicity. [i]Incubation with FBS.

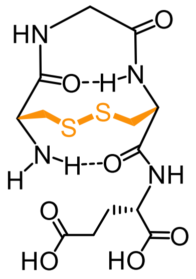

19

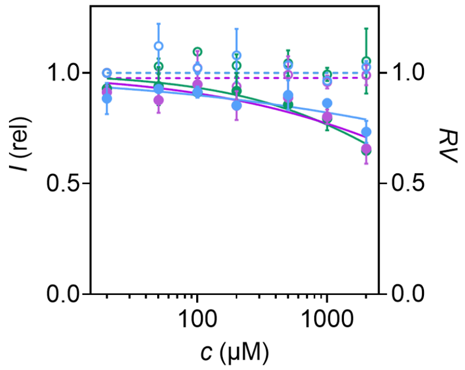

Fig. S15 Automatically analyzed HCHT data showing fluorescence intensity (filled symbols) and relative viability (empty symbols) of HeLa cells after pre-incubation with **19** for 30 min (blue circles), 1 h (purple circles) and 2 h (green circles) followed by incubation with **1** (10 µM) for 30 min.

Table S9 Inhibition of cellular uptake of **1** by **19** and cytotoxicity of **19** as a function of assay conditions

Entry	t_{pre}[a] (h)	t_{inc}[b] (h)	MIC[c] (µM)	IC_{50}[d] (mM)	n (IC_{50})[e]	GI_{50}[f] (mM)	n (GI_{50})[g]
1	0.5	0.5	400	> 2	-	> 2	-
2	1	0.5	400	> 2	-	> 2	-
3	2	0.5	400	> 2	-	> 2	-

[a]Pre-incubation time of HeLa cells with **19**. [b]Incubation time with **1** after pre-incubation[a] and removal of **19**. [c]Minimum inhibitory concentration of **19**. [d]Half maximal inhibitory concentration of **19**. [e]Hill coefficient for inhibition of cellular uptake. [f]Concentration of **19** causing 50% cell growth inhibition. [g]Hill coefficient for toxicity.

4.3.1.3. Screening with Cyclic Disulfides as Inhibitors

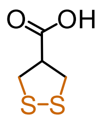

28

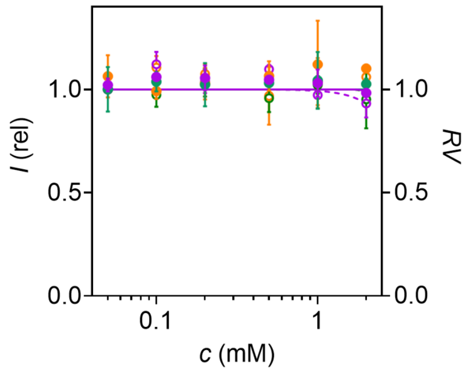

Fig. S16 Automatically analyzed HCHT data showing fluorescence intensity (filled symbols) and relative viability (empty symbols) of HeLa cells after pre-incubation with **28** for 1 h (purple circles), 2 h (green circles), and 4 h (yellow circles) followed by incubation with **1** (10 µM) for 30 min.

Table S10 Inhibition of cellular uptake of **1** by **28** and cytotoxicity of **28** as a function of assay conditions

Entry	t_{pre}[a] (h)	t_{inc}[b] (h)	MIC[c] (mM)	IC_{50}[d] (mM)	n (IC_{50})[e]	GI_{50}[f] (mM)	n (GI_{50})[g]
1	1	0.5	> 2	> 2	-	> 2	-
2	2	0.5	> 2	> 2	-	> 2	-
3	4	0.5	> 2	> 2	-	> 2	-

[a]Pre-incubation time of HeLa cells with **28**. [b]Incubation time with **1** after pre-incubation[a] and removal of **28**. [c]Minimum inhibitory concentration of **28**. [d]Half maximal inhibitory

concentration of **28**. [e]Hill coefficient for inhibition of cellular uptake. [f]Concentration of **28** causing 50% cell growth inhibition. [g]Hill coefficient for toxicity.

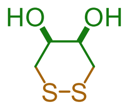

29

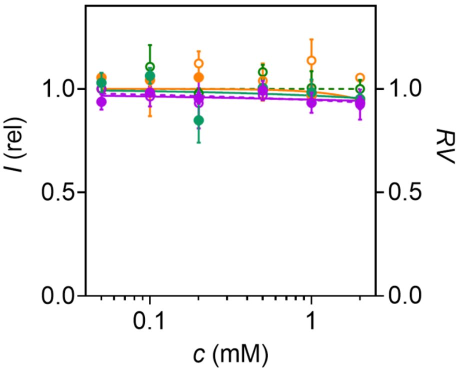

Fig. S17 Automatically analyzed HCHT data showing fluorescence intensity (filled symbols) and relative viability (empty symbols) of HeLa cells after pre-incubation with **29** for 1 h (purple circles), 2 h (green circles), and 4 h (yellow circles) followed by incubation with **1** (10 µM) for 30 min.

Table S11 Inhibition of cellular uptake of **1** by **29** and cytotoxicity of **29** as a function of assay conditions

Entry	t_{pre}[a] (h)	t_{inc}[b] (h)	MIC[c] (mM)	IC_{50}[d] (mM)	n (IC_{50})[e]	GI_{50}[f] (mM)	n (GI_{50})[g]
1	1	0.5	> 2	> 2	-	> 2	-
2	2	0.5	> 2	> 2	-	> 2	-
3	4	0.5	> 2	> 2	-	> 2	-

[a]Pre-incubation time of HeLa cells with **29**. [b]Incubation time with **1** after pre-incubation[a] and removal of **29**. [c]Minimum inhibitory concentration of **29**. [d]Half maximal inhibitory

concentration of **29**. [e]Hill coefficient for inhibition of cellular uptake. [f]Concentration of **29** causing 50% cell growth inhibition. [g]Hill coefficient for toxicity.

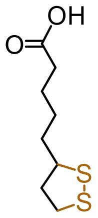

30

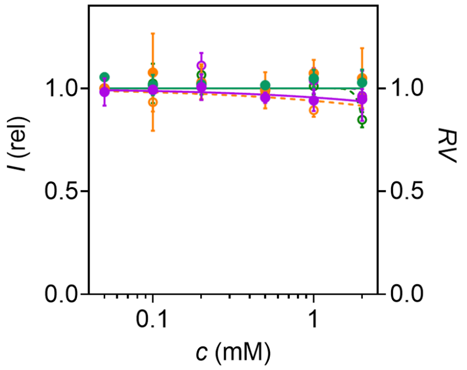

Fig. S18 Automatically analyzed HCHT data showing fluorescence intensity (filled symbols) and relative viability (empty symbols) of HeLa cells after pre-incubation with **30** for 1 h (purple circles), 2 h (green circles), and 4 h (yellow circles) followed by incubation with **1** (10 μM) for 30 min.

Table S12 Inhibition of cellular uptake of **1** by **30** and cytotoxicity of **30** as a function of assay conditions

Entry	t_{pre}[a] (h)	t_{inc}[b] (h)	MIC[c] (mM)	IC_{50}[d] (mM)	n (IC_{50})[e]	GI_{50}[f] (mM)	n (GI_{50})[g]
1	1	0.5	> 2	> 2	-	> 2	-
2	2	0.5	> 2	> 2	-	> 2	-
3	4	0.5	> 2	> 2	-	> 2	-

[a]Pre-incubation time of HeLa cells with **30**. [b]Incubation time with **1** after pre-incubation[a] and removal of **30**. [c]Minimum inhibitory concentration of **30**. [d]Half maximal inhibitory

concentration of **30**. [e]Hill coefficient for inhibition of cellular uptake. [f]Concentration of **30** causing 50% cell growth inhibition. [g]Hill coefficient for toxicity.

4.3.1.4. Screening with DTNB as Inhibitor

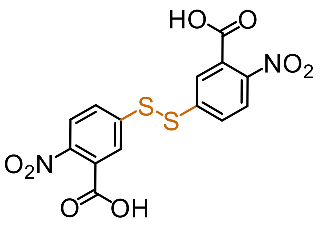

20

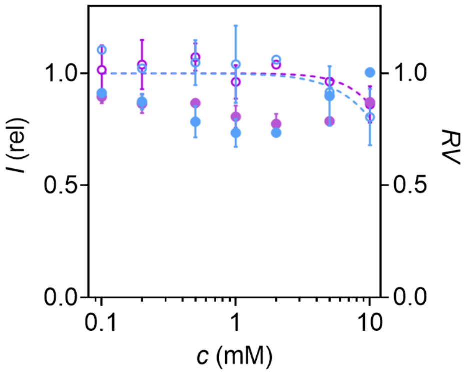

Fig. S19 Automatically analyzed HCHT data showing fluorescence intensity (filled symbols) and relative viability (empty symbols) of HeLa cells after pre-incubation with **20** (pre-neutralized) for 30 min (blue circles) and 1 h (purple circles) followed by incubation with **1** (10 µM) for 30 min.

Table S13 Inhibition of cellular uptake of **1** by **20** and cytotoxicity of **20** as a function of assay conditions

Entry	t_{pre}[a] (h)	t_{inc}[b] (h)	MIC[c] (mM)	IC_{50}[d] (mM)	n (IC_{50})[e]	GI_{50}[f] (mM)	n (GI_{50})[g]
1	0.5	0.5	0.2	-	-	> 10	-
2	1	0.5	0.5	-	-	> 10	-

[a]Pre-incubation time of HeLa cells with **20**. [b]Incubation time with **1** after pre-incubation[a] and removal of **20**. [c]Minimum inhibitory concentration of **20**. [d]Half maximal inhibitory concentration of **20**. [e]Hill coefficient for inhibition of cellular uptake. [f]Concentration of **20** causing 50% cell growth inhibition. [g]Hill coefficient for toxicity

4.3.2. Screening with BPS as Inhibitors

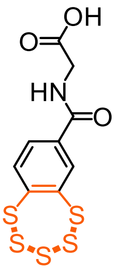

6

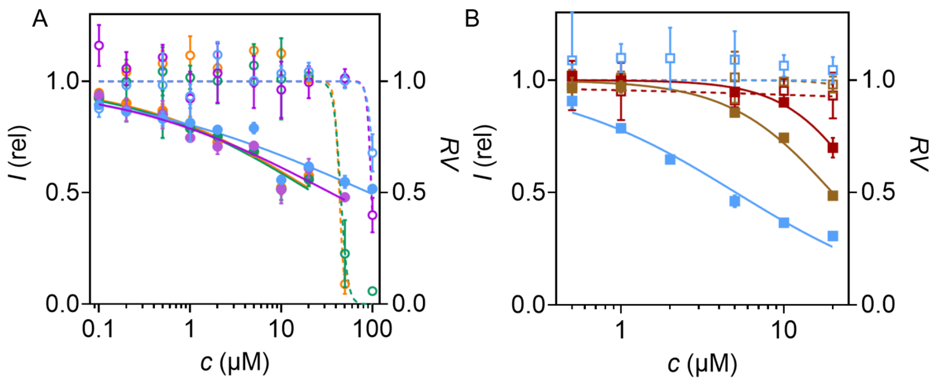

Fig. S20 Automatically analyzed HCHT data showing fluorescence intensity (filled symbols) and relative viability (empty symbols) of HeLa cells after A) pre-incubation with **6** for 30 min (blue circles), 1 h (purple circles), 2 h (green circles) and 4 h (yellow circles) followed by incubation with **1** (10 µM) for 30 min; B) incubation with **6** for 30 min (blue squares), 5.5 h with FBS (brown squares) and 23.5 h with FBS (red squares) followed by co-incubation with **1** (10 µM) for 30 min.

Table S14 Inhibition of cellular uptake of **1** by **6** and cytotoxicity of **6** as a function of assay conditions

Entry	t_{pre}[a] (h)	t_{inc}[b] (h)	t_{co}[c] (h)	MIC[d] (μM)	IC_{50}[e] (μM)	n (IC_{50})[f]	GI_{50}[g] (μM)	n (GI_{50})[h]
1	0.5	0.5	-	0.3	93 ± 28	-0.31 ± 0.03	> 100	-
2	1	0.5	-	0.3	35 ± 9	-0.37 ± 0.04	~ 98	-
3	2	0.5	-	0.4	23 ± 6	-0.43 ± 0.05	~ 45	-
4	4	0.5	-	0.4	24 ± 5	-0.44 ± 0.04	~ 44	-
5	0.5	-	0.5	0.5	5.1 ± 0.3	-0.77 ± 0.05	> 20	-
6	5.5[i]	-	0.5	5	20 ± 1	-1.37 ± 0.11	> 20	-
7	23.5[i]	-	0.5	12	> 20	-	> 20	-

[a]Pre-incubation time of HeLa cells with **6**. [b]Incubation time with **1** after pre-incubation[a] and removal of **6**. [c]Co-incubation time with **6** and **1** after pre-incubation[a] with **6**. [d]Minimum inhibitory concentration of **6**. [e]Half maximal inhibitory concentration of **6**. [f]Hill coefficient for inhibition of cellular uptake. [g]Concentration of **6** causing 50% cell growth inhibition. [h]Hill coefficient for toxicity. [i]Incubation with FBS.

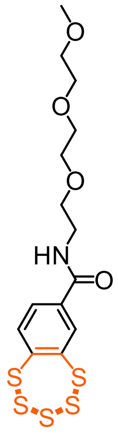

10

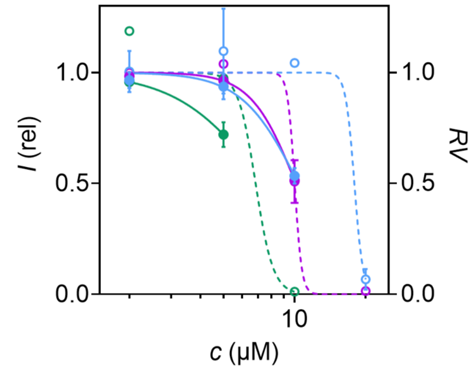

Fig. S21 Automatically analyzed HCHT data showing fluorescence intensity (filled symbols) and relative viability (empty symbols) of HeLa cells after pre-incubation with **10** for 30 min (blue circles), 1 h (purple circles), and 2 h (green circles) followed by incubation with **1** (10 µM) for 30 min.

Table S15 Inhibition of cellular uptake of **1** by **10** and cytotoxicity of **10** as a function of assay conditions

Entry	t_{pre}[a] (h)	t_{inc}[b] (h)	MIC[c] (µM)	IC_{50}[d] (µM)	n (IC_{50})[e]	GI_{50}[f] (µM)	n (GI_{50})[g]
1	0.5	0.5	6.5	10.4 ± 0.3	-3.6 ± 0.6	~ 18	-
2	1	0.5	7	10.1 ± 0.1	-4.6 ± 0.5	~ 10	-
3	2	0.5	3.5	> 5	-	~ 7	

[a]Pre-incubation time of HeLa cells with **10**. [b]Incubation time with **1** after pre-incubation[a] and removal of **10**. [c]Minimum inhibitory concentration of **10**. [d]Half maximal inhibitory

concentration of **10**. [e]Hill coefficient for inhibition of cellular uptake. [f]Concentration of **10** causing 50% cell growth inhibition. [g]Hill coefficient for toxicity.

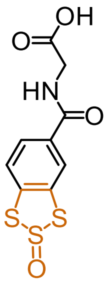

24

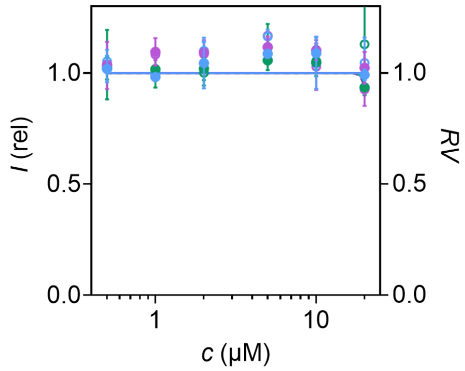

Fig. S22 Automatically analyzed HCHT data showing fluorescence intensity (filled symbols) and relative viability (empty symbols) of HeLa cells after pre-incubation with **24** for 30 min (blue circles), 1 h (purple circles), and 2 h (green circles) followed by incubation with **1** (10 μM) for 30 min.

Table S16 Inhibition of cellular uptake of **1** by **24** and cytotoxicity of **24** as a function of assay conditions

Entry	t_{pre}[a] (h)	t_{inc}[b] (h)	MIC[c] (μM)	IC_{50}[d] (μM)	n (IC_{50})[e]	GI_{50}[f] (μM)	n (GI_{50})[g]
1	0.5	0.5	> 20	> 20	-	> 20	-
2	1	0.5	> 20	> 20	-	> 20	-
3	2	0.5	> 20	> 20	-	> 20	-

[a]Pre-incubation time of HeLa cells with **24**. [b]Incubation time with **1** after pre-incubation[a] and removal of **24**. [c]Minimum inhibitory concentration of **24**. [d]Half maximal inhibitory

concentration of **24**. [e]Hill coefficient for inhibition of cellular uptake. [f]Concentration of **24** causing 50% cell growth inhibition. [g]Hill coefficient for toxicity.

4.3.3. Screening with Heteroaromatic Sulfones as Inhibitors

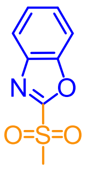

11

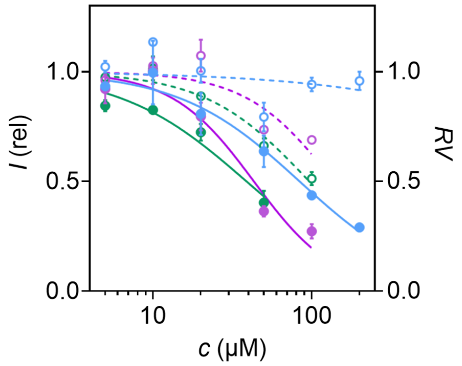

Fig. S23 Automatically analyzed HCHT data showing fluorescence intensity (filled symbols) and relative viability (empty symbols) of HeLa cells after pre-incubation with **11** for 15 min (blue circles), 30 min (purple circles), and 1 h (green circles) followed by incubation with **1** (10 µM) for 30 min.

Table S17 Inhibition of cellular uptake of **1** by **11** and cytotoxicity of **11** as a function of assay conditions

Entry	t_{pre}[a] (h)	t_{inc}[b] (h)	MIC[c] (µM)	IC_{50}[d] (µM)	n (IC_{50})[e]	GI_{50}[f] (µM)	n (GI_{50})[g]
1	0.25	0.5	18	84.0 ± 8.2	-1.1 ± 0.1	> 200	-
2	0.5	0.5	15	43.3 ± 3.9	-1.7 ± 0.2	138 ± 42	-1.6 ± 0.6
3	1	0.5	8	38.9 ± 4.2	-1.1 ± 0.1	97 ± 8	-1.3 ± 0.2

[a]Pre-incubation time of HeLa cells with **11**. [b]Incubation time with **1** after pre-incubation[a] and removal of **11**. [c]Minimum inhibitory concentration of **11**. [d]Half maximal inhibitory concentration of **11**. [e]Hill coefficient for inhibition of cellular uptake. [f]Concentration of **11** causing 50% cell growth inhibition. [g]Hill coefficient for toxicity.

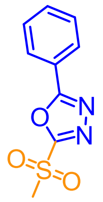

14

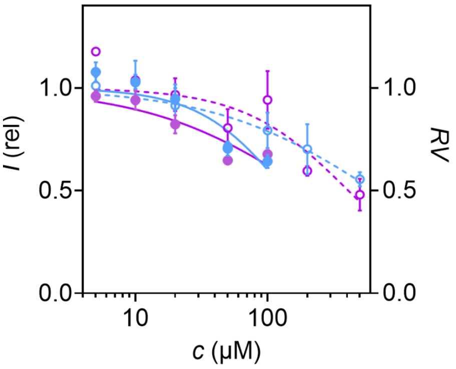

Fig. S24 Automatically analyzed HCHT data showing fluorescence intensity (filled symbols) and relative viability (empty symbols) of HeLa cells after pre-incubation with **14** for 15 min (blue circles) and 30 min (purple circles) followed by incubation with **1** (10 μM) for 30 min.

Table S18 Inhibition of cellular uptake of **1** by **14** and cytotoxicity of **14** as a function of assay conditions

Entry	t_{pre}[a] (h)	t_{inc}[b] (h)	MIC[c] (μM)	IC_{50}[d] (μM)	n (IC_{50})[e]	GI_{50}[f] (μM)	n (GI_{50})[g]
1	0.25	0.5	40	137 ± 28	-1.3 ± 0.3	> 500	-
2	0.5	0.5	20	204 ± 63	-0.7 ± 0.1	410 ± 99	-1.1 ± 0.3

[a]Pre-incubation time of HeLa cells with **14**. [b]Incubation time with **1** after pre-incubation[a] and removal of **14**. [c]Minimum inhibitory concentration of **14**. [d]Half maximal inhibitory

concentration of **14**. [e]Hill coefficient for inhibition of cellular uptake. [f]Concentration of **14** causing 50% cell growth inhibition. [g]Hill coefficient for toxicity.

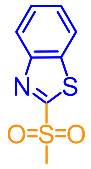

21

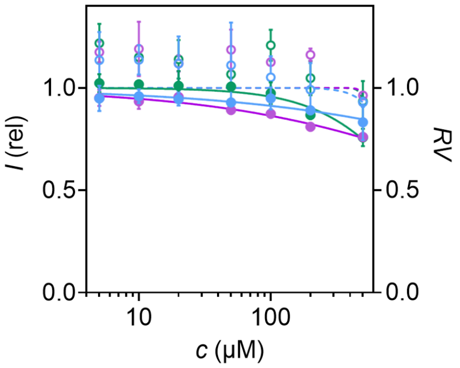

Fig. S25 Automatically analyzed HCHT data showing fluorescence intensity (filled symbols) and relative viability (empty symbols) of HeLa cells after pre-incubation with **21** for 15 min (blue circles), 30 min (purple circles), and 1 h (green circles) followed by incubation with **1** (10 μM) for 30 min.

Table S19 Inhibition of cellular uptake of **1** by **21** and cytotoxicity of **21** as a function of assay conditions

Entry	t_{pre}[a] (h)	t_{inc}[b] (h)	MIC[c] (μM)	IC_{50}[d] (μM)	n (IC_{50})[e]	GI_{50}[f] (μM)	n (GI_{50})[g]
1	0.25	0.5	300	> 500	-	> 500	-
2	0.5	0.5	100	> 500	-	> 500	-
3	1	0.5	300	> 500	-	> 500	-

[a]Pre-incubation time of HeLa cells with **21**. [b]Incubation time with **1** after pre-incubation[a] and removal of **21**. [c]Minimum inhibitory concentration of **21**. [d]Half maximal inhibitory

concentration of **21**. [e]Hill coefficient for inhibition of cellular uptake. [f]Concentration of **21** causing 50% cell growth inhibition. [g]Hill coefficient for toxicity.

4.3.4. Screening with Thiosulfinates as Inhibitors

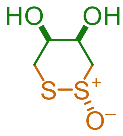

17

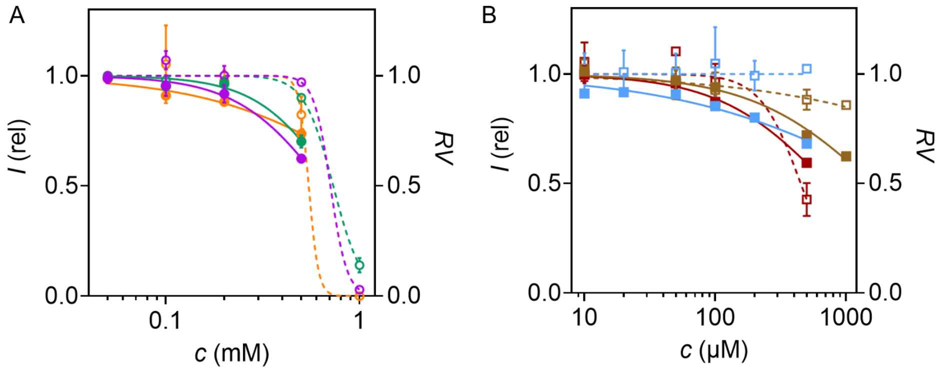

Fig. S26 Automatically analyzed HCHT data showing fluorescence intensity (filled symbols) and relative viability (empty symbols) of HeLa cells after A) pre-incubation with **17** for 1 h (purple circles), 2 h (green circles) and 4 h (yellow circles) followed by incubation with **1** (10 μM) for 30 min; B) incubation with **17** for 30 min (blue squares), 5.5 h with FBS (brown squares) and 23.5 h with FBS (red squares) followed by co-incubation with **1** (10 μM) for 30 min.

Table S20 Inhibition of cellular uptake of **1** by **17** and cytotoxicity of **17** as a function of assay conditions

Entry	t_{pre}[a] (h)	t_{inc}[b] (h)	t_{co}[c] (h)	MIC[d] (μM)	IC_{50}[e] (mM)	n (IC_{50})[f]	GI_{50}[g] (mM)	n (GI_{50})[h]
1	1	0.5	-	250	> 0.5	-	0.71 ± 0.05	-10.1 ± 1.9
2	2	0.5	-	300	> 0.5	-	0.73 ± 0.01	-5.8 ± 0.3
3	4	0.5	-	250	> 0.5	-	~ 0.5	-
4	0.5	-	0.5	100	> 0.5	-	> 0.5	-
5	5.5[i]	-	0.5	250	> 1.0	-	> 1	-
6	23.5[i]	-	0.5	150	> 0.5	-	0.45 ± 0.05	-2.8 ± 2.4

[a]Pre-incubation time of HeLa cells with **17**. [b]Incubation time with **1** after pre-incubation[a] and removal of **17**. [c]Co-incubation time with **17** and **1** after pre-incubation[a] with **17**. [d]Minimum inhibitory concentration of **17**. [e]Half maximal inhibitory concentration of **17**. [f]Hill coefficient for inhibition of cellular uptake. [g]Concentration of **17** causing 50% cell growth inhibition. [h]Hill coefficient for toxicity. [i]Incubation with FBS.

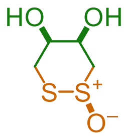

22

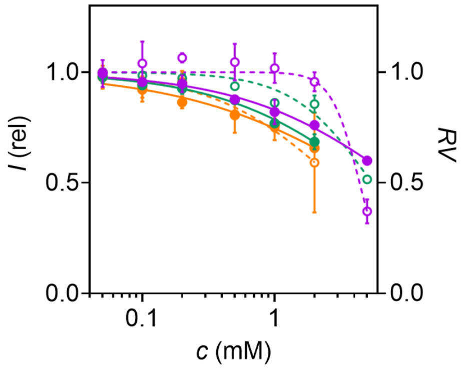

Fig. S27 Automatically analyzed HCHT data showing fluorescence intensity (filled symbols) and relative viability (empty symbols) of HeLa cells after pre-incubation with **22** for 1 h (purple circles), 2 h (green circles) and 4 h (yellow circles) followed by incubation with **1** (10 µM) for 30 min.

Table S21 Inhibition of cellular uptake of **1** by **22** and cytotoxicity of **22** as a function of assay conditions

Entry	t_{pre}[a] (h)	t_{inc}[b] (h)	MIC[c] (mM)	IC_{50}[d] (mM)	n (IC_{50})[e]	GI_{50}[f] (mM)	n (GI_{50})[g]
1	1	0.5	0.8	> 5	-	4.4 ± 0.2	-4.0 ± 1.1
2	2	0.5	0.6	> 2	-	> 5	-
3	4	0.5	0.3	> 2	-	> 2	-

[a]Pre-incubation time of HeLa cells with **22**. [b]Incubation time with **1** after pre-incubation[a] and removal of **22**. [c]Minimum inhibitory concentration of **22**. [d]Half maximal inhibitory concentration of **22**. [e]Hill coefficient for inhibition of cellular uptake. [f]Concentration of **22** causing 50% cell growth inhibition. [g]Hill coefficient for toxicity.

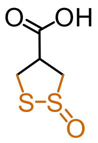

23

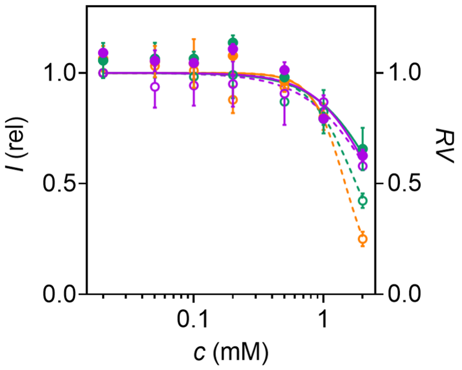

Fig. S28 Automatically analyzed HCHT data showing fluorescence intensity (filled symbols) and relative viability (empty symbols) of HeLa cells after pre-incubation with **23** for 1 h (purple circles), 2 h (green circles) and 4 h (yellow circles) followed by incubation with **1** (10 µM) for 30 min.

Table S22 Inhibition of cellular uptake of **1** by **23** and cytotoxicity of **23** as a function of assay conditions

Entry	t_{pre}[a] (h)	t_{inc}[b] (h)	MIC[c] (mM)	IC_{50}[d] (mM)	n (IC_{50})[e]	GI_{50}[f] (mM)	n (GI_{50})[g]
1	1	0.5	1.0	> 2	-	> 2	-
2	2	0.5	1.0	> 2	-	1.78 ± 0.12	-2.4 ± 0.5
3	4	0.5	0.9	> 1	-	1.47 ± 0.07	-3.4 ± 0.4

[a]Pre-incubation time of HeLa cells with **23**. [b]Incubation time with **1** after pre-incubation[a] and removal of **23**. [c]Minimum inhibitory concentration of **23**. [d]Half maximal inhibitory concentration of **23**. [e]Hill coefficient for inhibition of cellular uptake. [f]Concentration of **23** causing 50% cell growth inhibition. [g]Hill coefficient for toxicity.

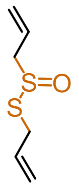

25

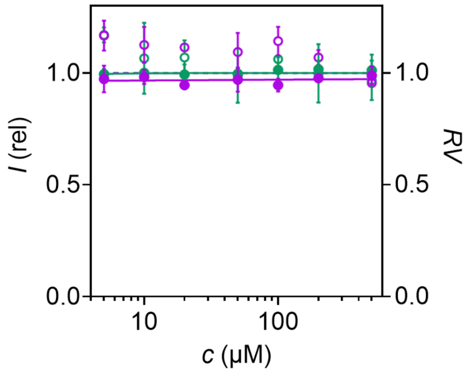

Fig. S29 Automatically analyzed HCHT data showing fluorescence intensity (filled symbols) and relative viability (empty symbols) of HeLa cells after pre-incubation with **25** for 1 h (purple circles) and 2 h (green circles) followed by incubation with **1** (10 µM) for 30 min.

Table S23 Inhibition of cellular uptake of **1** by **25** and cytotoxicity of **25** as a function of assay conditions

Entry	t_{pre}[a] (h)	t_{inc}[b] (h)	MIC[c] (µM)	IC_{50}[d] (µM)	n (IC_{50})[e]	GI_{50}[f] (µM)	n (GI_{50})[g]
1	1	0.5	> 500	> 500	-	> 500	-
2	2	0.5	> 500	> 500	-	> 500	-

[a]Pre-incubation time of HeLa cells with **25**. [b]Incubation time with **1** after pre-incubation[a] and removal of **25**. [c]Minimum inhibitory concentration of **25**. [d]Half maximal inhibitory concentration of **25**. [e]Hill coefficient for inhibition of cellular uptake. [f]Concentration of **25** causing 50% cell growth inhibition. [g]Hill coefficient for toxicity.

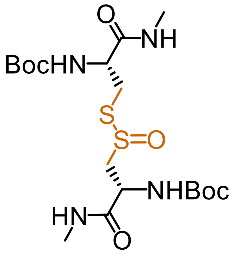

26

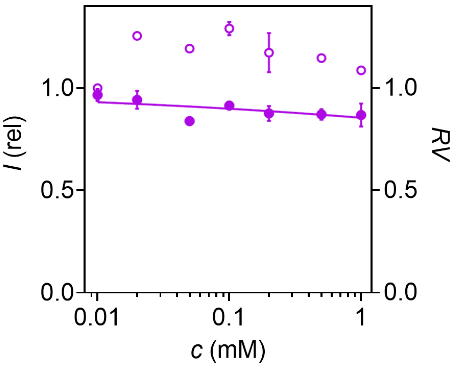

Fig. S30 Automatically analyzed HCHT data showing fluorescence intensity (filled symbols) and relative viability (empty symbols) of HeLa cells after pre-incubation with **26** for 1 h (purple circles) followed by incubation with **1** (10 µM) for 30 min.

Table S24 Inhibition of cellular uptake of **1** by **26** and cytotoxicity of **26** as a function of assay conditions

Entry	t_{pre}[a] (h)	t_{inc}[b] (h)	MIC[c] (mM)	IC_{50}[d] (mM)	n (IC_{50})[e]	GI_{50}[f] (mM)	n (GI_{50})[g]
1	1	0.5	> 1	> 1	-	> 1	-

[a]Pre-incubation time of HeLa cells with **26**. [b]Incubation time with **1** after pre-incubation[a] and removal of **26**. [c]Minimum inhibitory concentration of **26**. [d]Half maximal inhibitory concentration of **26**. [e]Hill coefficient for inhibition of cellular uptake. [f]Concentration of **26** causing 50% cell growth inhibition. [g]Hill coefficient for toxicity.

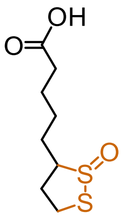

27

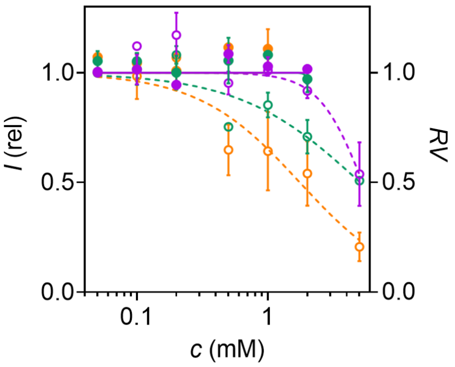

Fig. S31 Automatically analyzed HCHT data showing fluorescence intensity (filled symbols) and relative viability (empty symbols) of HeLa cells after pre-incubation with **27** for 1 h (purple circles), 2 h (green circles) and 4 h (yellow circles) followed by incubation with **1** (10 µM) for 30 min.

Table S25 Inhibition of cellular uptake of **1** by **27** and cytotoxicity of **27** as a function of assay conditions

Entry	t_{pre}[a] (h)	t_{inc}[b] (h)	MIC[c] (mM)	IC_{50}[d] (mM)	n (IC_{50})[e]	GI_{50}[f] (mM)	n (GI_{50})[g]
1	1	0.5	> 2	> 2	-	> 5	-
2	2	0.5	> 2	> 2	-	> 5	-
3	4	0.5	> 2	> 2	-	1.8 ± 0.3	-1.1 ± 0.2

[a]Pre-incubation time of HeLa cells with **27**. [b]Incubation time with **1** after pre-incubation[a] and removal of **27**. [c]Minimum inhibitory concentration of **27**. [d]Half maximal inhibitory concentration of **27**. [e]Hill coefficient for inhibition of cellular uptake. [f]Concentration of **27** causing 50% cell growth inhibition. [g]Hill coefficient for toxicity.

4.3.5. Screening with Thiosulfonates as Inhibitors

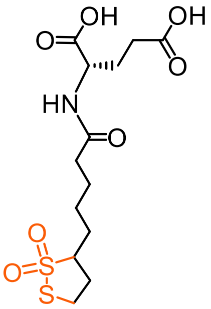

15

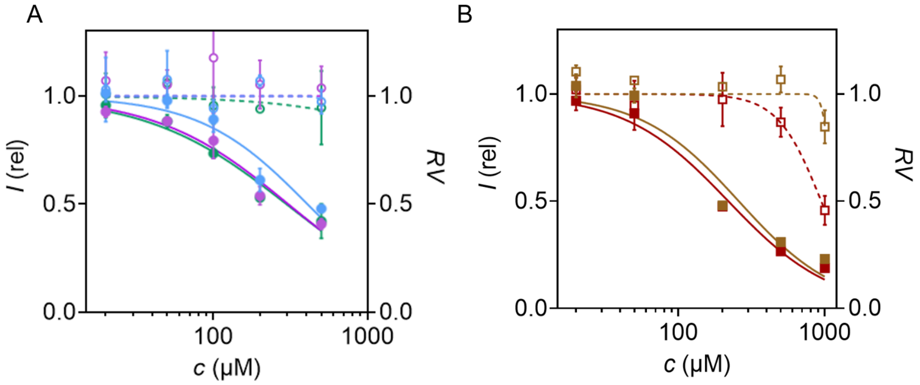

Fig. S32 Automatically analyzed HCHT data showing fluorescence intensity (filled symbols) and relative viability (empty symbols) of HeLa cells after A) pre-incubation with **15** for 30 min (blue circles), 1 h (purple circles) and 2 h (green circles) followed by incubation with **1** (10 µM) for 30 min; B) incubation with **15** for 5.5 h with FBS (brown squares) and 23.5 h with FBS (red squares) followed by co-incubation with **1** (10 µM) for 30 min.

Table S26 Inhibition of cellular uptake of **1** by **15** and cytotoxicity of **15** as a function of assay conditions

Entry	t_{pre}[a] (h)	t_{inc}[b] (h)	t_{co}[c] (h)	MIC[d] (μM)	IC_{50}[e] (μM)	n (IC_{50})[f]	GI_{50}[g] (μM)	n (GI_{50})[h]
1	0.5	0.5	-	100	407 ± 49	-1.2 ± 0.2	> 500	-
2	1	0.5	-	50	306 ± 30	-1.0 ± 0.1	> 500	-
3	2	0.5	-	50	298 ± 31	-1.0 ± 0.1	> 500	-
4	5.5[i]	-	0.5	65	258 ± 33	-1.3 ± 0.2	> 1000	-
5	23.5[i]	-	0.5	55	219 ± 16	-1.3 ± 0.1	944 ± 60	-2.9 ± 0.7

[a]Pre-incubation time of HeLa cells with **15**. [b]Incubation time with **1** after pre-incubation[a] and removal of **15**. [c]Co-incubation time with **15** and **1** after pre-incubation[a] with **15**. [d]Minimum inhibitory concentration of **15**. [e]Half maximal inhibitory concentration of **15**. [f]Hill coefficient for inhibition of cellular uptake. [g]Concentration of **15** causing 50% cell growth inhibition. [h]Hill coefficient for toxicity. [i]Incubation with FBS.

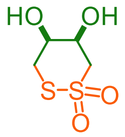

16

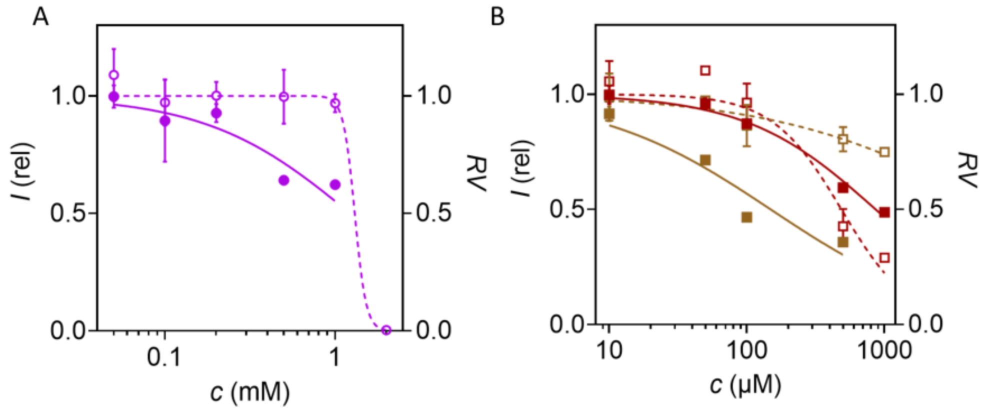

Fig. S33 Automatically analyzed HCHT data showing fluorescence intensity (filled symbols) and relative viability (empty symbols) of HeLa cells after A) pre-incubation with **16** for 1 h (purple circles) followed by incubation with **1** (10 μM) for 30 min; B) incubation with **16** for 5.5 h with FBS (brown squares) and 23.5 h with FBS (red squares) followed by co-incubation with **1** (10 μM) for 30 min.

Table S27 Inhibition of cellular uptake of **1** by **16** and cytotoxicity of **16** as a function of assay conditions

Entry	t_{pre}[a] (h)	t_{inc}[b] (h)	t_{co}[c] (h)	MIC[d] (μM)	IC_{50}[e] (mM)	n (IC_{50})[f]	GI_{50}[g] (mM)	n (GI_{50})[h]
1	1	0.5	-	200	> 1	-	1.30 ± 0.49	-13 ± 18
2	5.5[i]	-	0.5	10	0.15 ± 0.03	-0.7 ± 0.1	> 1	-
3	23.5[i]	-	0.5	150	0.86 ± 0.06	-0.9 ± 0.1	0.49 ± 0.06	-1.7 ± 0.4

[a]Pre-incubation time of HeLa cells with **16**. [b]Incubation time with **1** after pre-incubation[a] and removal of **16**. [c]Co-incubation time with **16** and **1** after pre-incubation[a] with **16**. [d]Minimum inhibitory concentration of **16**. [e]Half maximal inhibitory concentration of **16**. [f]Hill coefficient for inhibition of cellular uptake. [g]Concentration of **16** causing 50% cell growth inhibition. [h]Hill coefficient for toxicity. [i]Incubation with FBS.

4.3.6. Screening with IPS as Inhibitor

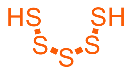

13

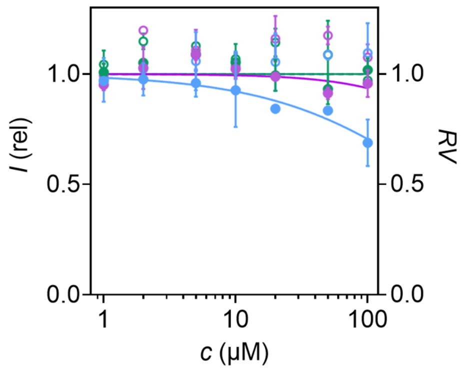

Fig. S34 Automatically analyzed HCHT data showing fluorescence intensity (filled symbols) and relative viability (empty symbols) of HeLa cells after pre-incubation with **13** for 15 min (blue circles), 30 min (pirple circles) and 1 h (green circles) followed by incubation with **1** (10 μM) for 30 min.

Table S28 Inhibition of cellular uptake of **1** by **13** and cytotoxicity of **13** as a function of assay conditions.

Entry	t_{pre}[a] (h)	t_{inc}[b] (h)	MIC[c] (μM)	IC_{50}[d] (μM)	n (IC_{50})[e]	GI_{50}[f] (μM)	n (GI_{50})[g]
1	0.25	0.5	20	> 100	-	> 100	-
2	0.5	0.5	> 100	> 100	-	> 100	-
3	1	0.5	> 100	> 100	-	> 100	-

[a]Pre-incubation time of HeLa cells with **13**. [b]Incubation time with **1** after pre-incubation[a] and removal of **13**. [c]Minimum inhibitory concentration of **13**. [d]Half maximal inhibitory concentration of **13**. [e]Hill coefficient for inhibition of cellular uptake. [f]Concentration of **13** causing 50% cell growth inhibition. [g]Hill coefficient for toxicity.

4.4. HCHT Inhibitor Screening with BPS as Reporter

Integrated fluorescent intensity values (per cell) for each condition with inhibitors were normalized using the value of integrated fluorescent intensity (per cell) with addition of reporter **2**, Hoechst 33342 and PI as maximum signal (I_{rel} = 1) and the value of integrated fluorescent intensity (per cell) with only addition of Hoechst 33342 and PI as minimum (I_{rel} = 0), for each set of experiments. Duplicates were performed for each condition. The resulting dependence of the relative fluorescent intensity values (I_{rel}) to the concentration of inhibitors ($c_{inhibitor}$) was plotted and fitted with Equation (S1) to retrieve the half maximal inhibitory concentration (IC_{50}) value and the Hill coefficient (n).

$$I_{rel} = 1 / (1 + (IC_{50} / c_{inhibitor})^n) \qquad (S1)$$

Relative cell viability (RV) for each condition with inhibitors were calculated using the count value of living cells by dividing the count value of living cells with addition of reporter **2**, Hoechst 33342 and PI, for each set of experiments. Duplicates were performed for each condition. The resulting dependence of the relative cell viability (RV_{rel}) to the concentration of inhibitors ($c_{inhibitor}$) was plotted and fitted with Equation (S2) to retrieve the concentration causing 50% cell growth inhibition (GI_{50}) value and the Hill coefficient (n).

$$RV_{rel} = 1 / (1 + (GI_{50} / c_{inhibitor})^n) \qquad (S2)$$

Note: Reporter **2** were not toxic for 24 h at 10 µM.[S3] Also in this study, the count value of living cells with addition of reporter **2**, Hoechst 33342 and PI is always similar to that of living cells with only addition of Hoechst 33342 and PI.

4.4.1. Screening with Disulfides as Inhibitors

4.4.1.1. Screening with ETP as Inhibitors

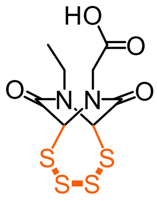

3

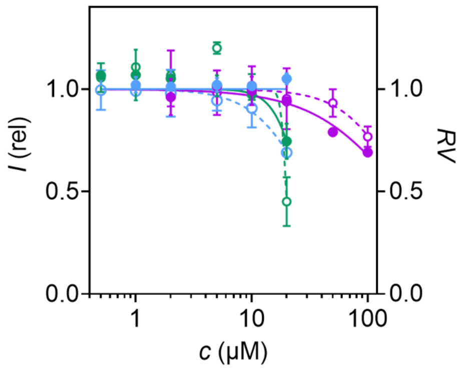

Fig. S35 Automatically analyzed HCHT data showing fluorescence intensity (filled symbols) and relative viability (empty symbols) of HeLa cells after pre-incubation with **3** for 1 h (blue circles), and 6 h with FBS (purple circles) followed by incubation with **2** (10 µM) for 30 min,

and incubation with **3** for 30 min (green circles) followed by co-incubation with **2** (10 µM) for 30 min.

Table S29 Inhibition of cellular uptake of **2** by **3** and cytotoxicity of **3** as a function of assay conditions

Entry	t_{pre}[a] (h)	t_{inc}[b] (h)	t_{co}[c] (h)	MIC[d] (µM)	IC_{50}[e] (µM)	n (IC_{50})[f]	GI_{50}[g] (µM)	n (GI_{50})[h]
1	1	0.5	-	> 20	> 20	-	> 20	-
2	6[i]	0.5	-	40	> 100	-	> 100	-
3	0.5	-	0.5	17	> 20	-	~ 20	-

[a]Pre-incubation time of HeLa cells with **3**. [b]Incubation time with **2** after pre-incubation[a] and removal of **3**. [c]Co-incubation time with **3** and **2** after pre-incubation[a] with **3**. [d]Minimum inhibitory concentration of **3**. [e]Half maximal inhibitory concentration of **3**. [f]Hill coefficient for inhibition of cellular uptake. [g]Concentration of **3** causing 50% cell growth inhibition. [h]Hill coefficient for toxicity. [i]Incubation with FBS.

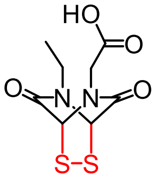

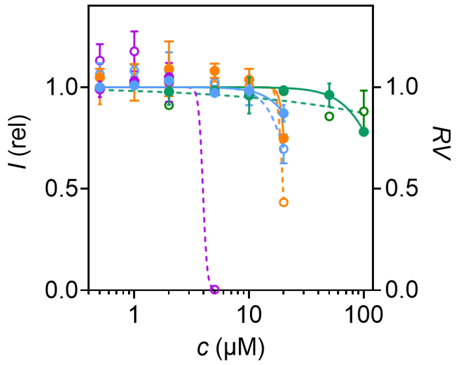

Fig. S36 Automatically analyzed HCHT data showing fluorescence intensity (filled symbols) and relative viability (empty symbols) of HeLa cells after pre-incubation with **4** for 1 h (blue circles), 4 h (purple circles) and 6 h with FBS (green circles) followed by incubation with **2** (10 μM) for 30 min, and incubation with **4** for 30 min (yellow circles) followed by co-incubation with **2** (10 μM) for 30 min.

Table S30 Inhibition of cellular uptake of **2** by **4** and cytotoxicity of **4** as a function of assay conditions

Entry	t_{pre}[a] (h)	t_{inc}[b] (h)	t_{co}[c] (h)	MIC[d] (μM)	IC_{50}[e] (μM)	n (IC_{50})[f]	GI_{50}[g] (μM)	n (GI_{50})[h]
1	1	0.5	-	> 20	> 20	-	> 20	-
2	4	0.5	-	> 2	> 2	-	~ 4	-
3	6[i]	0.5	-	80	> 100	-	> 100	-
4	0.5	-	0.5	20	> 20	-	~ 20	-

[a]Pre-incubation time of HeLa cells with **4**. [b]Incubation time with **2** after pre-incubation[a] and removal of **4**. [c]Co-incubation time with **4** and **2** after pre-incubation[a] with **4**. [d]Minimum

inhibitory concentration of **4**. [e]Half maximal inhibitory concentration of **4**. [f]Hill coefficient for inhibition of cellular uptake. [g]Concentration of **4** causing 50% cell growth inhibition. [h]Hill coefficient for toxicity. [i]Incubation with FBS.

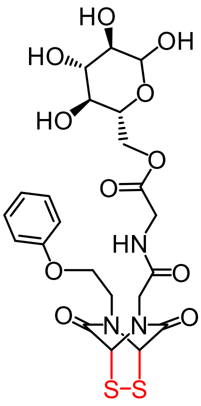

7

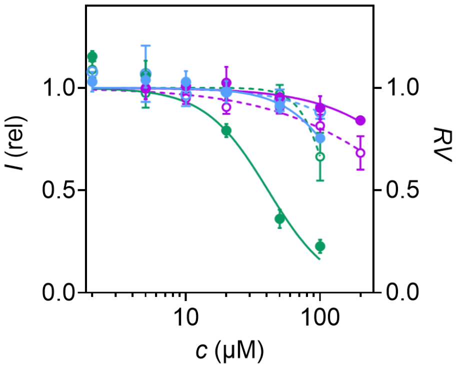

Fig. S37 Automatically analyzed HCHT data showing fluorescence intensity (filled symbols) and relative viability (empty symbols) of HeLa cells after pre-incubation with **7** for 4 h (blue circles) and 6 h with FBS (purple circles) followed by incubation with **2** (10 μM) for 30 min, and incubation with **7** for 23.5 h with FBS (green circles) followed by co-incubation with **2** (10 μM) for 30 min.

Table S31 Inhibition of cellular uptake of **2** by **7** and cytotoxicity of **7** as a function of assay conditions

Entry	t_{pre}[a] (h)	t_{inc}[b] (h)	t_{co}[c] (h)	MIC[d] (μM)	IC_{50}[e] (μM)	n (IC_{50})[f]	GI_{50}[g] (μM)	n (GI_{50})[h]
1	4	0.5	-	70	> 100	-	> 100	-
2	6[i]	0.5	-	180	> 200	-	> 200	-

4	23.5[i]	-	0.5	15	41 ± 4	-1.8 ± 0.4	> 100	-

[a]Pre-incubation time of HeLa cells with **7**. [b]Incubation time with **2** after pre-incubation[a] and removal of **7**. [c]Co-incubation time with **7** and **2** after pre-incubation[a] with **7**. [d]Minimum inhibitory concentration of **7**. [e]Half maximal inhibitory concentration of **7**. [f]Hill coefficient for inhibition of cellular uptake. [g]Concentration of **7** causing 50% cell growth inhibition. [h]Hill coefficient for toxicity. [i]Incubation with FBS.

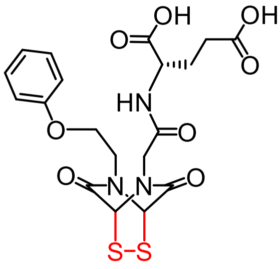

9

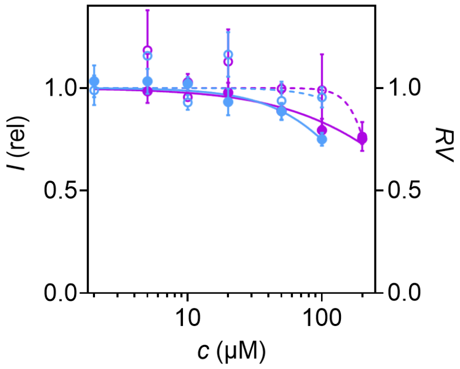

Fig. S38 Automatically analyzed HCHT data showing fluorescence intensity (filled symbols) and relative viability (empty symbols) of HeLa cells after pre-incubation with **9** for 4 h (blue circles) and 6 h with FBS (purple circles) followed by incubation with **2** (10 μM) for 30 min.

Table S32 Inhibition of cellular uptake of **2** by **9** and cytotoxicity of **9** as a function of assay conditions

Entry	t_{pre}[a] (h)	t_{inc}[b] (h)	MIC[c] (μM)	IC_{50}[d] (μM)	n (IC_{50})[e]	GI_{50}[f] (μM)	n (GI_{50})[g]
1	4	0.5	60	> 100	-	> 100	-
2	6[h]	0.5	80	> 200	-	> 200	-

[a]Pre-incubation time of HeLa cells with **9**. [b]Incubation time with **2** after pre-incubation[a] and removal of **9**. [c]Minimum inhibitory concentration of **9**. [d]Half maximal inhibitory concentration of **9**. [e]Hill coefficient for inhibition of cellular uptake. [f]Concentration of **9** causing 50% cell growth inhibition. [g]Hill coefficient for toxicity. [h]Incubation with FBS.

4.4.1.2. Screening with γ-Turn Peptides as Inhibitors

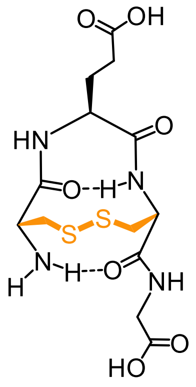

18

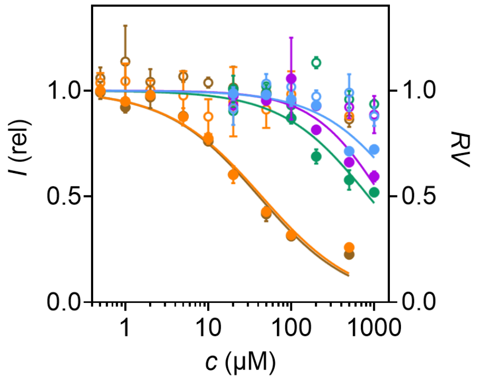

Fig. S39 Automatically analyzed HCHT data showing fluorescence intensity (filled symbols) and relative viability (empty symbols) of HeLa cells after pre-incubation with **18** for 1 h (blue circles), 2 h (purple circles) and 4 h (green circles) followed by incubation with **2** (10 µM) for 30 min, and incubation with **18** for 30 min (yellow circles) and 1.5 h (brown circles) followed by co-incubation with **2** (10 µM) for 30 min.

Table S33 Inhibition of cellular uptake of **2** by **18** and cytotoxicity of **18** as a function of assay conditions

Entry	t_{pre}[a] (h)	t_{inc}[b] (h)	t_{co}[c] (h)	MIC[d] (μM)	IC_{50}[e] (μM)	n (IC_{50})[f]	GI_{50}[g] (μM)	n (GI_{50})[h]
1	1	0.5	-	300	> 1000	-	> 1000	-
2	2	0.5	-	200	> 1000	-	> 1000	-
3	4	0.5	-	100	859 ± 140	-0.83 ± 0.12	> 1000	-
4	0.5	-	0.5	4	44 ± 5	-0.76 ± 0.06	> 500	-
5	1.5	-	0.5	4	41 ± 4	-0.78 ± 0.06	> 500	-

[a]Pre-incubation time of HeLa cells with **18**. [b]Incubation time with **2** after pre-incubation[a] and removal of **18**. [c]Co-incubation time with **18** and **2** after pre-incubation[a] with **18**. [d]Minimum inhibitory concentration of **18**. [e]Half maximal inhibitory concentration of **18**. [f]Hill coefficient for inhibition of cellular uptake. [g]Concentration of **18** causing 50% cell growth inhibition. [h]Hill coefficient for toxicity.

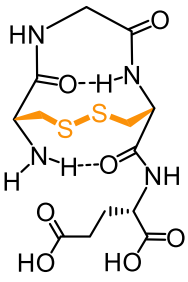

19

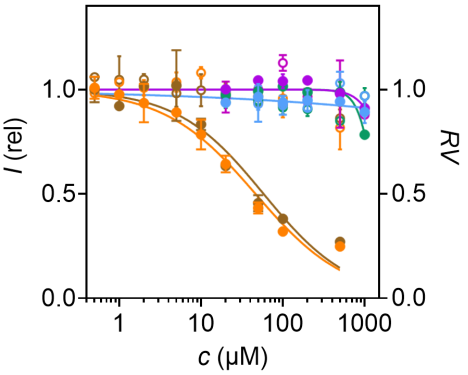

Fig. S40 Automatically analyzed HCHT data showing fluorescence intensity (filled symbols) and relative viability (empty symbols) of HeLa cells after pre-incubation with **19** for 1 h (blue

circles), 2 h (purple circles) and 4 h (green circles) followed by incubation with **2** (10 μM) for 30 min, and incubation with **19** for 30 min (yellow circles) and 1.5 h (brown circles) followed by co-incubation with **2** (10 μM) for 30 min.

Table S34 Inhibition of cellular uptake of **2** by **19** and cytotoxicity of **19** as a function of assay conditions

Entry	t_{pre}[a] (h)	t_{inc}[b] (h)	t_{co}[c] (h)	MIC[d] (μM)	IC_{50}[e] (μM)	n (IC_{50})[f]	GI_{50}[g] (μM)	n (GI_{50})[h]
1	1	0.5	-	> 1000	> 1000	-	> 1000	-
2	2	0.5	-	> 1000	> 1000	-	> 1000	-
3	4	0.5	-	900	> 1000	-	> 1000	-
4	0.5	-	0.5	5	46 ± 5	-0.78 ± 0.07	> 500	-
5	1.5	-	0.5	7	58 ± 10	-0.82 ± 0.11	> 500	-

[a]Pre-incubation time of HeLa cells with **19**. [b]Incubation time with **2** after pre-incubation[a] and removal of **19**. [c]Co-incubation time with **19** and **2** after pre-incubation[a] with **19**. [d]Minimum inhibitory concentration of **19**. [e]Half maximal inhibitory concentration of **19**. [f]Hill coefficient for inhibition of cellular uptake. [g]Concentration of **19** causing 50% cell growth inhibition. [h]Hill coefficient for toxicity.

4.4.2. Screening with BPS as Inhibitors

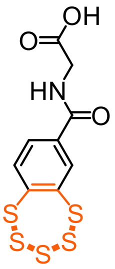

6

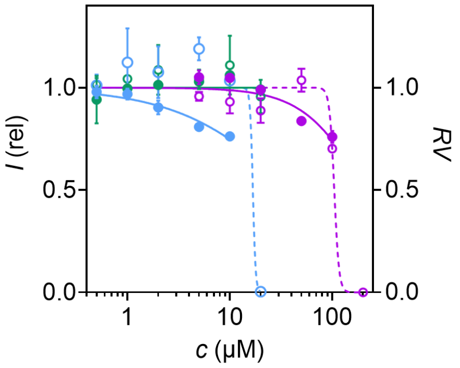

Fig. S41 Automatically analyzed HCHT data showing fluorescence intensity (filled symbols) and relative viability (empty symbols) of HeLa cells after pre-incubation with **6** for 4 h (blue circles) and 6 h with FBS (purple circles) followed by incubation with **2** (10 μM) for 30 min, and incubation with **6** for 30 min (green circles) followed by co-incubation with **2** (10 μM) for 30 min.

Table S35 Inhibition of cellular uptake of **2** by **6** and cytotoxicity of **6** as a function of assay conditions

Entry	t_{pre}^{a} (h)	t_{inc}^{b} (h)	t_{co}^{c} (h)	MIC^{d} (μM)	IC$_{50}^{e}$ (μM)	n (IC$_{50}$)^{f}	GI$_{50}^{g}$ (μM)	n (GI$_{50}$)^{h}
1	4	0.5	-	4	> 10	-	~ 17	-
2	6^{i}	0.5	-	60	> 100	-	~ 105	-
3	0.5	-	0.5	> 20	> 20	-	> 20	-

[a]Pre-incubation time of HeLa cells with **6**. [b]Incubation time with **2** after pre-incubation[a] and removal of **6**. [c]Co-incubation time with **6** and **2** after pre-incubation[a] with **6**. [d]Minimum inhibitory concentration of **6**. [e]Half maximal inhibitory concentration of **6**. [f]Hill coefficient for

inhibition of cellular uptake. [g]Concentration of **6** causing 50% cell growth inhibition. [h]Hill coefficient for toxicity. [i]Incubation with FBS.

4.4.3. Screening with Thiosulfonates as Inhibitors

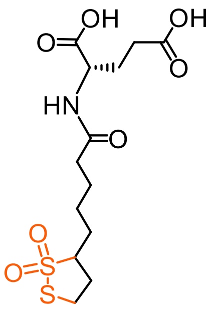

15

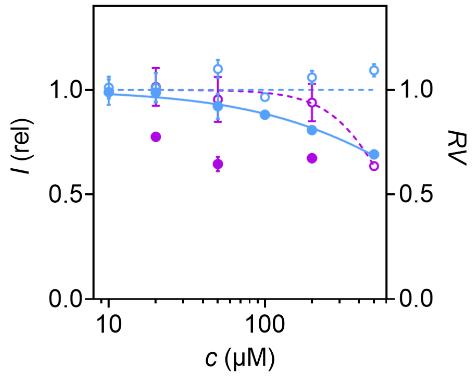

Fig. S42 Automatically analyzed HCHT data showing fluorescence intensity (filled symbols) and relative viability (empty symbols) of HeLa cells after pre-incubation with **15** for 4 h (blue circles) followed by incubation with **2** (10 μM) for 30 min, and incubation with **15** for 23.5 h with FBS (purple circles) followed by co-incubation with **2** (10 μM) for 30 min.

Table S36 Inhibition of cellular uptake of **2** by **15** and cytotoxicity of **15** as a function of assay conditions

Entry	t_{pre}[a] (h)	t_{inc}[b] (h)	t_{co}[c] (h)	MIC[d] (μM)	IC_{50}[e] (μM)	n (IC_{50})[f]	GI_{50}[g] (μM)	n (GI_{50})[h]
1	4	0.5	-	150	> 500	-	> 500	-
2	23.5[i]	-	0.5	< 20	> 500	-	> 500	-

[a]Pre-incubation time of HeLa cells with **15**. [b]Incubation time with **2** after pre-incubation[a] and removal of **15**. [c]Co-incubation time with **15** and **2** after pre-incubation[a] with **15**. [d]Minimum inhibitory concentration of **15**. [e]Half maximal inhibitory concentration of **15**. [f]Hill coefficient for inhibition of cellular uptake. [g]Concentration of **15** causing 50% cell growth inhibition. [h]Hill coefficient for toxicity. [i]Incubation with FBS.

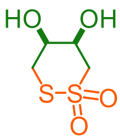

16

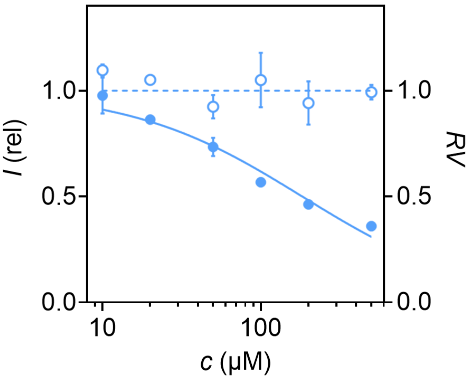

Fig. S43 Automatically analyzed HCHT data showing fluorescence intensity (filled symbols) and relative viability (empty symbols) of HeLa cells after pre-incubation with **16** for 4 h (blue circles) followed by incubation with **2** (10 μM) for 30 min.

Table S37 Inhibition of cellular uptake of **2** by **16** and cytotoxicity of **16** as a function of assay conditions

Entry	t_{pre}[a] (h)	t_{inc}[b] (h)	MIC[c] (μM)	IC_{50}[d] (μM)	n (IC_{50})[e]	GI_{50}[f] (μM)	n (GI_{50})[g]
1	4	0.5	20	183 ± 18	-0.80 ± 0.07	> 500	-

[a]Pre-incubation time of HeLa cells with **16**. [b]Incubation time with **2** after pre-incubation[a] and removal of **16**. [c]Minimum inhibitory concentration of **16**. [d]Half maximal inhibitory

concentration of **16**. [e]Hill coefficient for inhibition of cellular uptake. [f]Concentration of **16** causing 50% cell growth inhibition. [g]Hill coefficient for toxicity.

5. Preliminary Tests on Antiviral Activity

Antiviral activity tests including inhibition of virus (SARS-CoV-2 and vesicular-somatitis virus) entry and cell viability measurement were performed by NEURIX (64, Avenue de la Roseraie 1205, Geneva, Switzerland). pCG1_SCoV-2 plasmid encoding SARS-CoV-2 S-protein was provided by Prof. Dr. Stefan Pöhlmann (Deutsches Primatenzentrum, Leibniz-Institute for Primate research, Göttingen, Germany).

Lentivirus generation:

Standard lentivirus was generated using transient transfection of HEK 293T/17 cells with the CD511B plasmid coding for green fluorescent protein and Gaussia luciferase under the control of cytomegalovirus (CMV) and elongation factor-1 (EF1) promoters, respectively, the psPAX2 plasmid encoding gag/pol, and the pCAG-VSVG envelope plasmid, as described in ref. 23. To generate the SARS-CoV-2 pseudo virus, the pCAG-VSVG plasmid was replaced by the pCG1 plasmid expressing the C-terminaly truncated SARS-CoV-2 spike protein harboring the D614G mutation under CMV promoter. Human lung epithelial A549 cells were cultivated in DMEM media supplemented with 10% fetal bovine serum and 1% non-essential amino-acid in humidified incubator at 37°C with 5% CO_2, all from Thermofisher scientific.

Compound treatment:

Stock solutions of 6 selected compounds (**3** (50 mM), **4** (20 mM), **6** (20 mM), **15** (400 mM), **16** (500 mM) and **18** (500 mM)) in DMSO were prepared freshly. Before cell experiment, these solutions were diluted 1000 time with culture media to give each desired solution (**3** (50

μM), **4** (20 μM), **6** (20 μM), **15** (400 μM), **16** (500 μM) and **18** (500 μM)) with only 0.1% of DMSO (avoid any off-target effect from DMSO).

A549 cells were treated with these compounds (desired solutions (1x) or 10 times diluted from these desired solutions (10x)) for 24 h or 1 h prior to addition of the lentiviruses. Six hours later the culture media containing both compounds and lentivirus was discarded and fresh culture media was added. 72 hours later, 10 μL of culture media was sampled and mixed with 50 μL of PBS containing 4 μM of Coelentrazine (Apollo Scientific) and luminescence generated by the gaussia luciferase reporter was measured. In parallel 10 μL of cell counting kit WST8 (Sigma Aldrich) was added on cells to measure viability by absorbance at 450 nm according to manufacturer instructions.

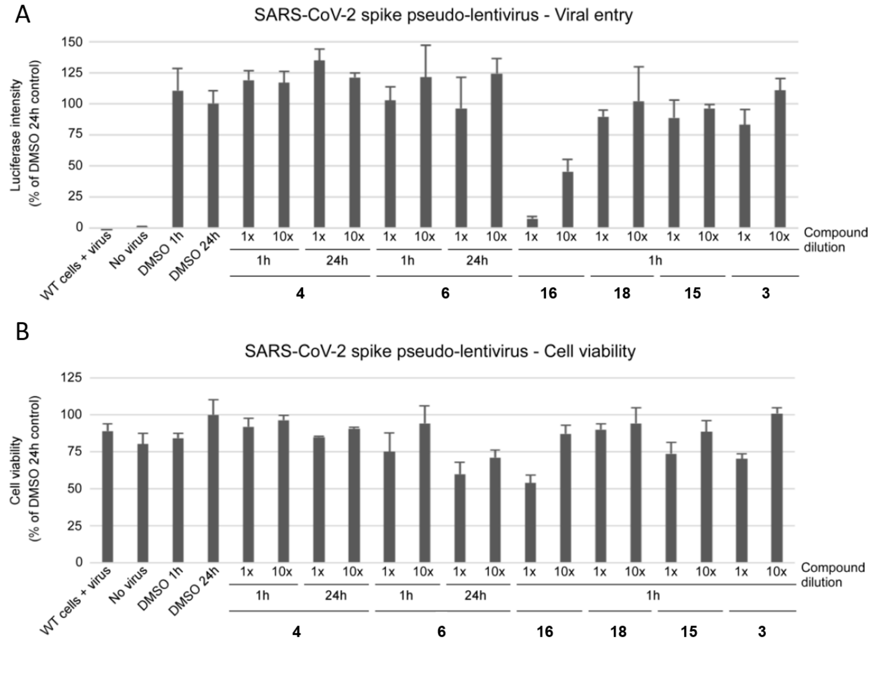

Fig. S44 Virus entry inhibition (A) and cell viability (B) results using A549 human lung alveolar basal epithelium cells overexpressing ACE2 and TMPRSS2 which transduced with lentivirus expressing the SARS-CoV-2 spike surface protein harboring the D614G mutation.

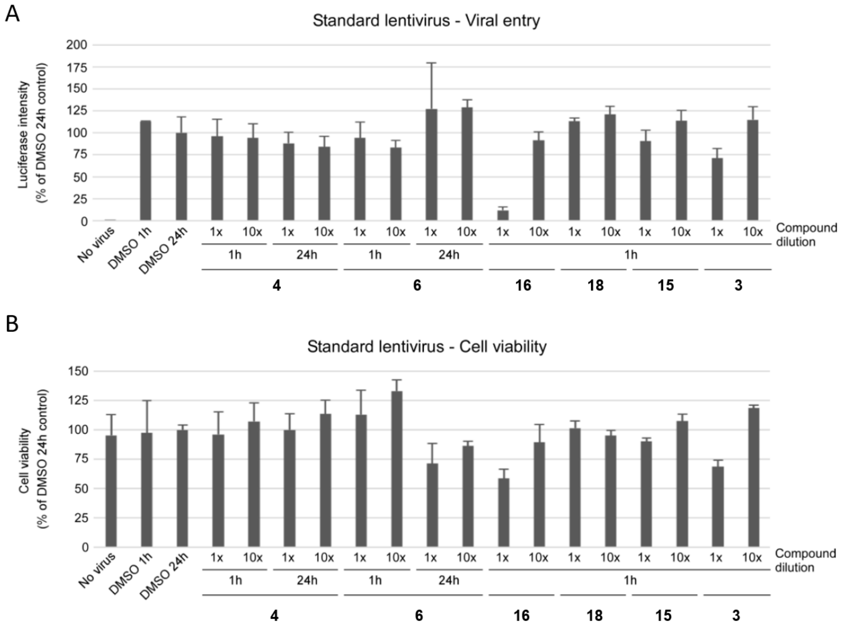

Fig. S45 Virus entry inhibition (A) and cell viability (B) results using wild type A549 cells transduced with a standard lentivirus which expressing VSVG.

6. Supplementary References

S1 J. López-Andarias, J. Saarbach, D. Moreau, Y. Cheng, E. Derivery, Q. Laurent, M. González-Gaitán, N. Winssinger, N. Sakai and S. Matile, *J. Am. Chem. Soc.*, 2020, **142**, 4784–4792.

S2 L. Zong, E. Bartolami, D. Abegg, A. Adibekian, N. Sakai and S. Matile, *ACS Cent. Sci.*, 2017, **3**, 449–453.

S3 Y. Cheng, L. Zong, J. López-Andarias, E. Bartolami, Y. Okamoto, T. R. Ward, N. Sakai and S. Matile, *Angew. Chem. Int. Ed.,* 2019, **58**, 9522–9526.

S4 E. Marchal, Md. I. Uddin, C. L. A. Hawco and A. Thompson, *Can. J. Chem.*, 2015, **93**, 526–535.

S5 Y. Cui, Z. Cheng, J. Mao and Y. Yu, *Tetrahedron Lett.*, 2013, **54**, 3831–3833.

S6 A. Saha, S. Panda, S. Paul and D. Manna, *Chem. Commun.*, 2016, **52**, 9438–9441.

S7 Y. Ma and C. Wu, *J. Mater. Chem. B*, 2014, **2**, 3282–3291.

S8 (*a*) I. Cacciatore, A. D. Stefano, S. Duprè, E. Morera, F. Pinnen and A. Spirito, *Bioorg. Chem.*, 2003, **31**, 109–121. (*b*) B. Schmidt, S. Lindman, W. Tong, G. Lindeberg, A. Gogoll, Z. Lai, M. Thörnwall, B. Synnergren, A. Nilsson, C. J. Welch, M. Sohtell, C. Westerlund, F. Nyberg, A. Karlén and A. Hallberg, *J. Med. Chem.*, 1997, **40**, 903–919. (*c*) R. Kishore and P. Balaram, *Biopolymers.*, 1985, **24**, 2041–2043.

S9 A. Tirla, M. Hansen and P. Rivera-Fuentes, *Synlett.*, 2018, **29**, 1289−1292.

S10 J. Hayashi, Y. Samezawa, Y. Ochi, S. I. Wada and H. Urata, *Bioorg. Med. Chem. Lett.*, 2017, **27**, 3135−3138.

S11 H. F. Motiwala, Y.-H. Kuo, B. L. Stinger, B. A. Palfey and B. R. Martin, *J. Am. Chem. Soc.*, 2020, **142**, 1801–1810.

S12 M. Söderström, E. Zamaratski and L. R. Odell, *Eur. J. Org. Chem.*, 2019, 5402–5408.

S13 N. Toda, S. Asano and C. F., 3rd, Barbas, *Angew. Chem. Int. Ed.*, 2013, **52**, 12592–12596.

S14 P. K. Singh, L. Field and B. J. Sweetman, *J. Org. Chem.*, 1988, **53**, 2608–2612.

S15 L. Field and Y. H. Khim, *J. Org. Chem.*, 1972, **37**, 2710–2714.

S16 D. P. Donnelly, M. G. Dowgiallo, J. P. Salisbury, K. C. Aluri, S. Iyengar, M. Chaudhari, M. Mathew, I. Miele, J. R. Auclair, S. A. Lopez, R. Manetsch and J. N. Agar, *J. Am. Chem. Soc.*, 2018, **140**, 7377–7380.

S17 G. Agnihotri, B. M. Crall, T. C. Lewis, T. P. Day, R. Balakrishna, H. J. Warshakoon, S. S. Malladi and S. A. David, *J. Med. Chem.*, 2011, **54**, 8148−8160.

S18 H. Richard, M. Antonio, T. Barry and W. Iain, *PCT Int. Appl.*, 2004, WO 2004108692 A1.

S19 Y. Du, L. Xia, A. Jo, R. M. Davis, P. Bissel, M. F. Ehrich and D. G. I. Kingston, *Bioconjugate Chem.*, 2018, **29**, 420–430.

S20 (*a*) B. K. Law and R. K. Castellano, *PCT Int. Appl.*, 2019, WO 2019241644 Al. (*b*) W. G. Rice, R. R. Schultz, D. C. Baker and L. E. Henderson, *PCT Int. Appl.*, 1998, WO 9801440 A2.

S21 Z. Wang, C. Zhang, H. Wang, Y. Xiong, X. Yang, Y.-e. Shi and A. L. Rogach, *Angew. Chem. Int. Ed.*, 2020, **59**, 9997–10002.

S22 A. Kamyshny, A. Goifman, J. Gun, D. Rizkov and O. Lev, *Environ. Sci. Technol.*, 2004, **38**, 6633–6644.

S23 M. Giry-Laterrière, E. Verhoeyen and P. Salmon, *Methods Mol. Biol.*, 2011, **737**, 183–209.

The original data can be found at: https://dx.doi.org/10.5281/zenodo.4109129

7. NMR Spectra

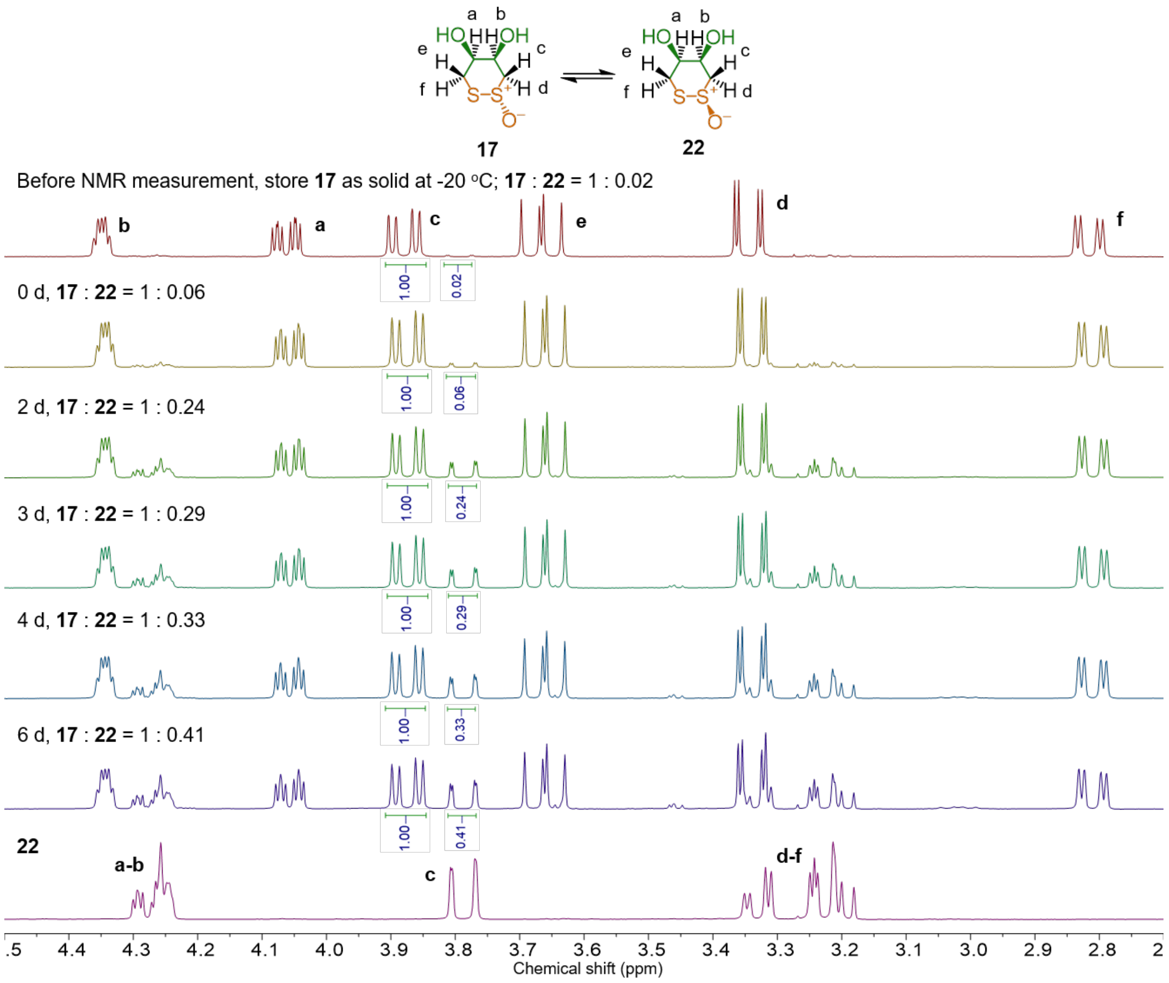

Fig. S46 ^{1}H NMR (400 MHz, D_2O) spectrum showing the isomerization of **17** to **22** with increasing of time at rt.

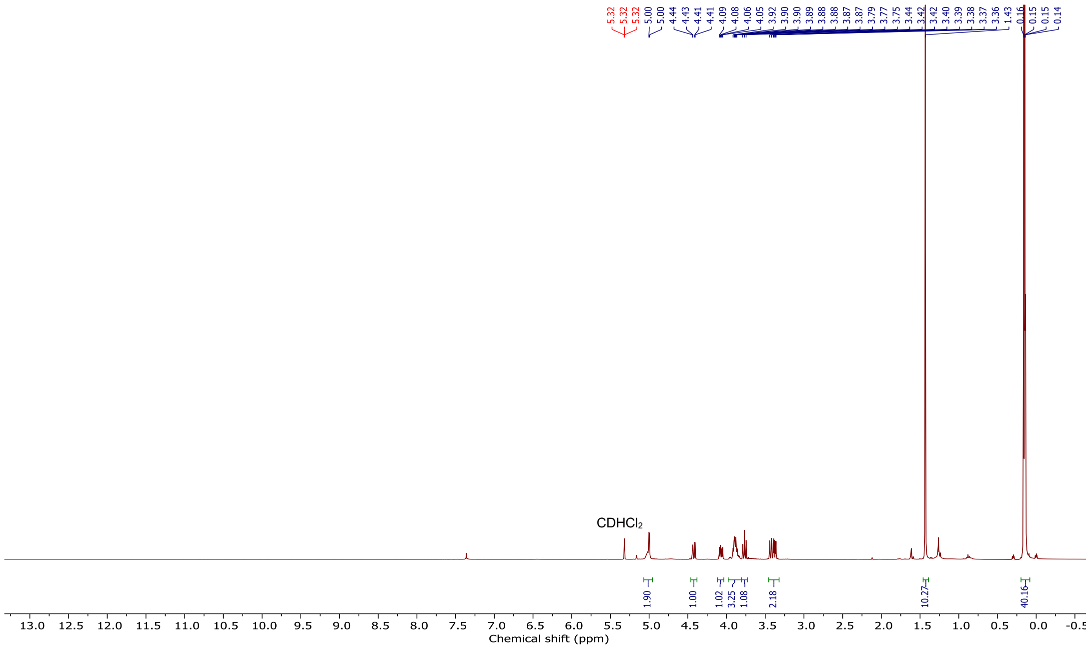

Fig. S47 ^{1}H NMR (400 MHz, CD_2Cl_2) spectrum of **34**.

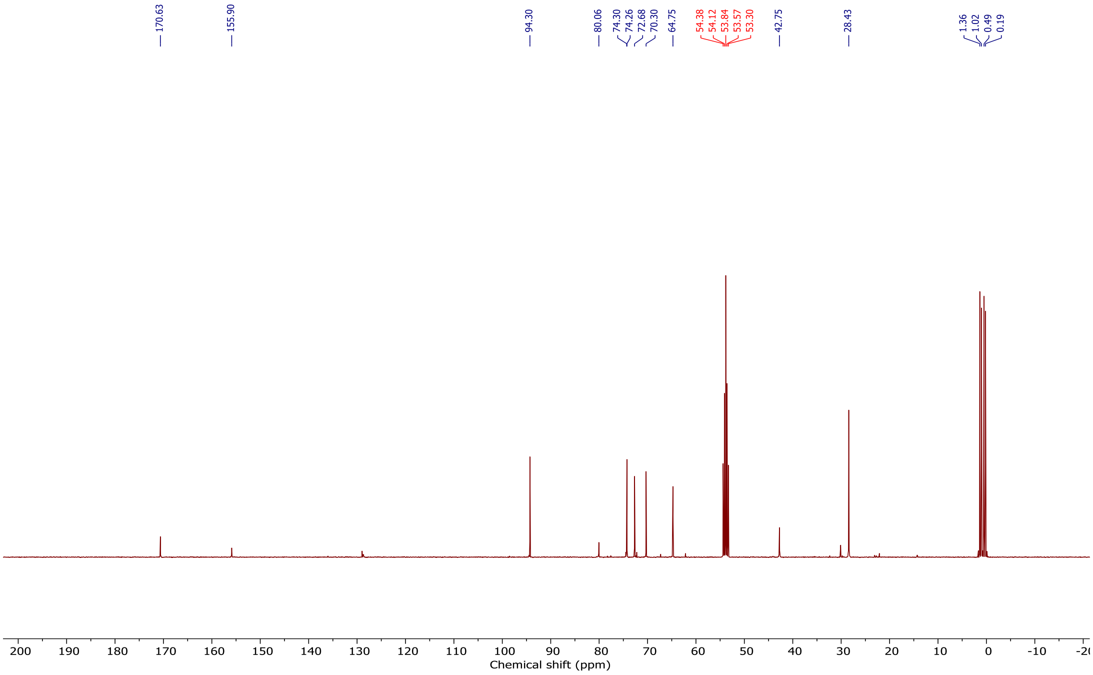

Fig. S48 ^{13}C NMR (100 MHz, CD_2Cl_2) spectrum of **34**.

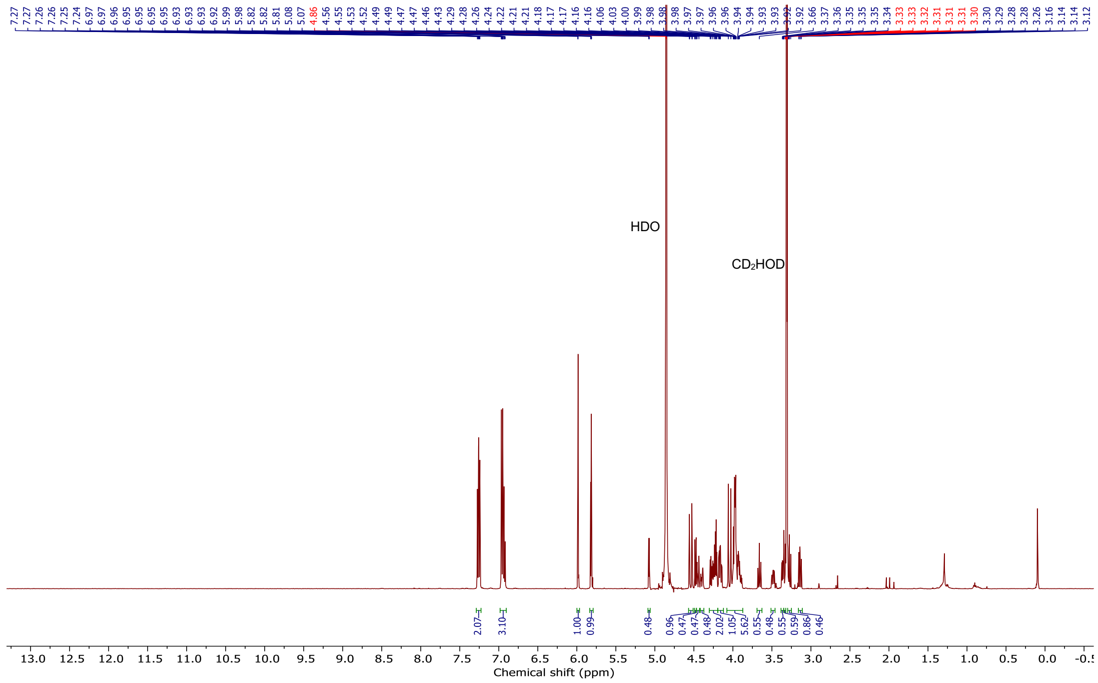

Fig. S49 ^{1}H NMR (400 MHz, CD_3OD) spectrum of **7**.

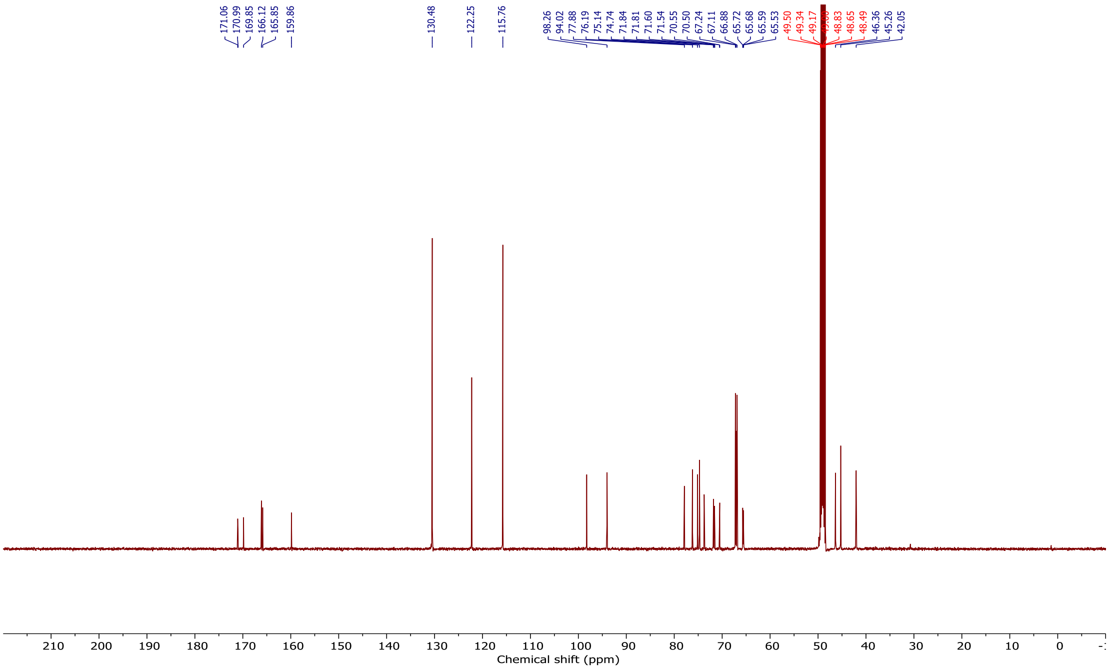

Fig. S50 ^{13}C NMR (125 MHz, CD_3OD) spectrum of **7**.

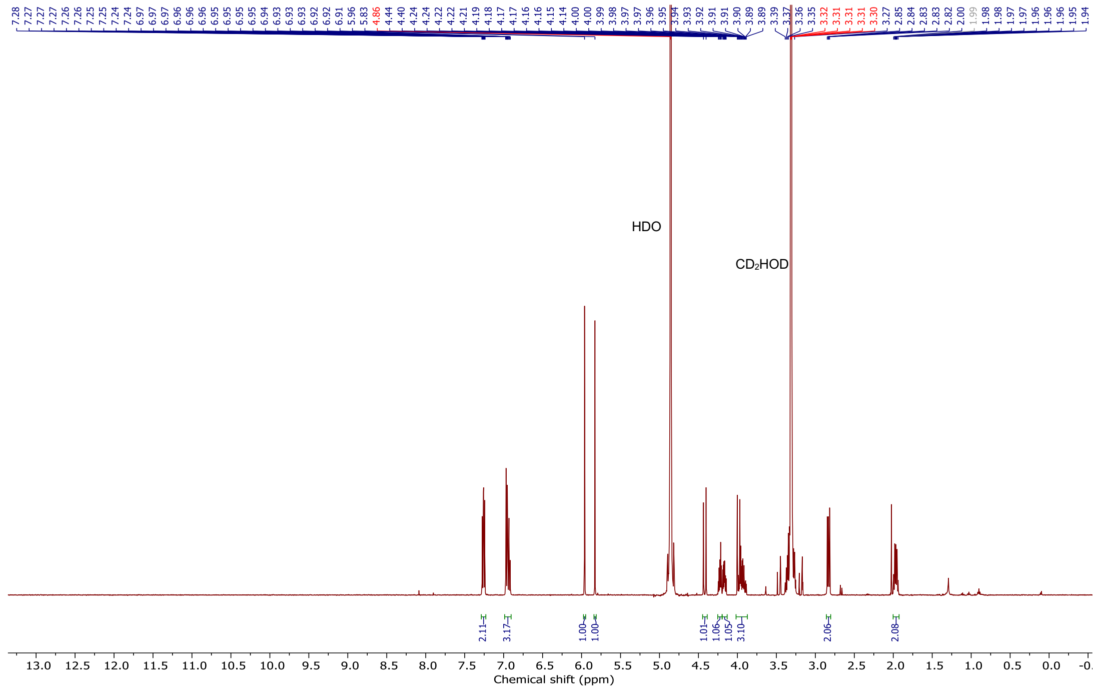

Fig. S51 ^{1}H NMR (500 MHz, CD_3OD) spectrum of **8**.

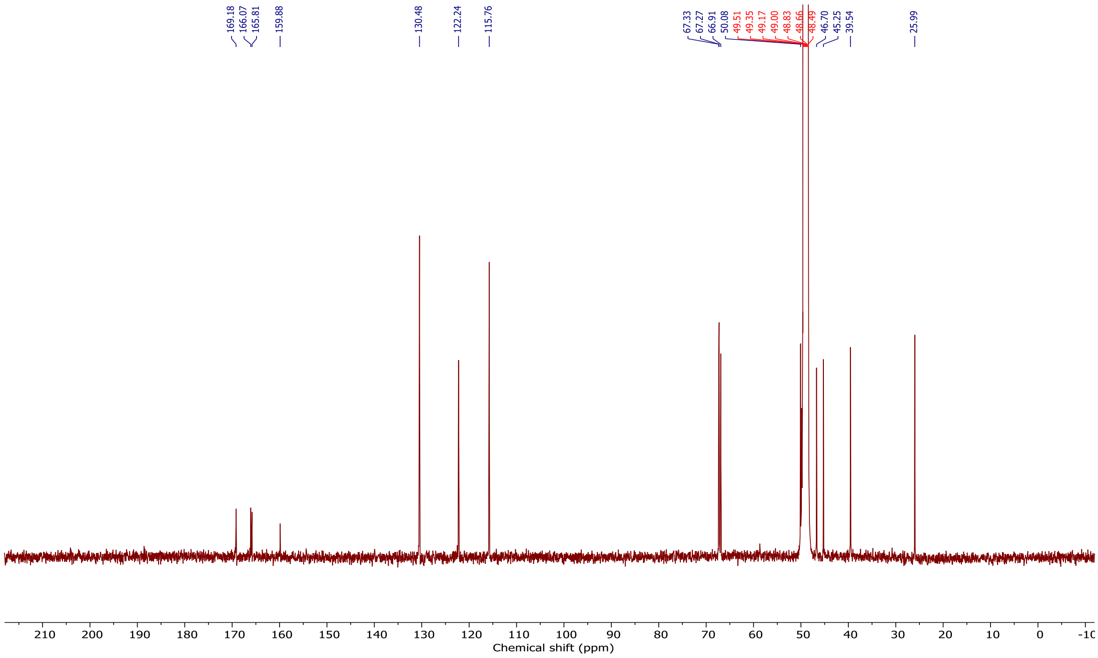

Fig. S52 ^{13}C NMR (125 MHz, CD_3OD) spectrum of **8**.

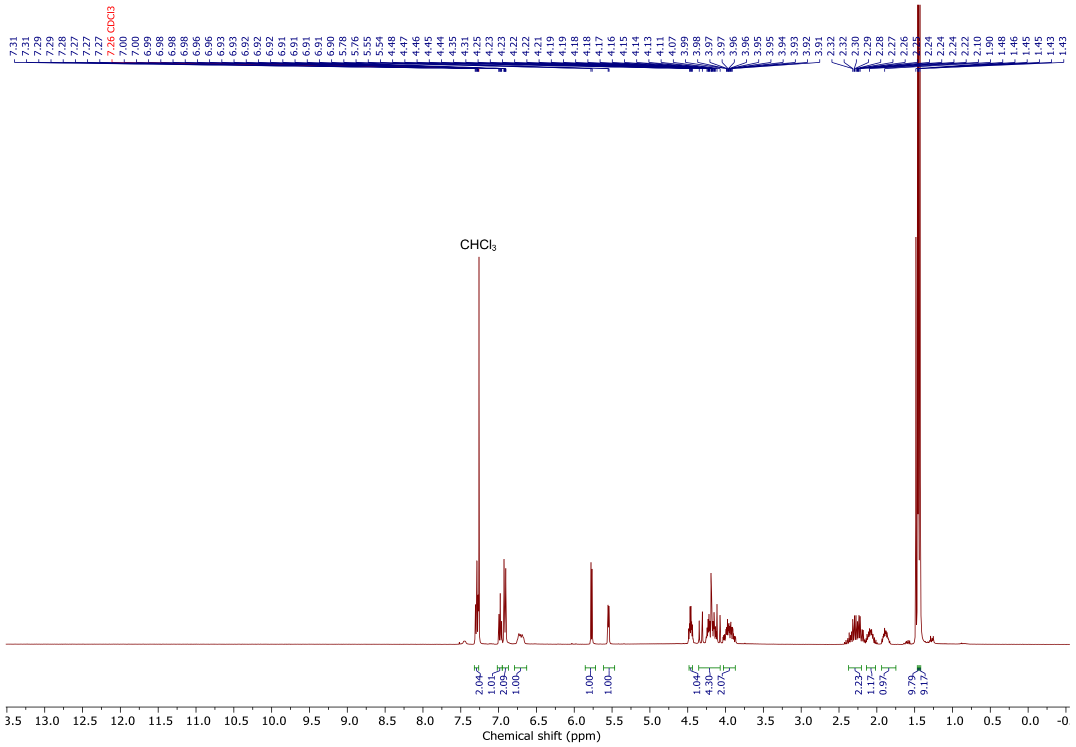

Fig. S53 ^{1}H NMR (400 MHz, $CDCl_3$) spectrum of **38** (mixture of 2 diastereomers).

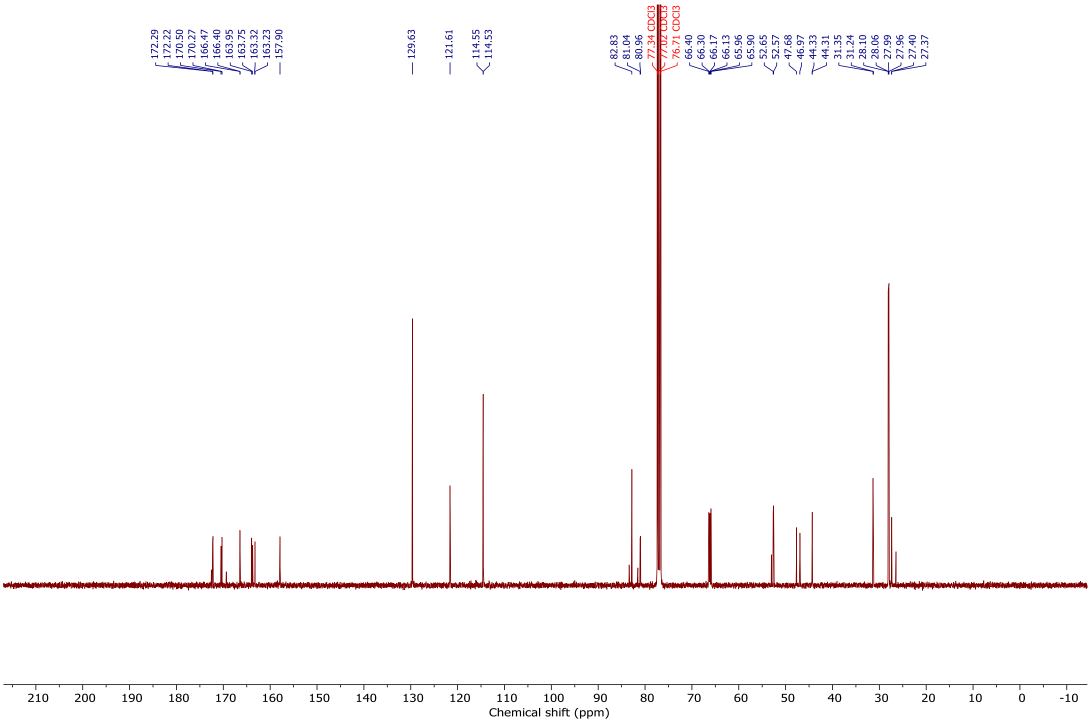

Fig. S54 ^{13}C NMR (100 MHz, $CDCl_3$) spectrum of **38** (mixture of 2 diastereomers).

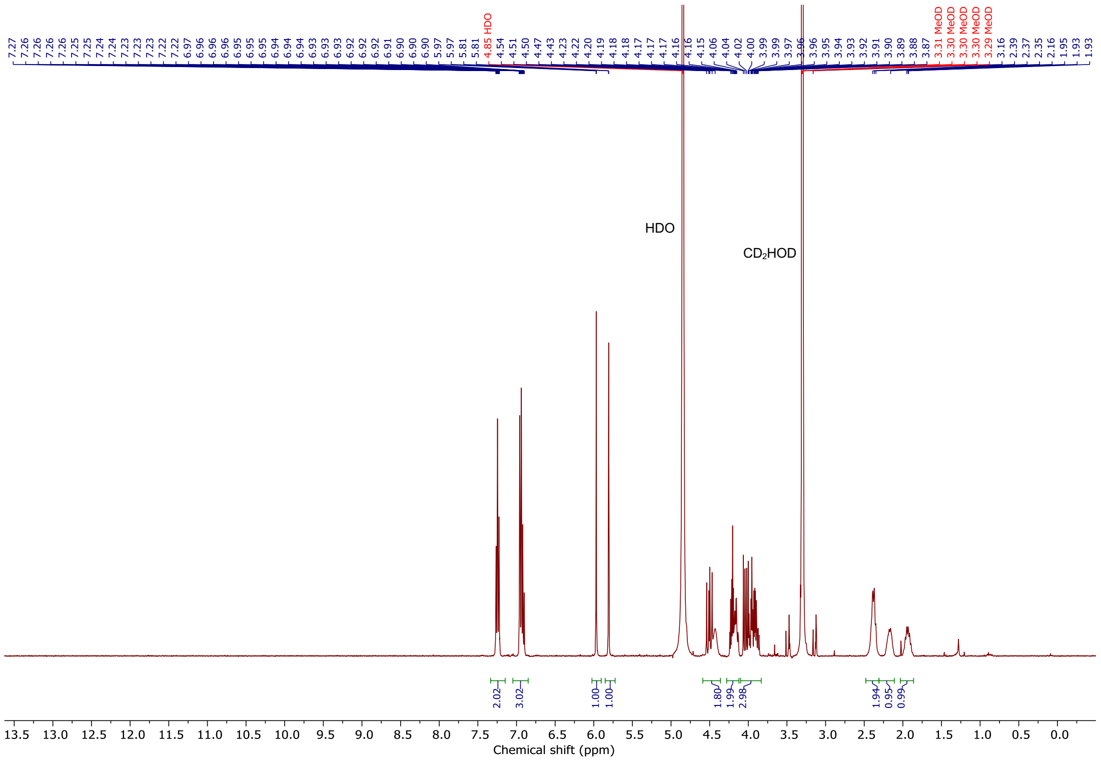

Fig. S55 ^{1}H NMR (400 MHz, CD_3OD) spectrum of **9** (mixture of 2 diastereomers).

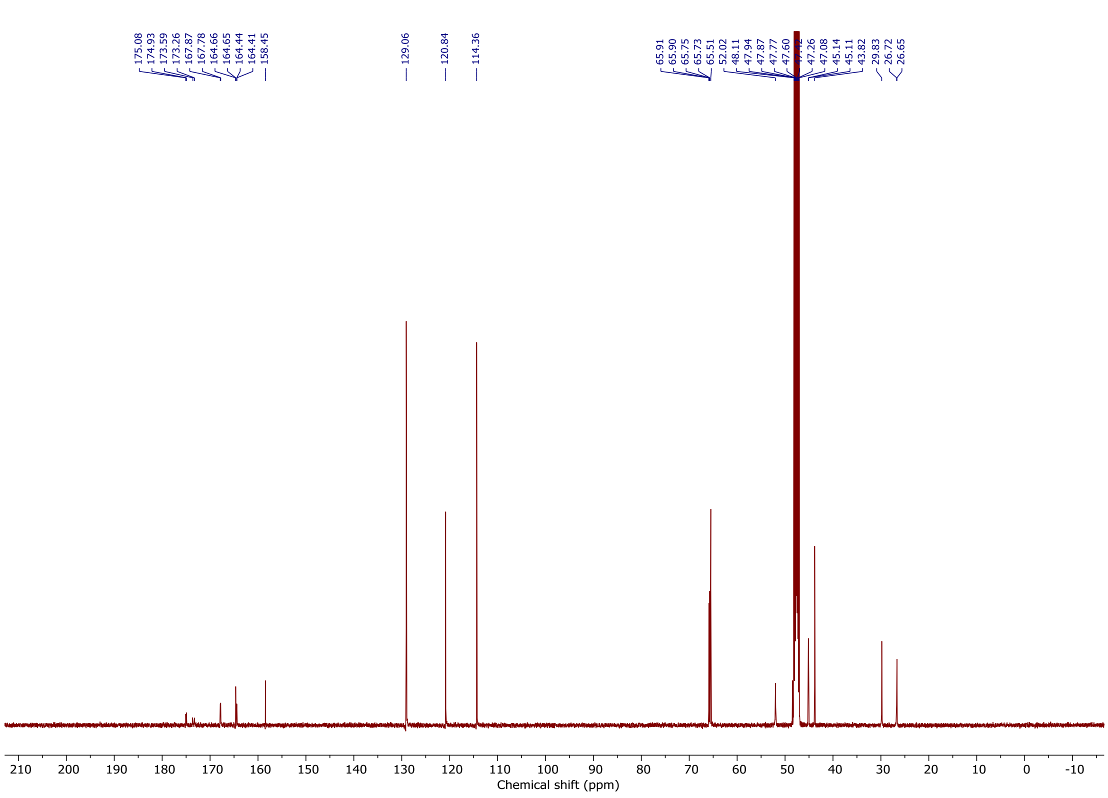

Fig. S56 ^{13}C NMR (100 MHz, CD_3OD) spectrum of **9** (mixture of 2 diastereomers).

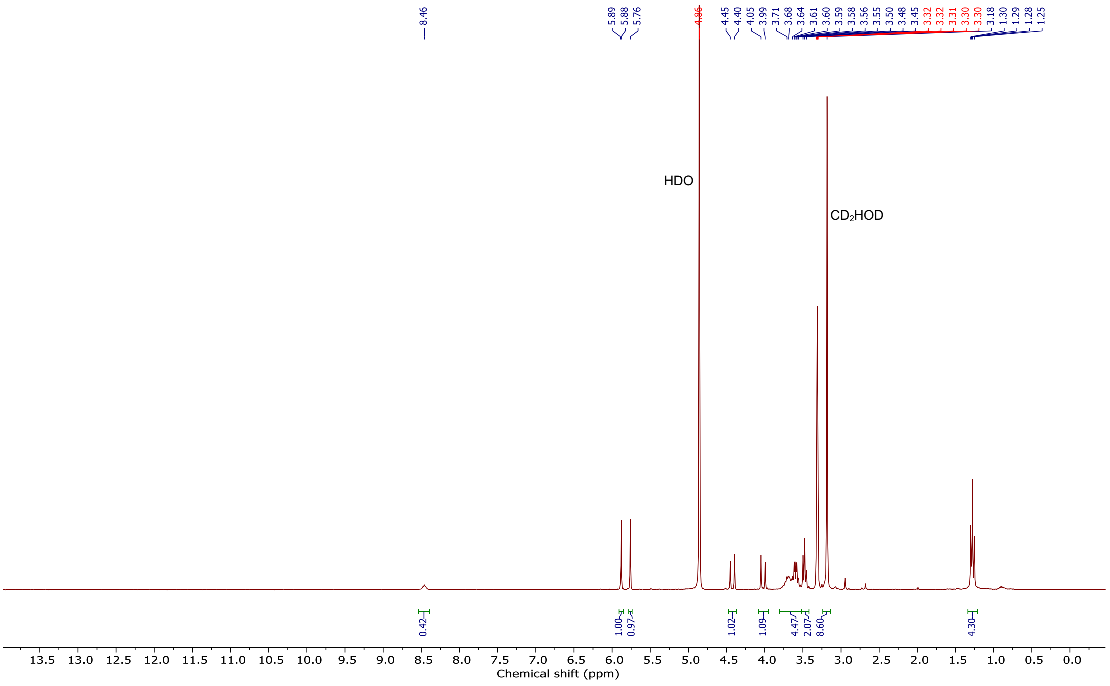

Fig. S57 ^{1}H NMR (400 MHz, CD_3OD) spectrum of **12.**

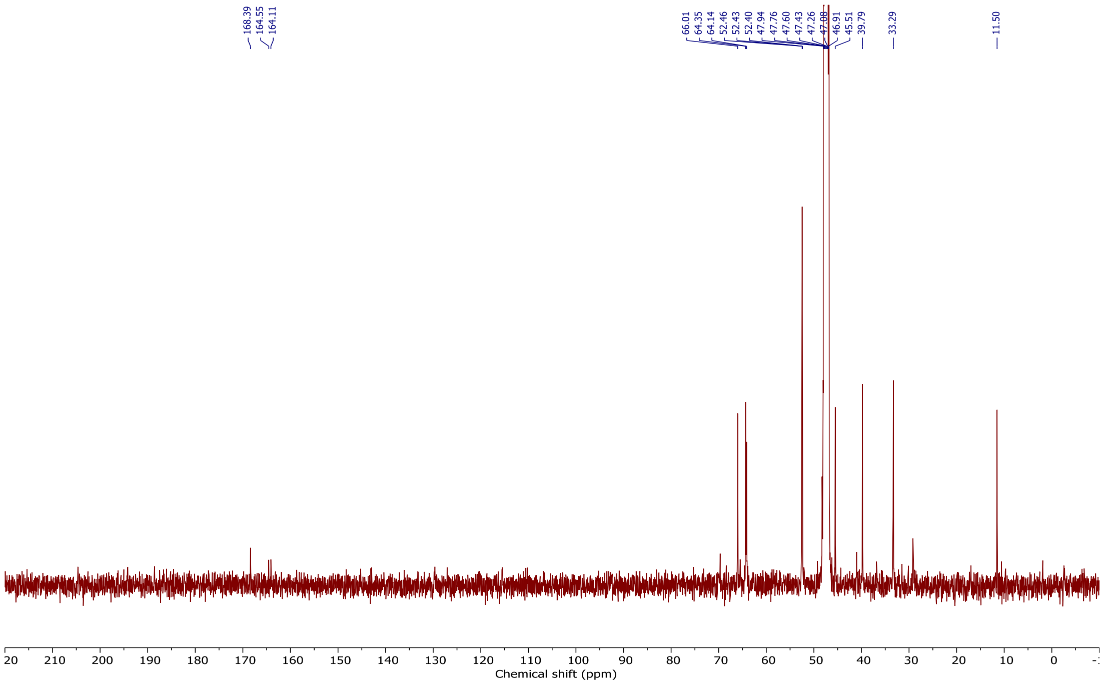

Fig. S58 ^{13}C NMR (100 MHz, CD_3OD) spectrum of **12.**

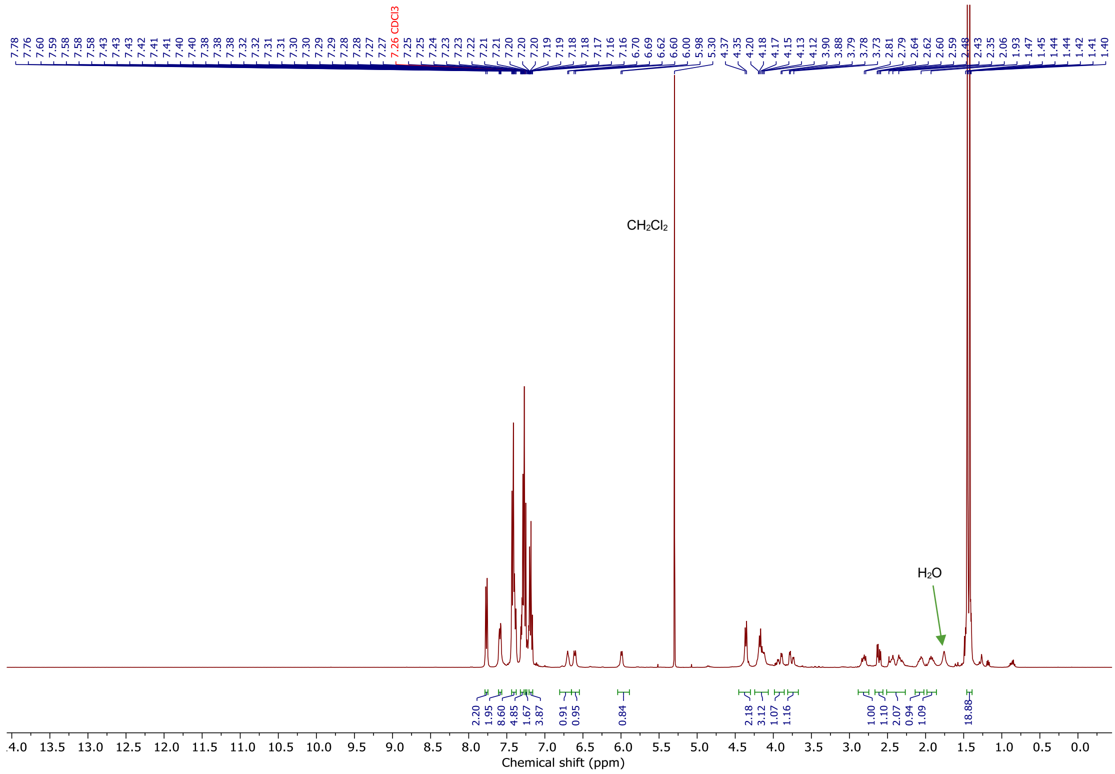

Fig. S59 ^{1}H NMR (400 MHz, $CDCl_3$) spectrum of **42.**

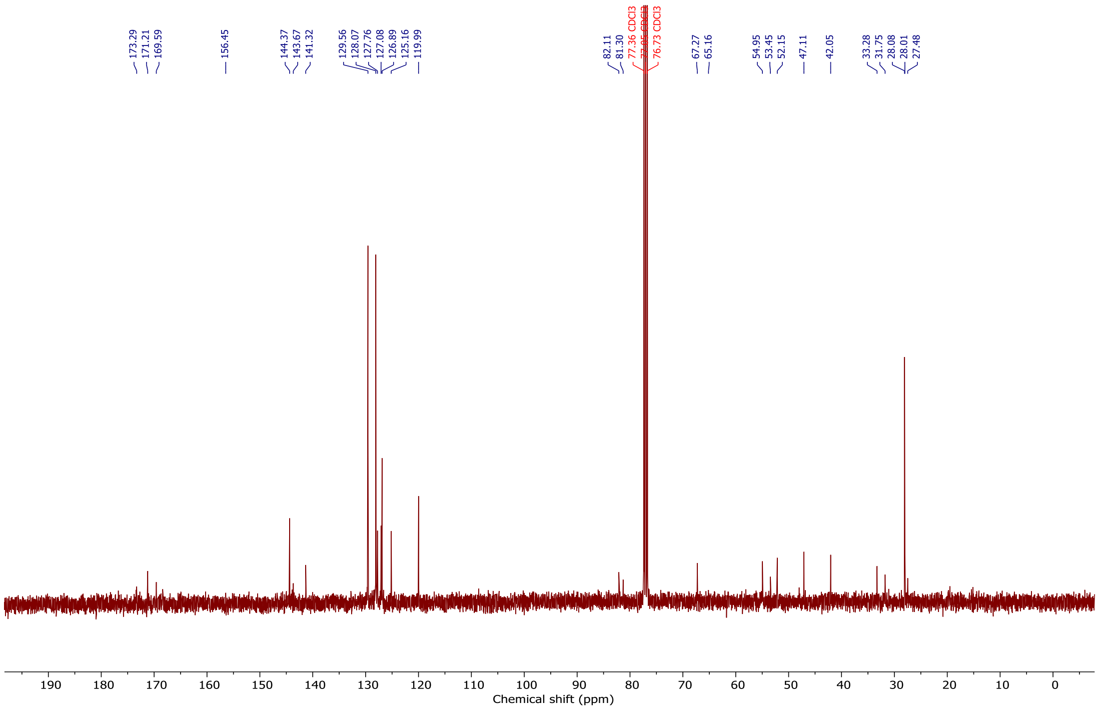

Fig. S60 ^{13}C NMR (100 MHz, $CDCl_3$) spectrum of **42.**

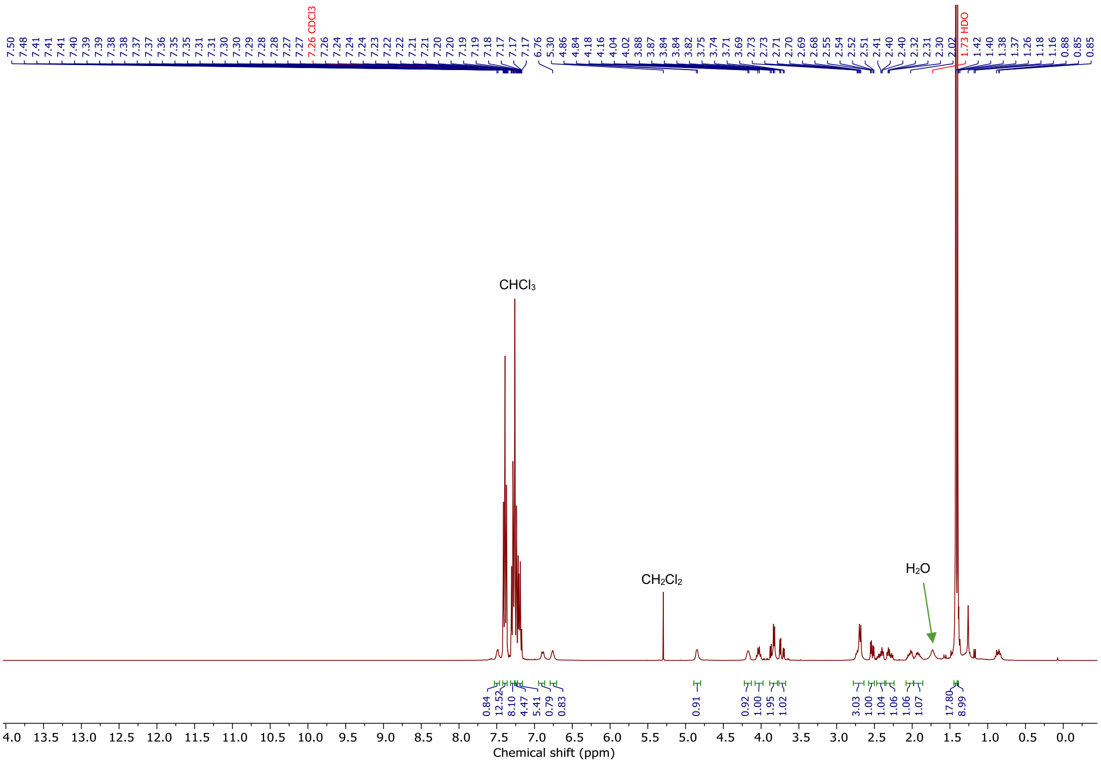

Fig. S61 ^{1}H NMR (400 MHz, $CDCl_3$) spectrum of **43.**

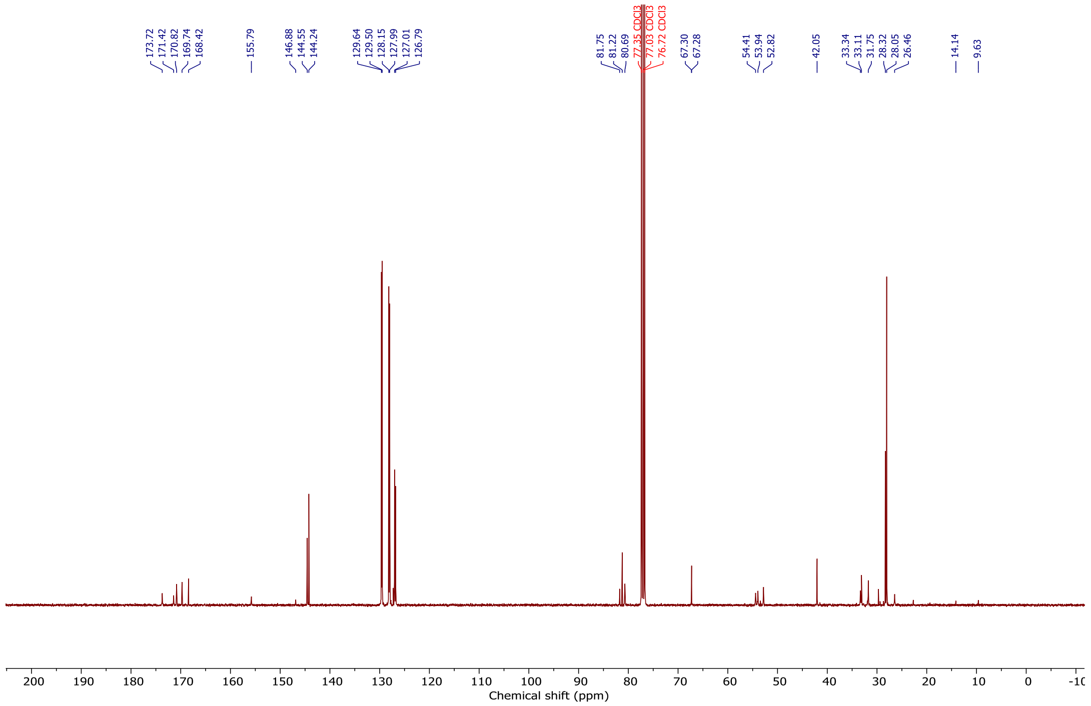

Fig. S62 ^{13}C NMR (125 MHz, $CDCl_3$) spectrum of **43.**

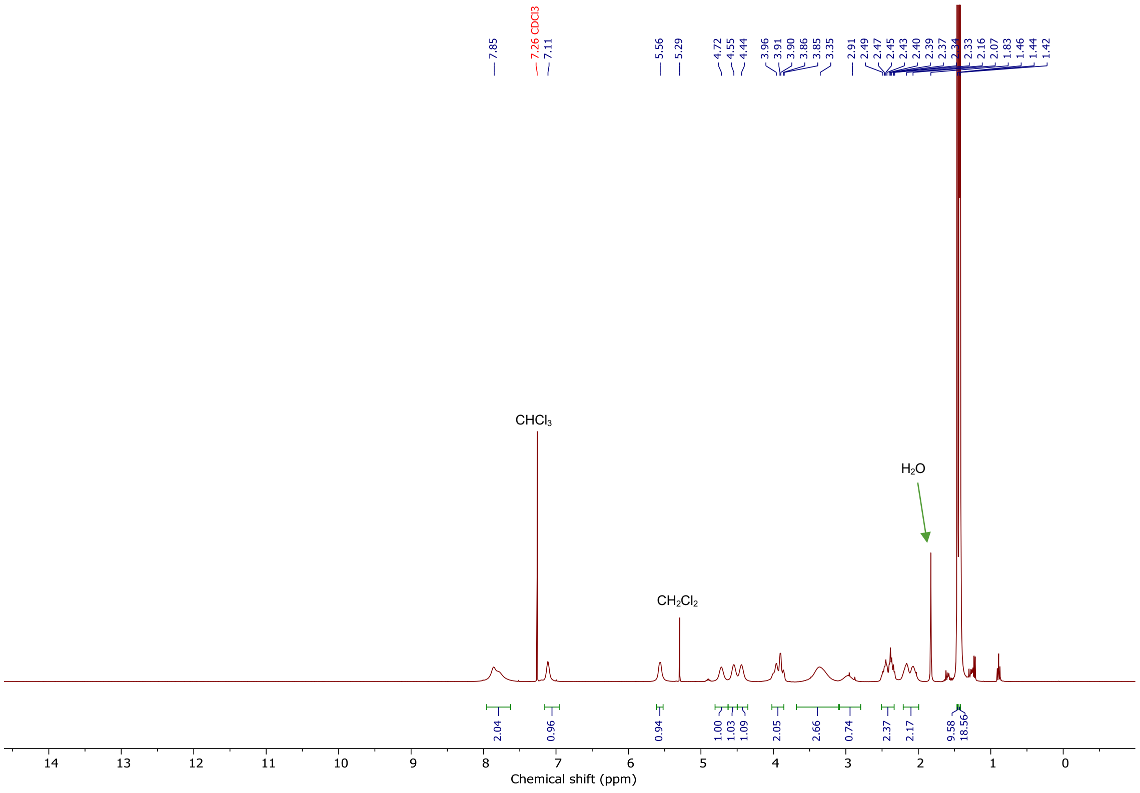

Fig. S63 ^{1}H NMR (400 MHz, $CDCl_3$) spectrum of **44.**

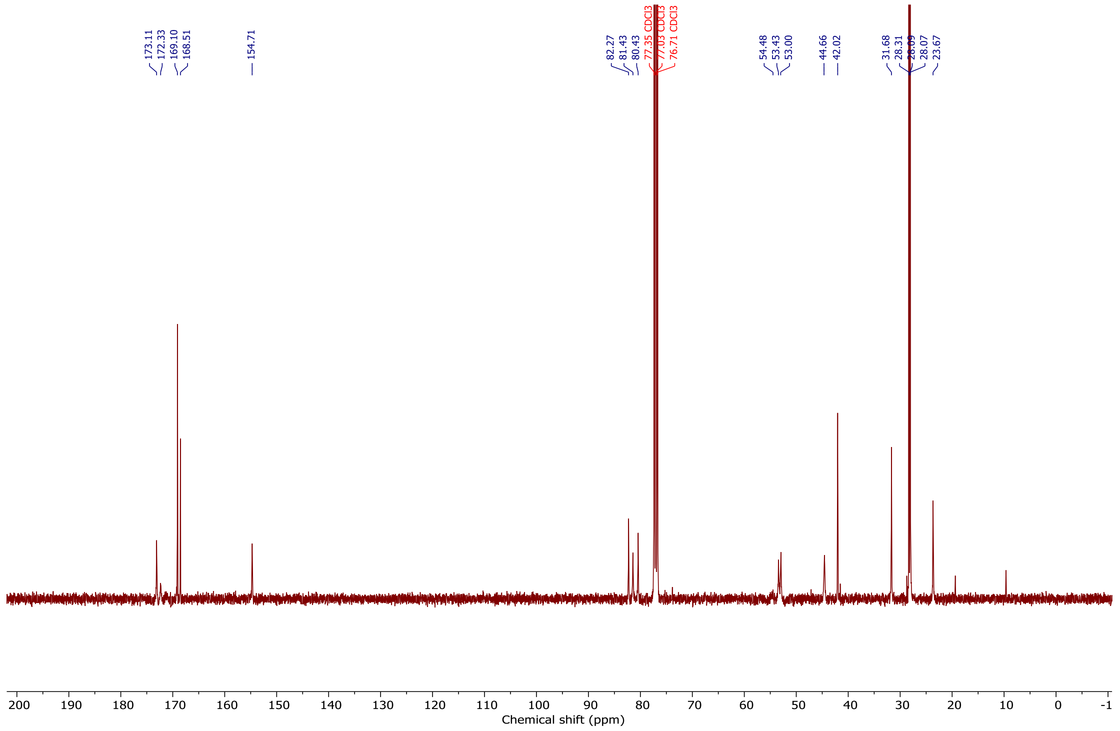

Fig. S64 ^{13}C NMR (125 MHz, $CDCl_3$) spectrum of **44.**

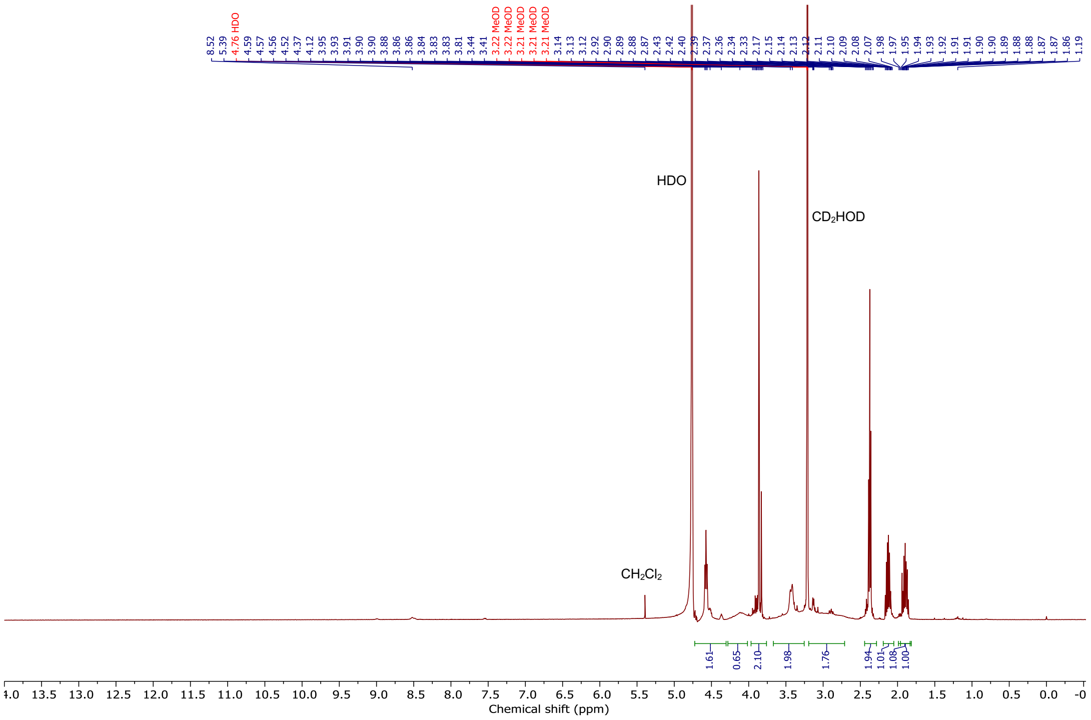

Fig. S65 ^{1}H NMR (400 MHz, CD_3OD) spectrum of **18** (Peak broadening due to the presence of conformers).

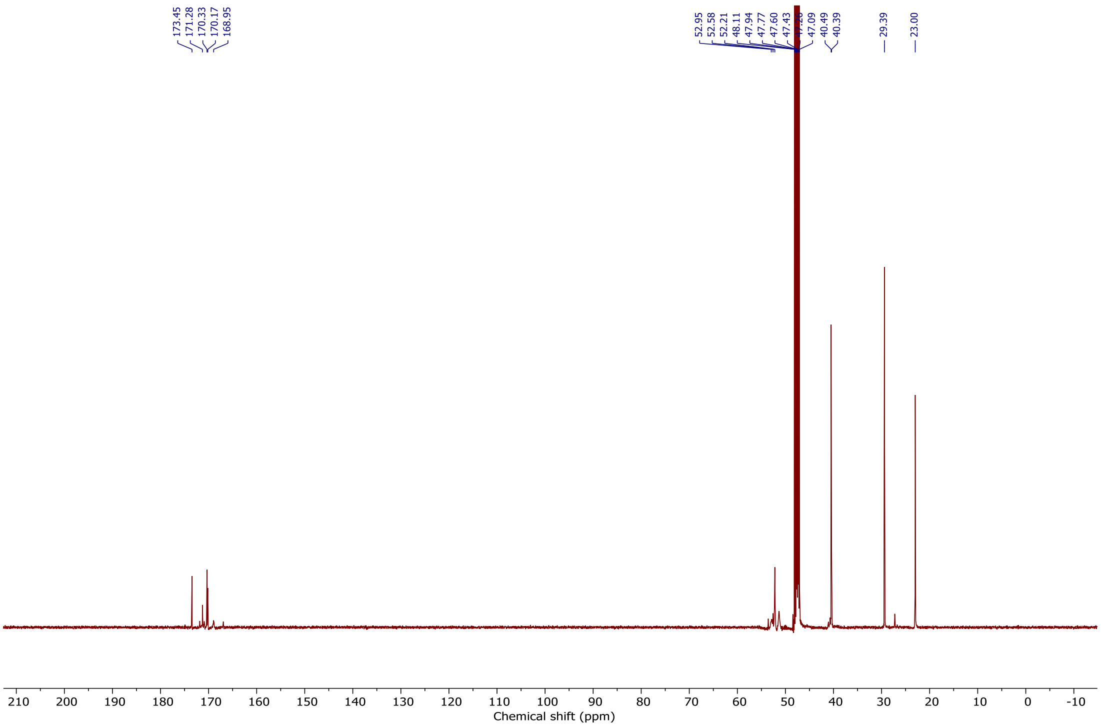

Fig. S66 ^{13}C NMR (125 MHz, CD_3OD) spectrum of **18**.

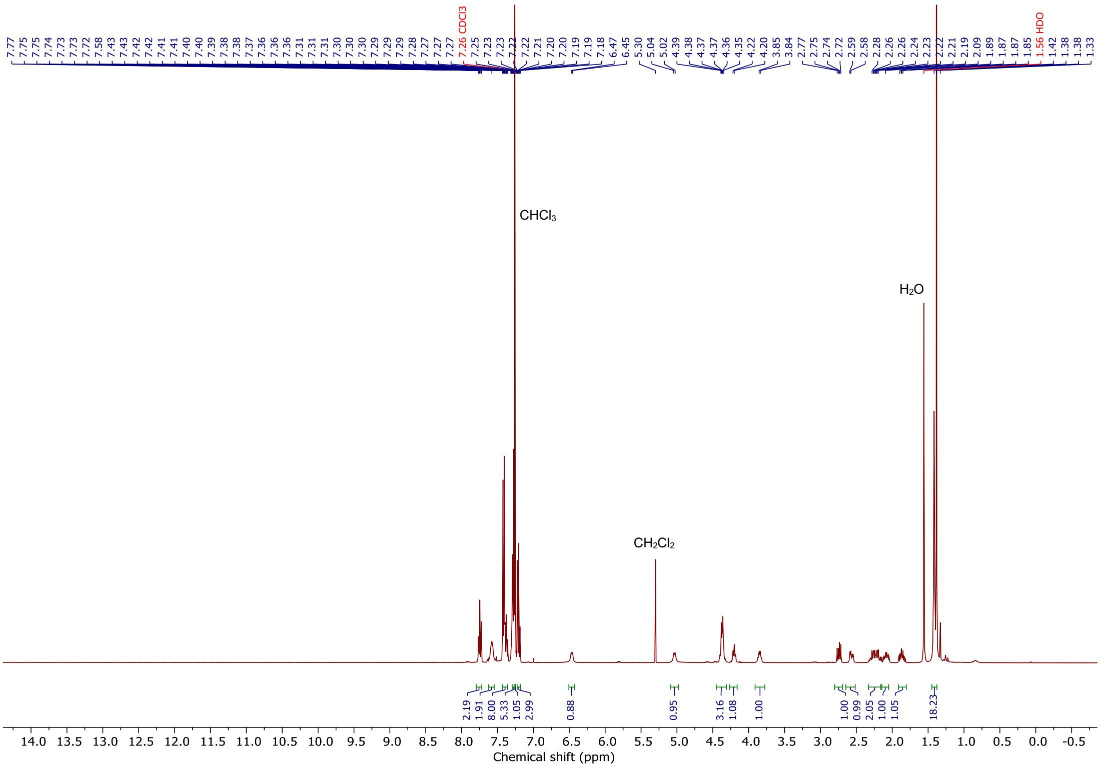

Fig. S67 ^{1}H NMR (400 MHz, $CDCl_3$) spectrum of **46**.

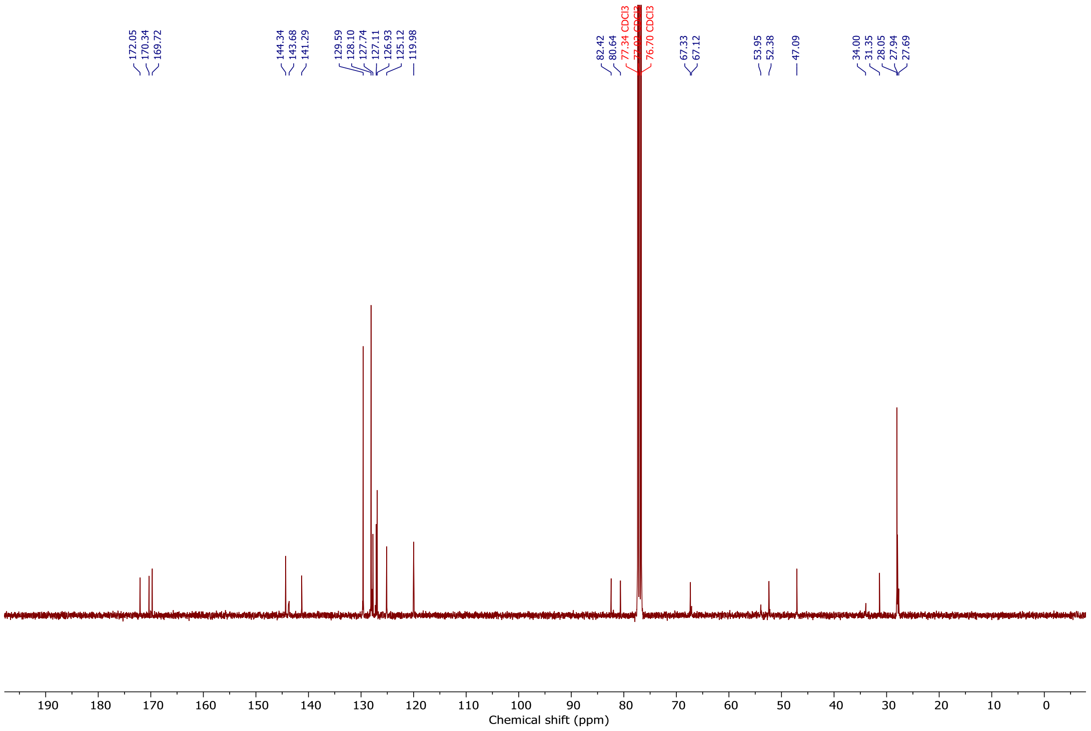

Fig. S68 ^{13}C NMR (125 MHz, $CDCl_3$) spectrum of **46**.

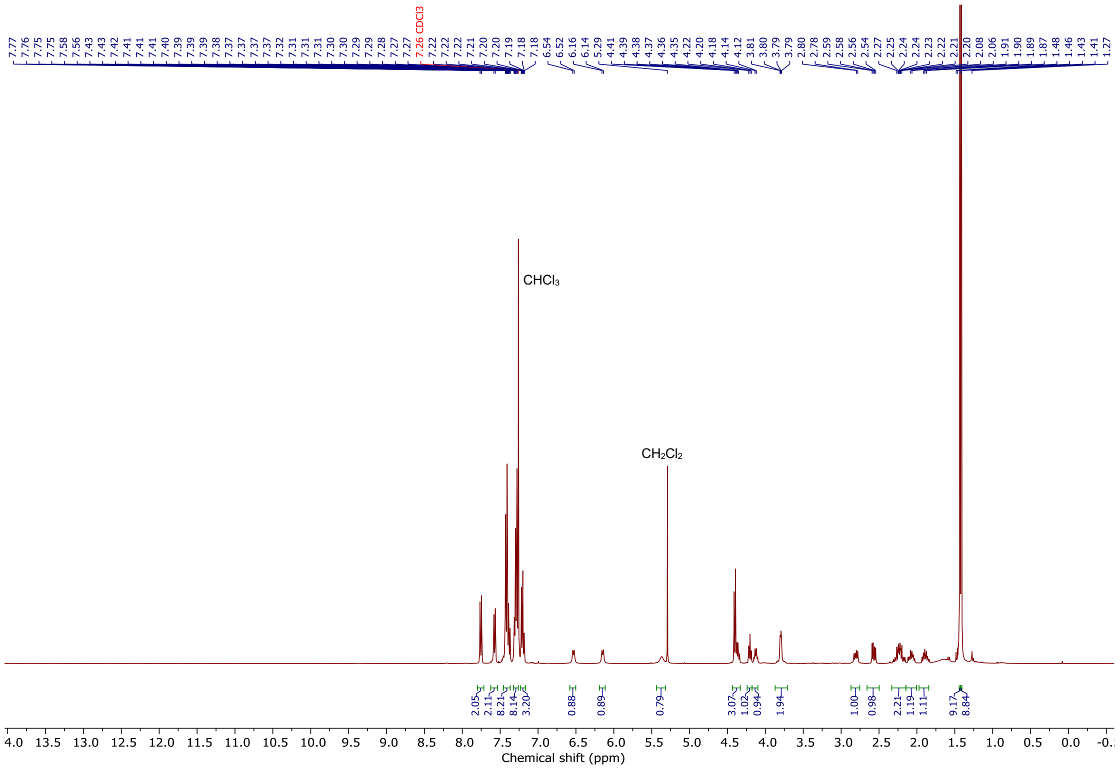

Fig. S69 ^{1}H NMR (400 MHz, $CDCl_3$) spectrum of **47**.

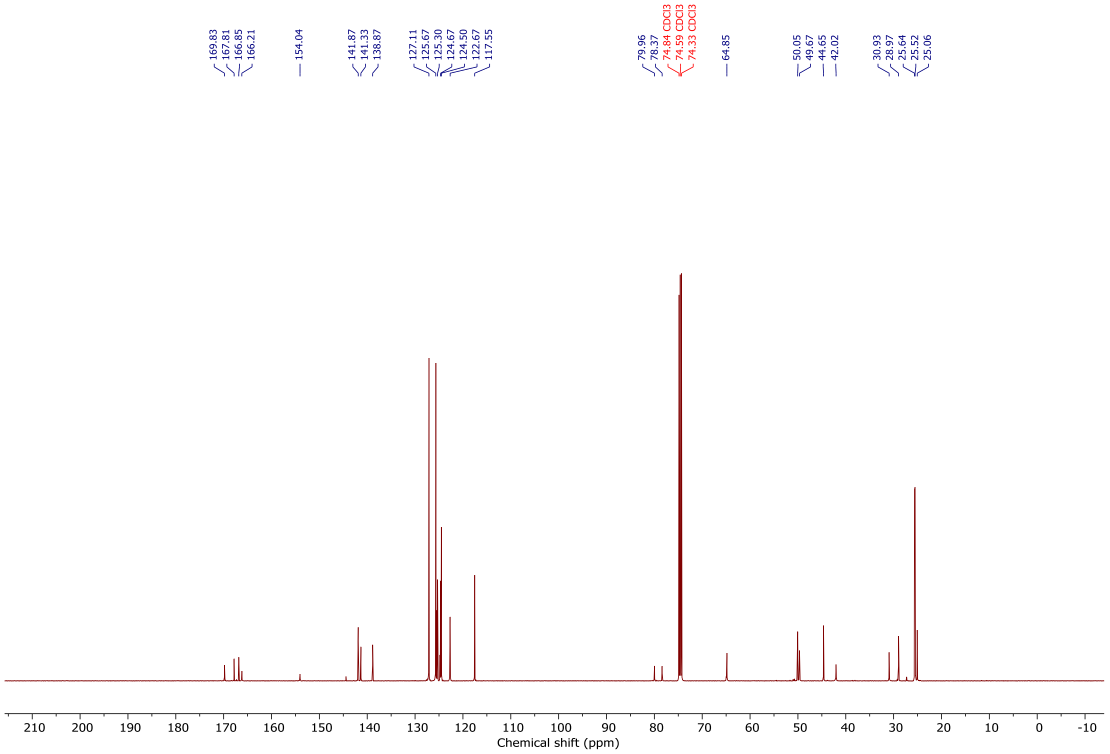

Fig. S70 ^{13}C NMR (125 MHz, $CDCl_3$) spectrum of **47**.

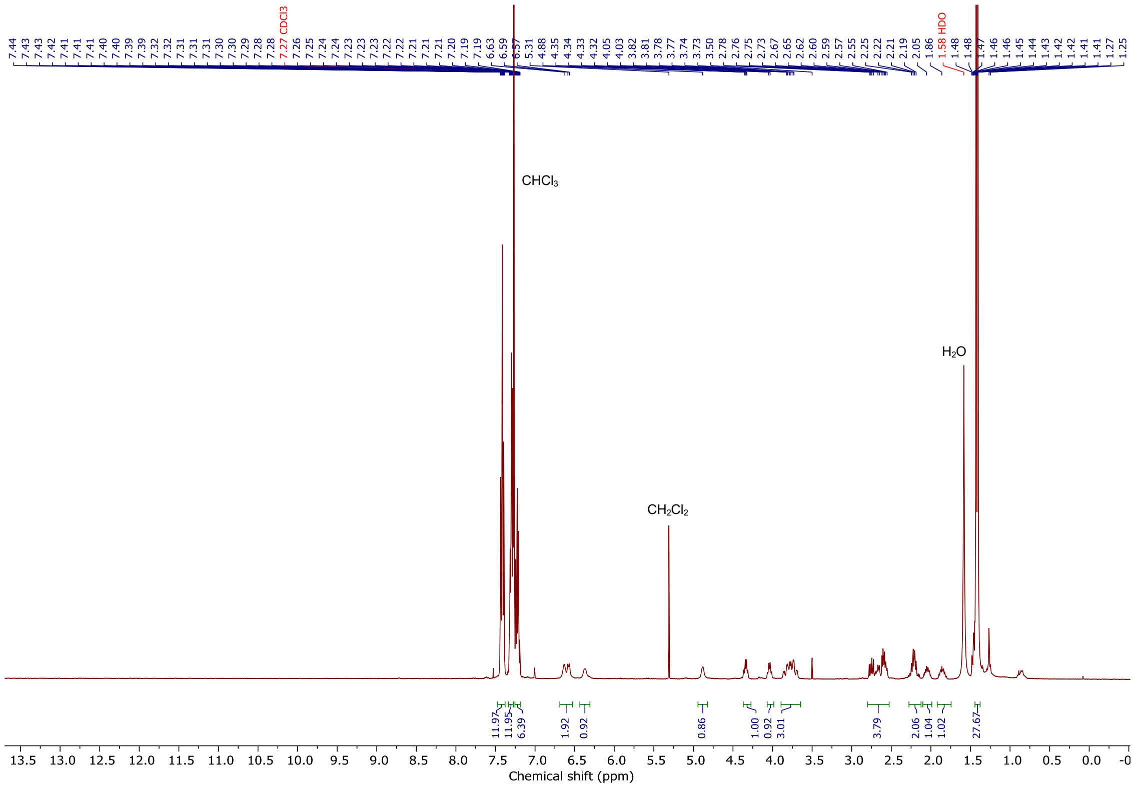

Fig. S71 ^{1}H NMR (400 MHz, $CDCl_3$) spectrum of **48**.

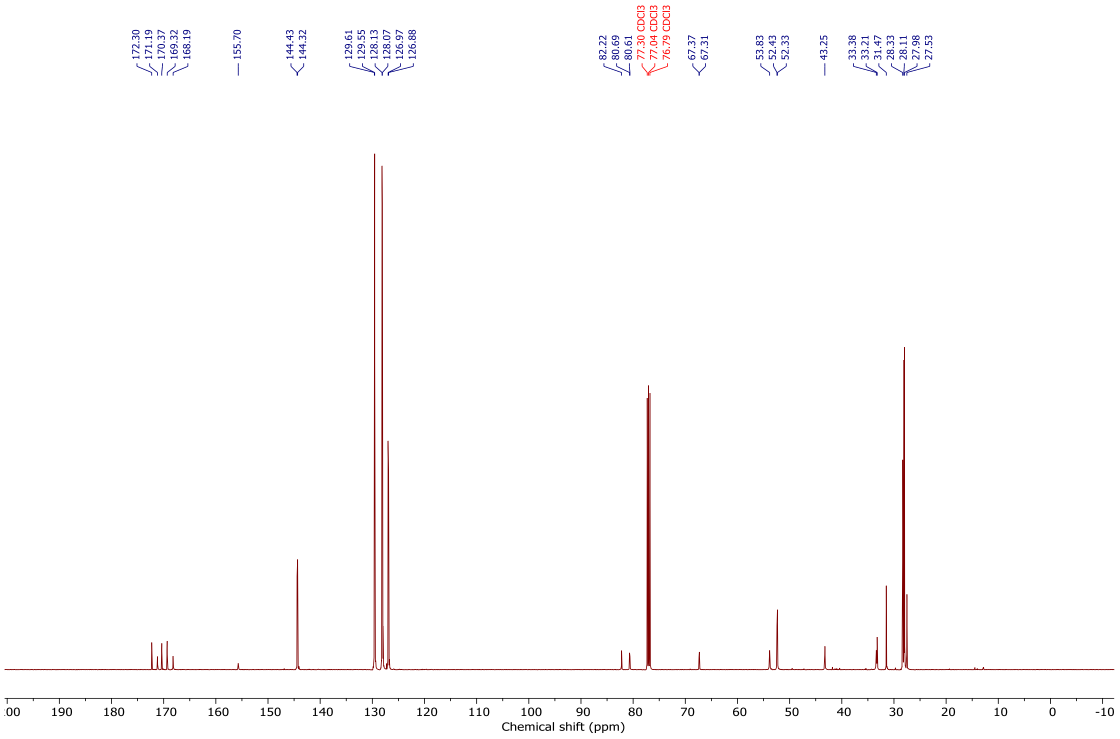

Fig. S72 ^{13}C NMR (125 MHz, $CDCl_3$) spectrum of **48**.

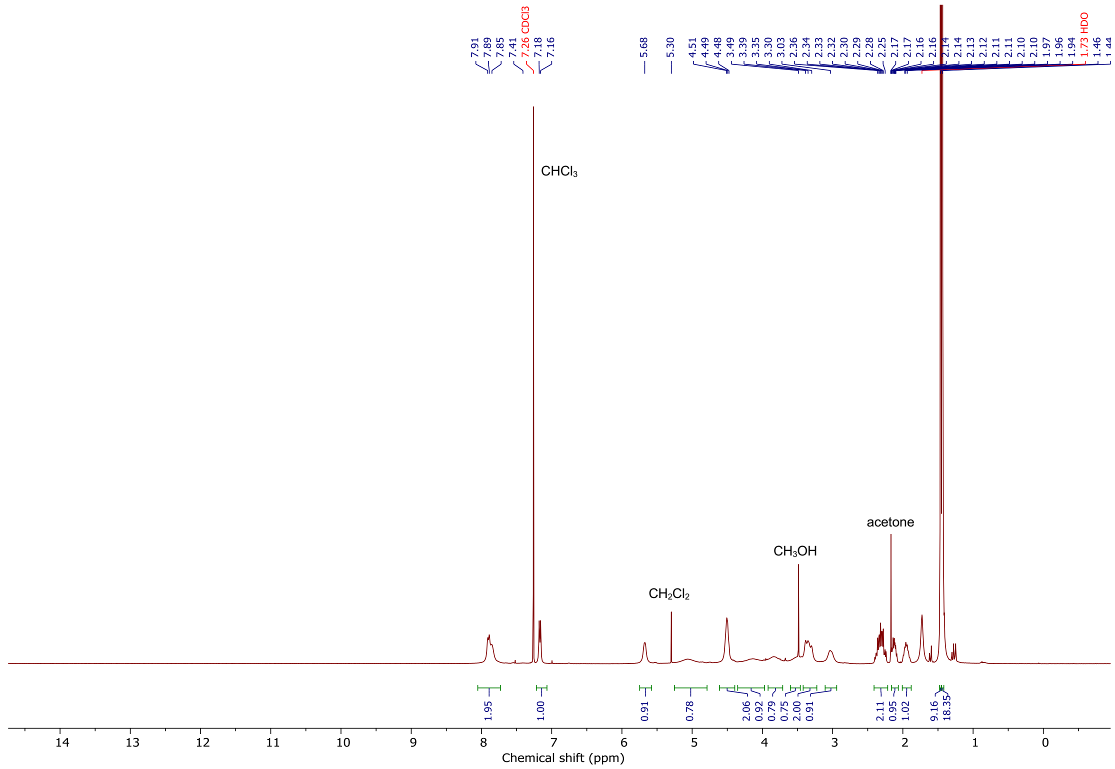

Fig. S73 ^{1}H NMR (400 MHz, $CDCl_3$) spectrum of **49**.

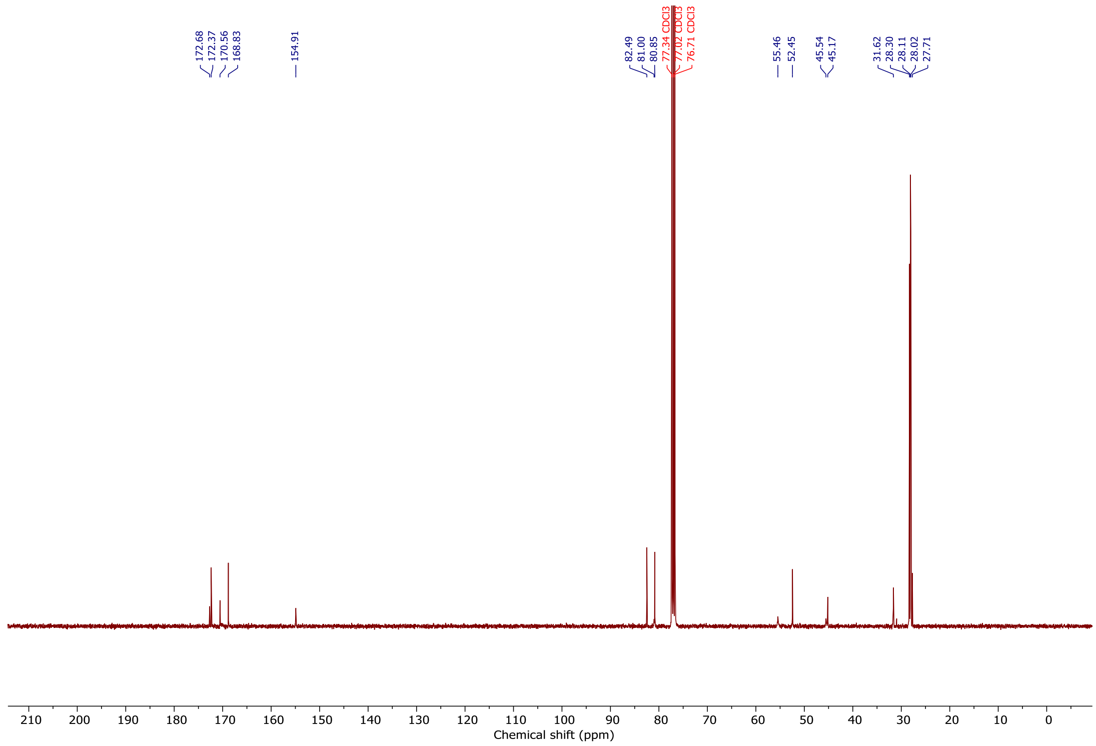

Fig. S74 ^{13}C NMR (125 MHz, $CDCl_3$) spectrum of **49**.

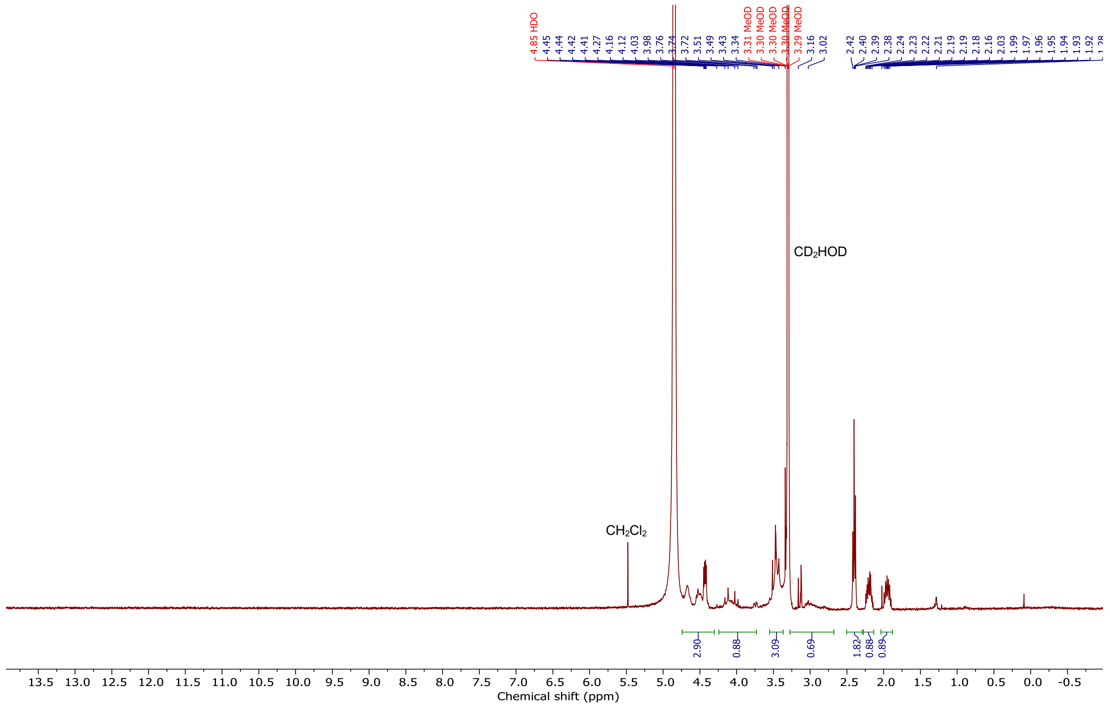

Fig. S75 ^{1}H NMR (400 MHz, CD_3OD) spectrum of **19** (Peak broadening due to the presence of conformers).

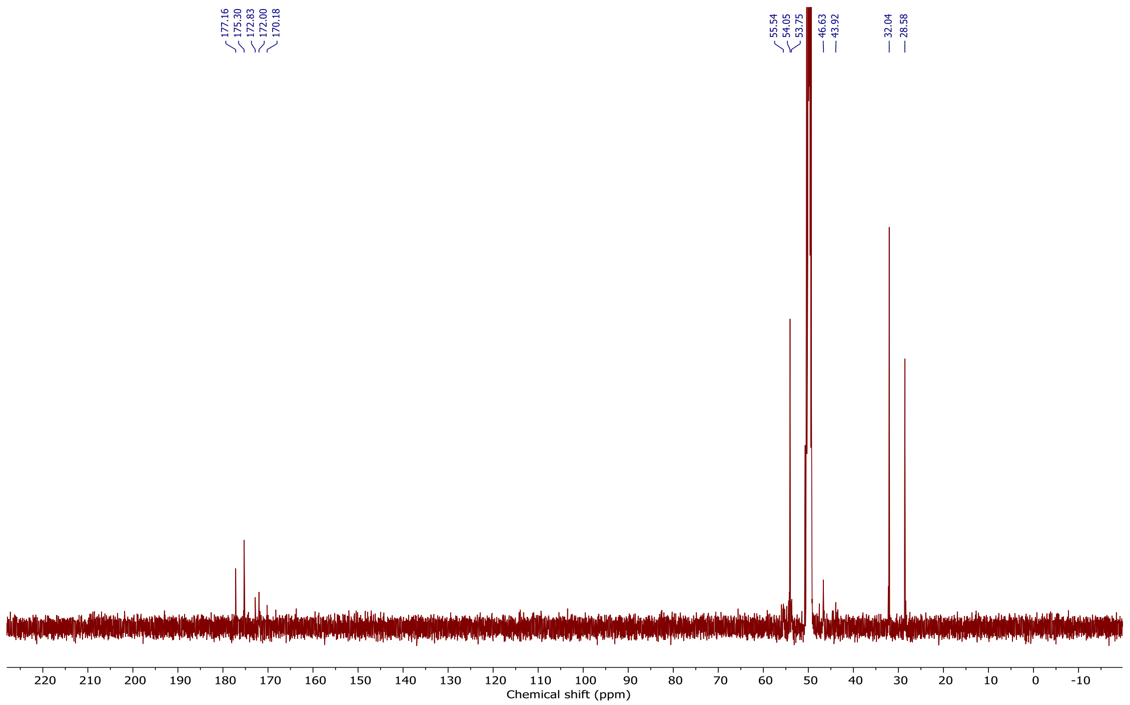

Fig. S76 ^{13}C NMR (125 MHz, CD_3OD) spectrum of **19**.

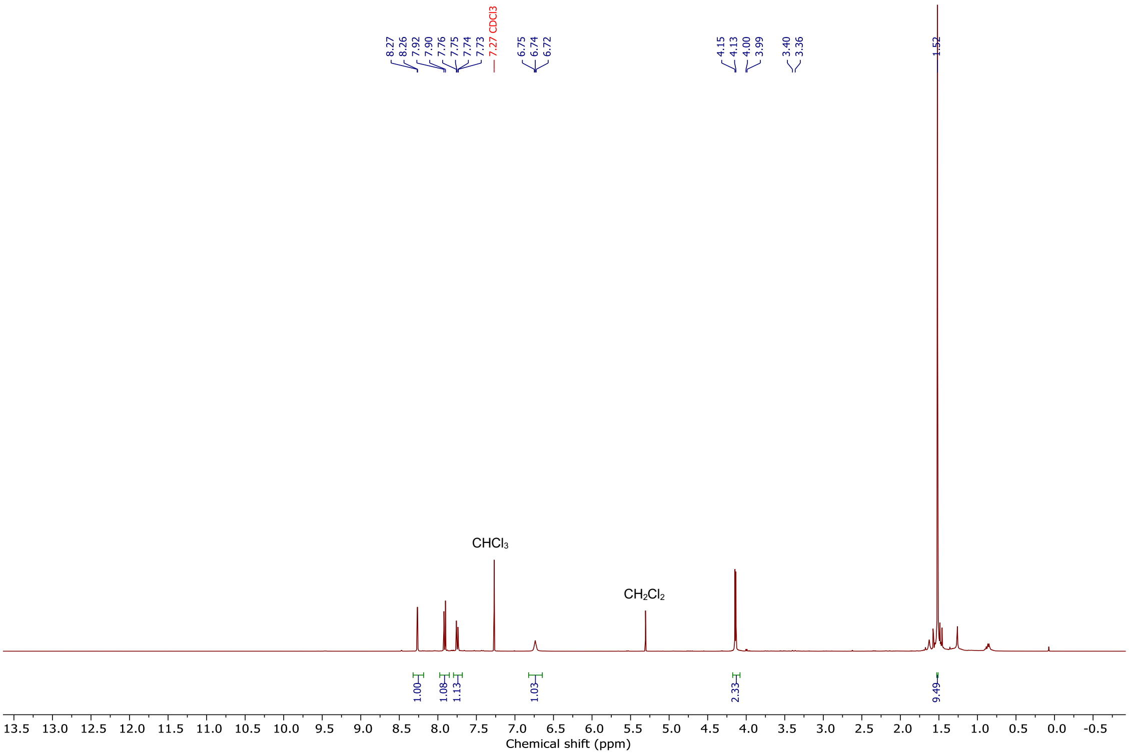

Fig. S77 ^{1}H NMR (400 MHz, $CDCl_3$) spectrum of **51**.

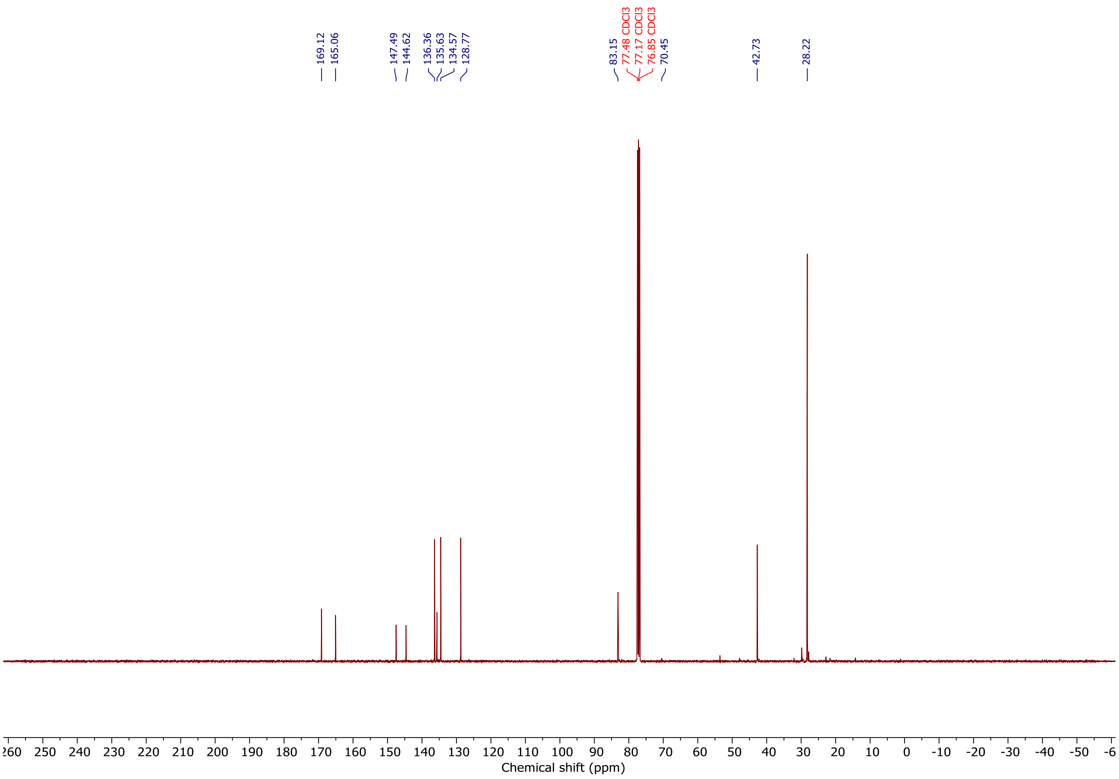

Fig. S78 ^{13}C NMR (100 MHz, $CDCl_3$) spectrum of **51**.

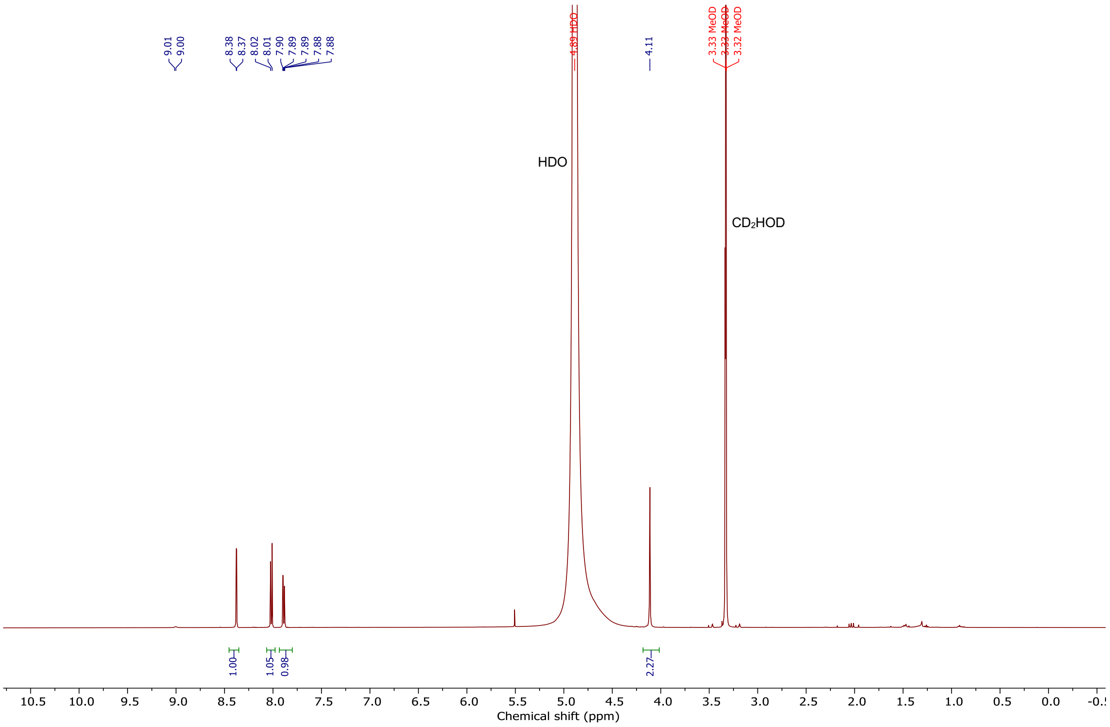

Fig. S79 ^{1}H NMR (400 MHz, CD_3OD) spectrum of **6**.

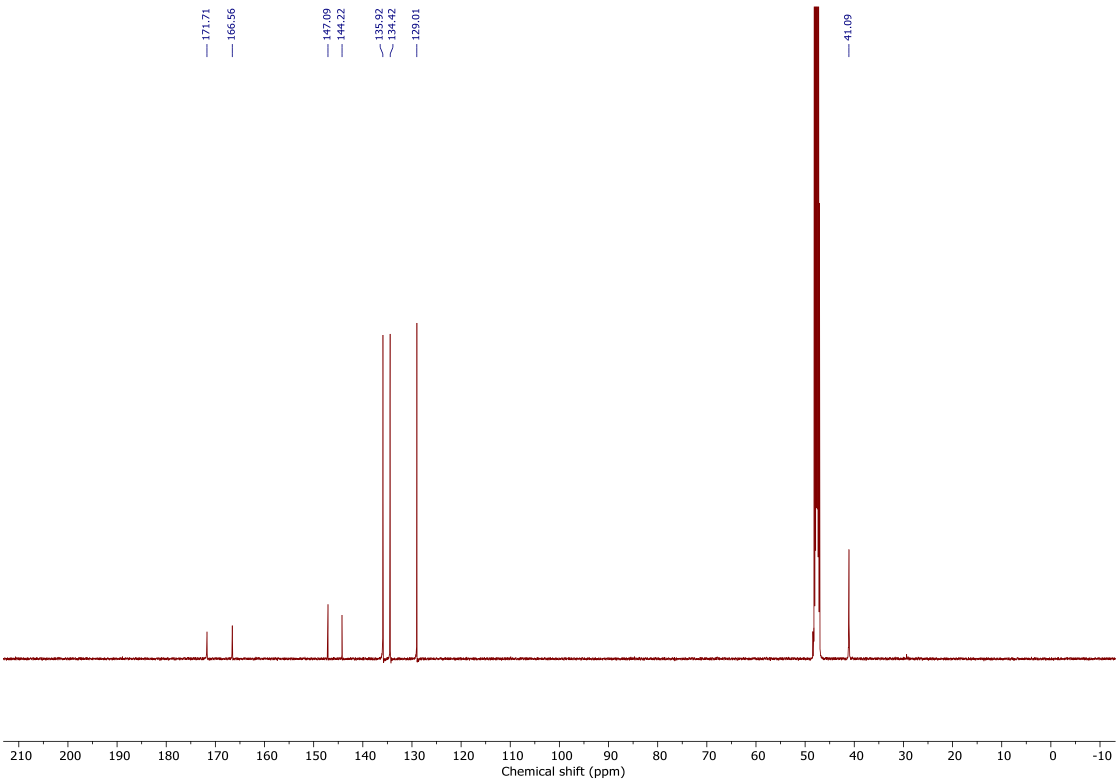

Fig. S80 ^{13}C NMR (125 MHz, CD_3OD) spectrum of **6**.

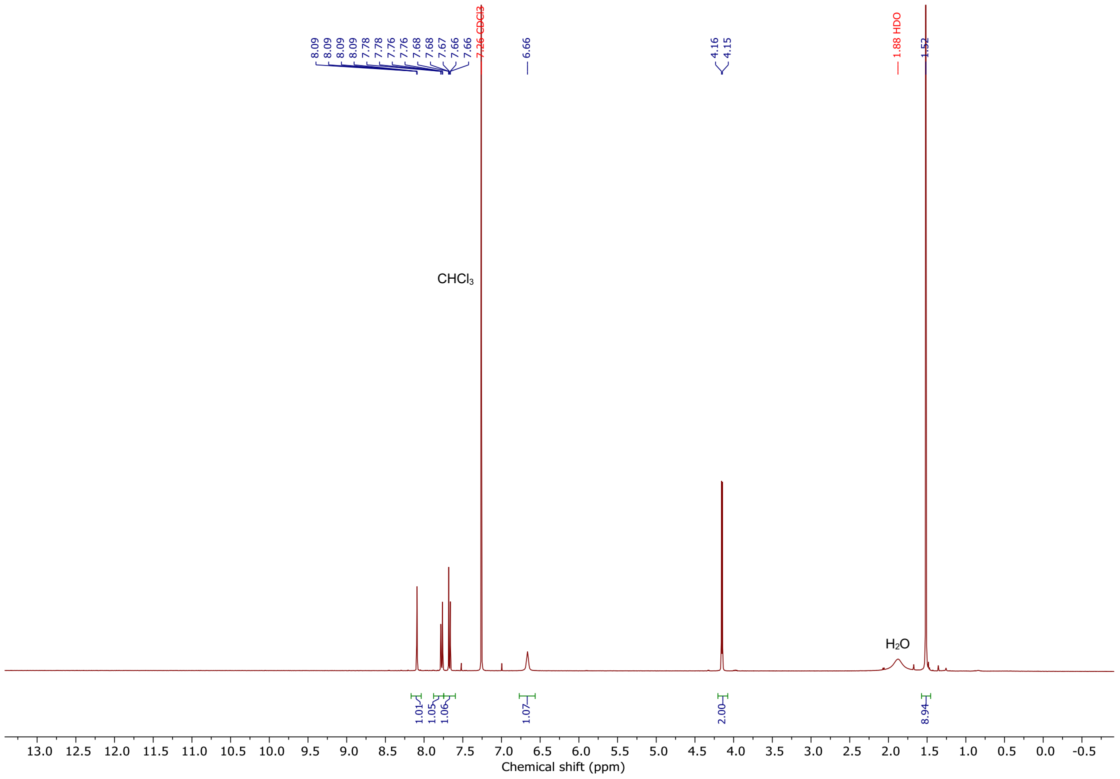

Fig. S81 ^{1}H NMR (400 MHz, $CDCl_3$) spectrum of **54**.

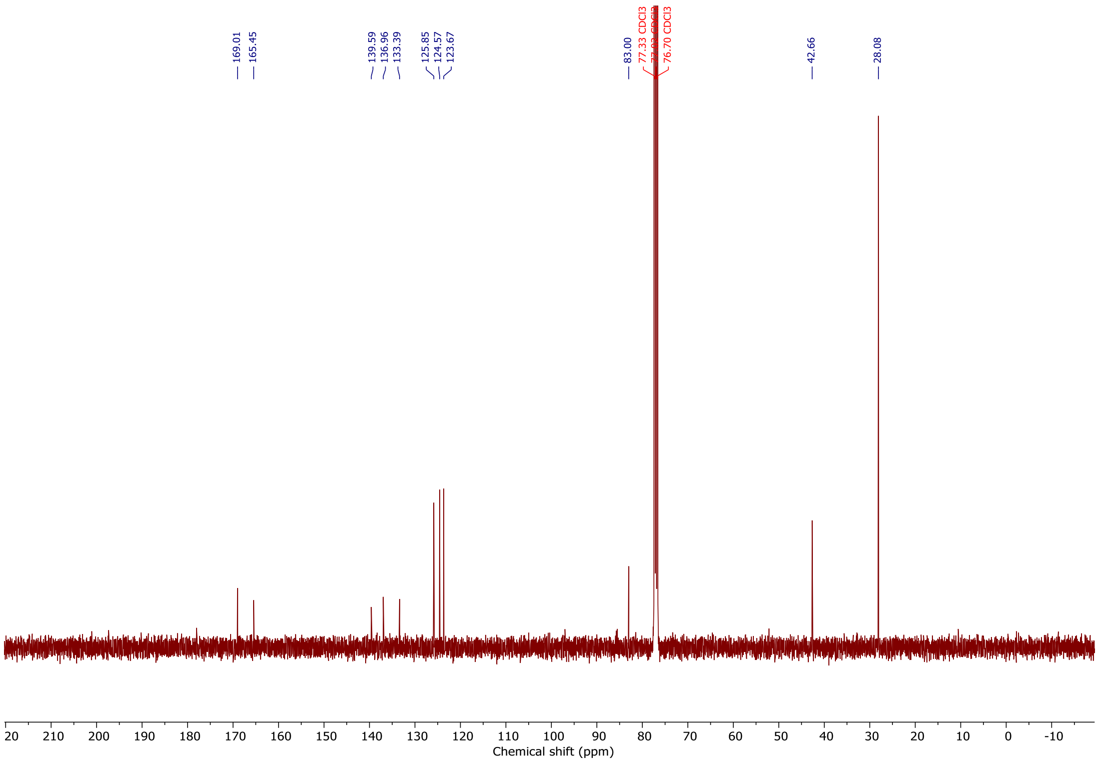

Fig. S82 ^{13}C NMR (125 MHz, $CDCl_3$) spectrum of **54**.

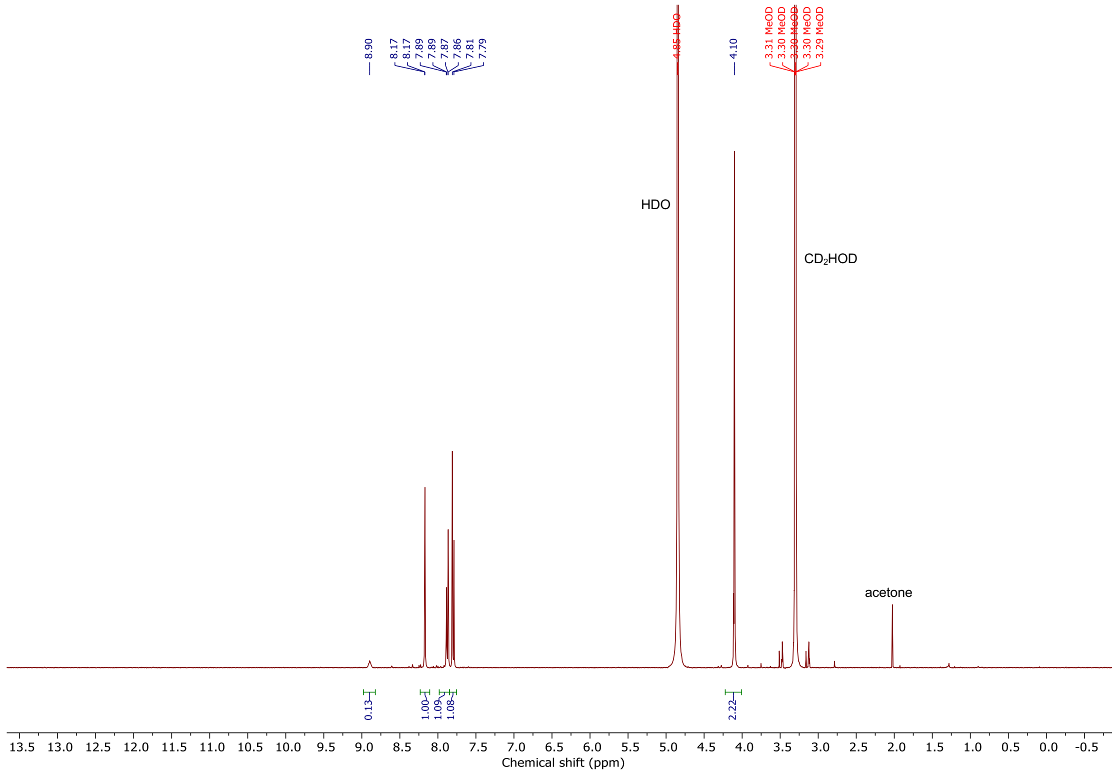

Fig. S83 ^{1}H NMR (400 MHz, CD_3OD) spectrum of **24**.

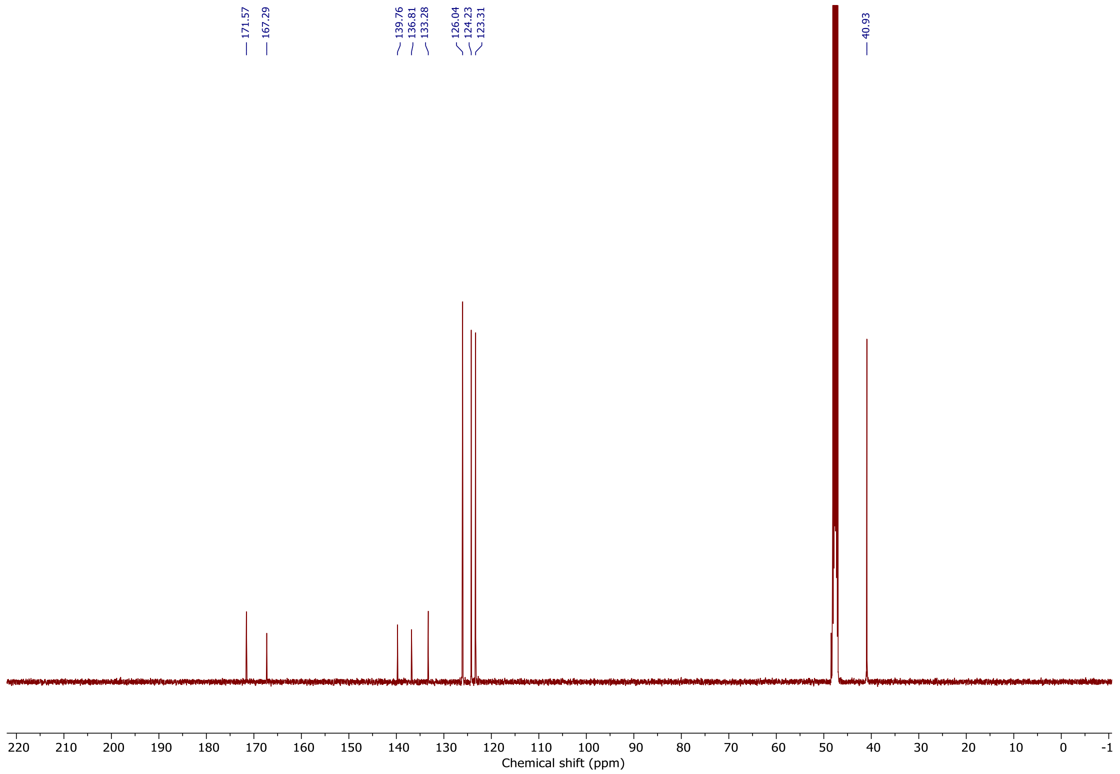

Fig. S84 ^{13}C NMR (125 MHz, CD_3OD) spectrum of **24**.

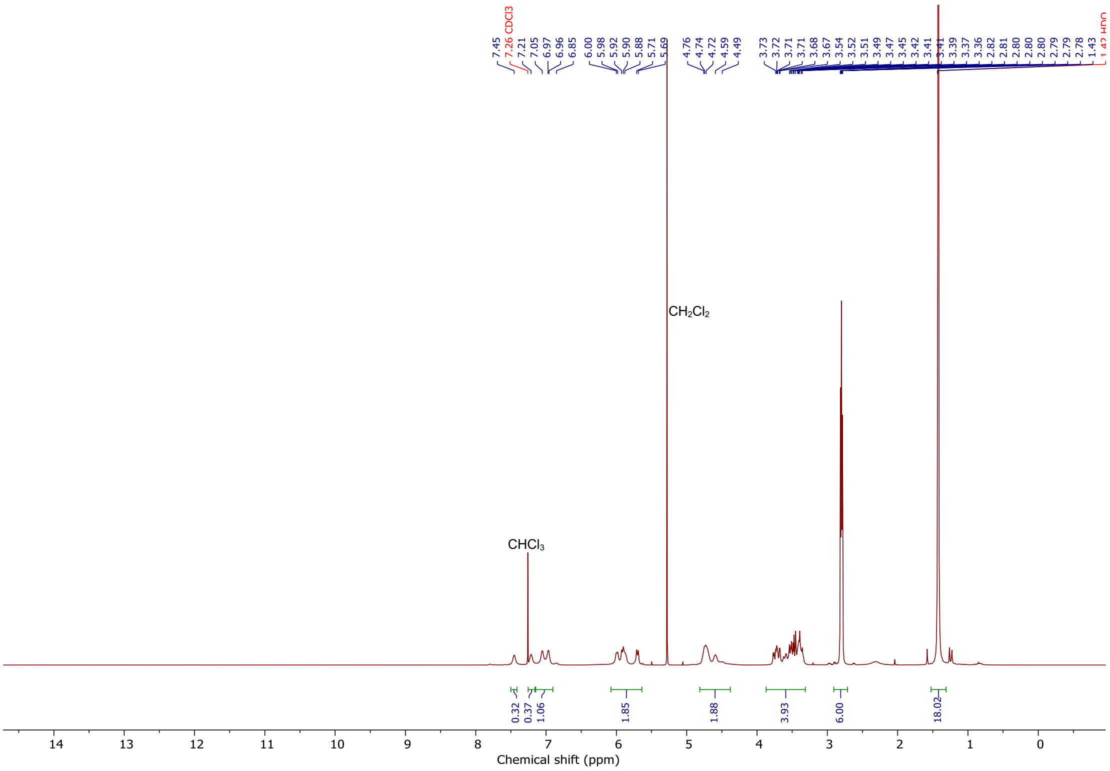

Fig. S85 ^{1}H NMR (400 MHz, $CDCl_3$) spectrum of **26**.

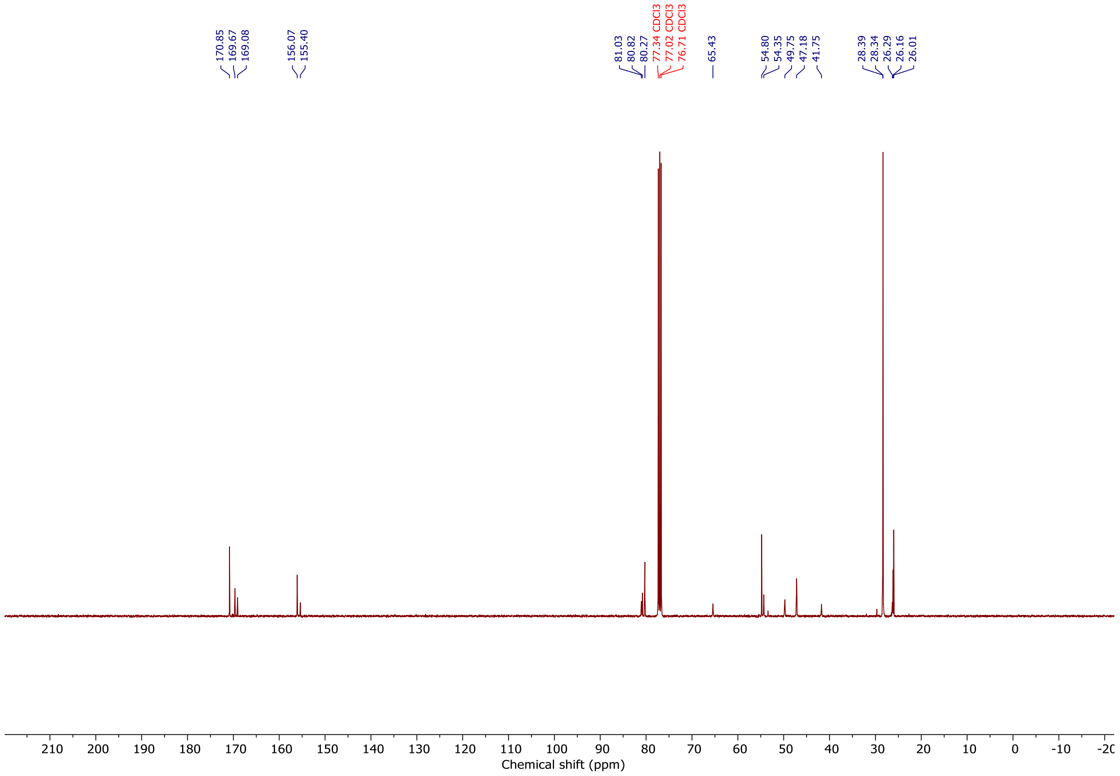

Fig. S86 ^{13}C NMR (125 MHz, $CDCl_3$) spectrum of **26**.

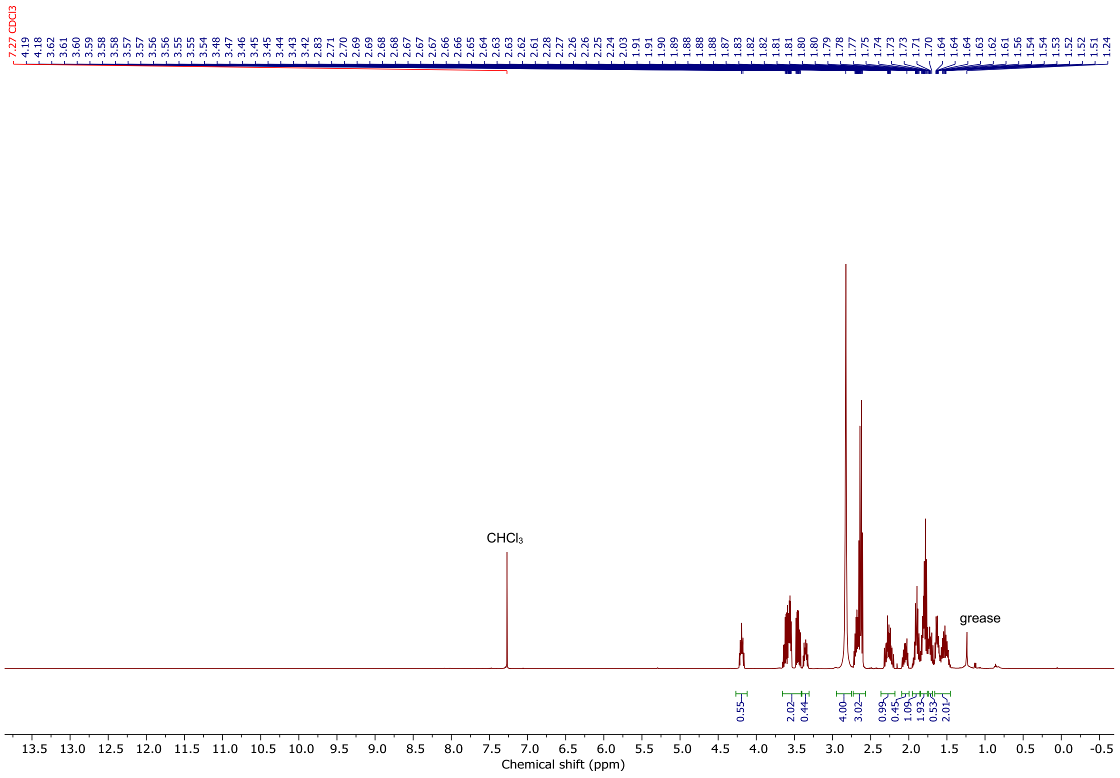

Fig. S87 ^{1}H NMR (400 MHz, $CDCl_3$) spectrum of **61** (mixture of 2 isomers).

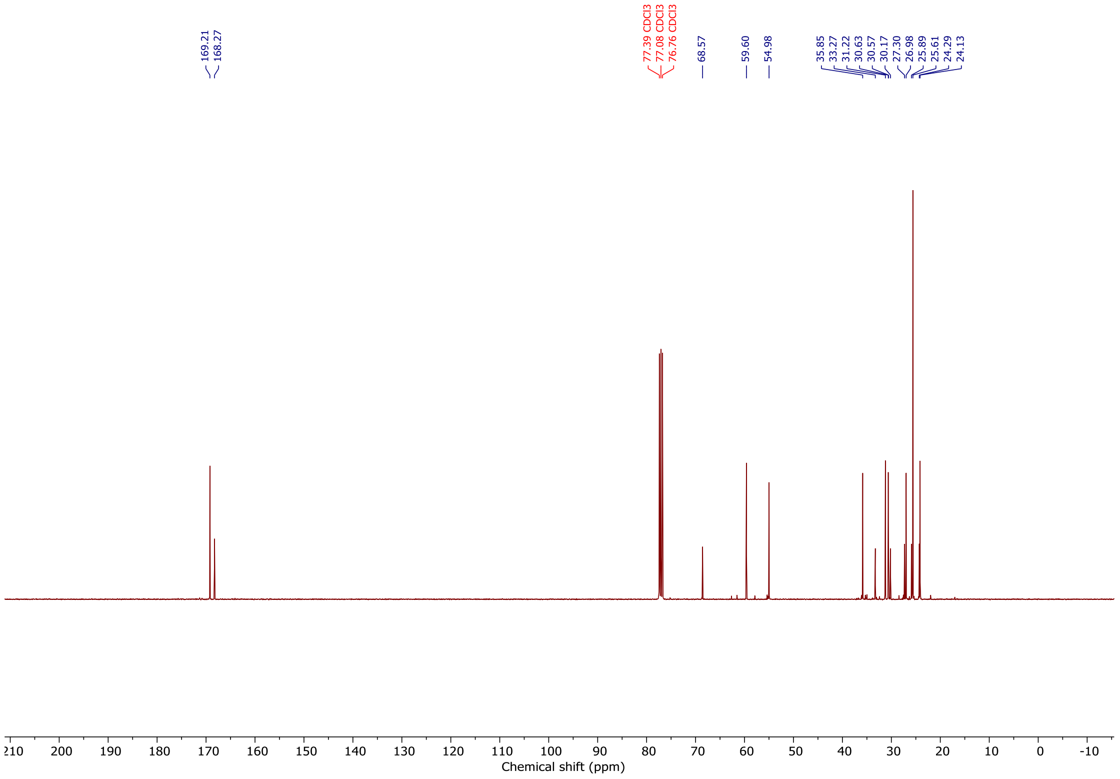

Fig. S88 ^{13}C NMR (100 MHz, $CDCl_3$) spectrum of **61**.

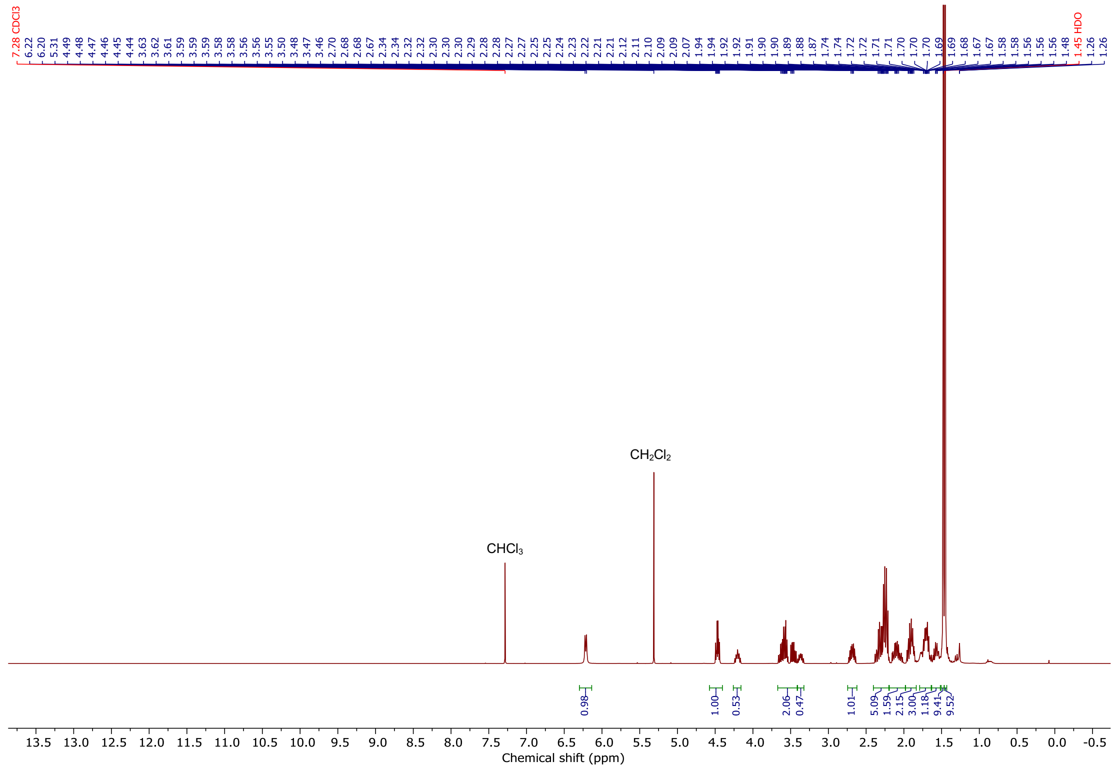

Fig. S89 ^{1}H NMR (400 MHz, $CDCl_3$) spectrum of **62** (mixture of 2 isomers).

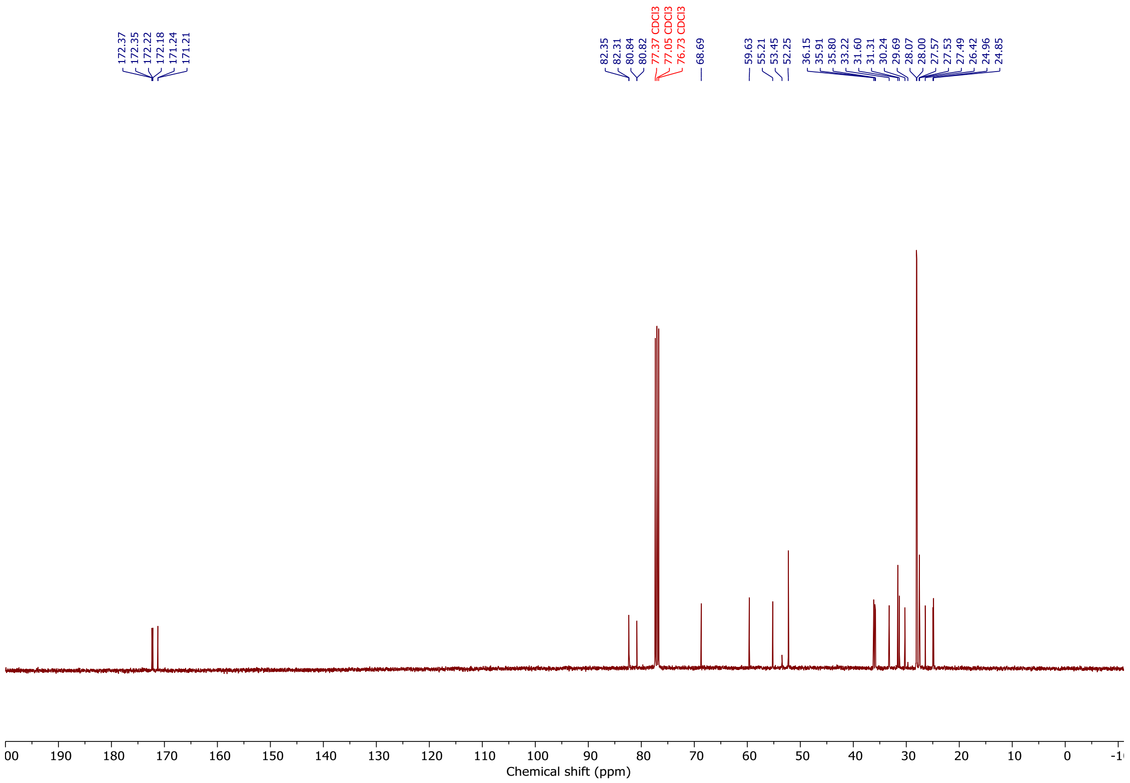

Fig. S90 ^{13}C NMR (100 MHz, $CDCl_3$) spectrum of **62**.

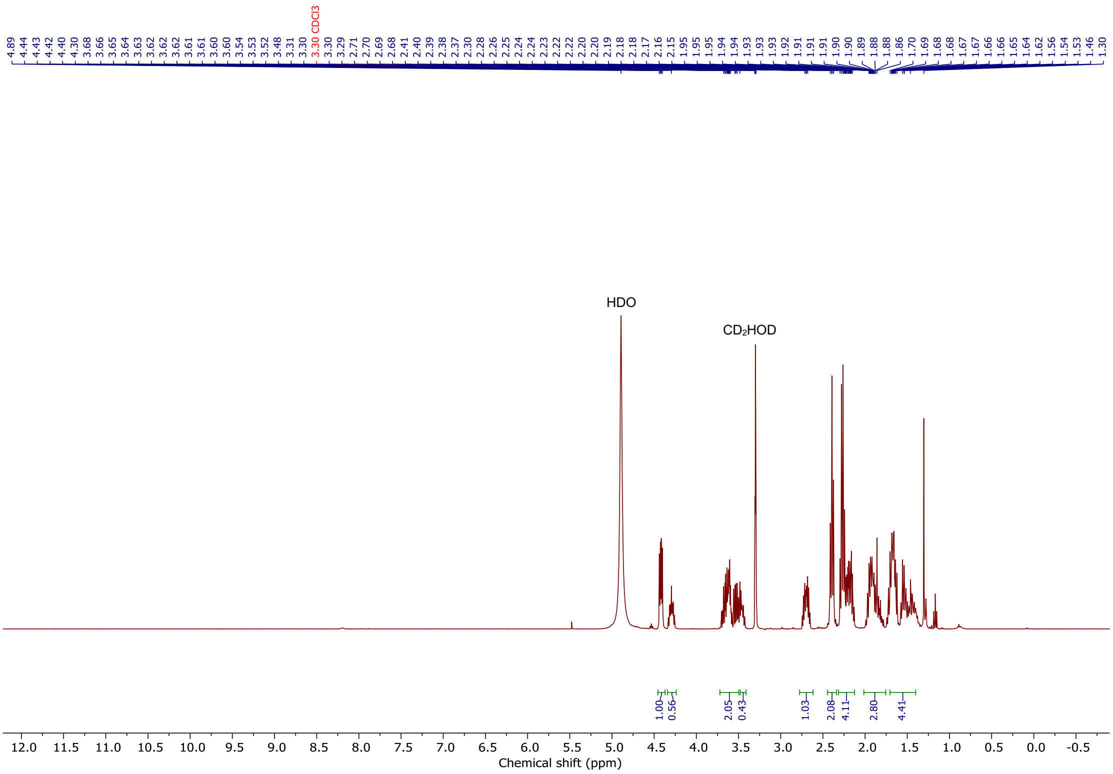

Fig. S91 ^{1}H NMR (400 MHz, CD_3OD) spectrum of **15** (mixture of 2 isomers).

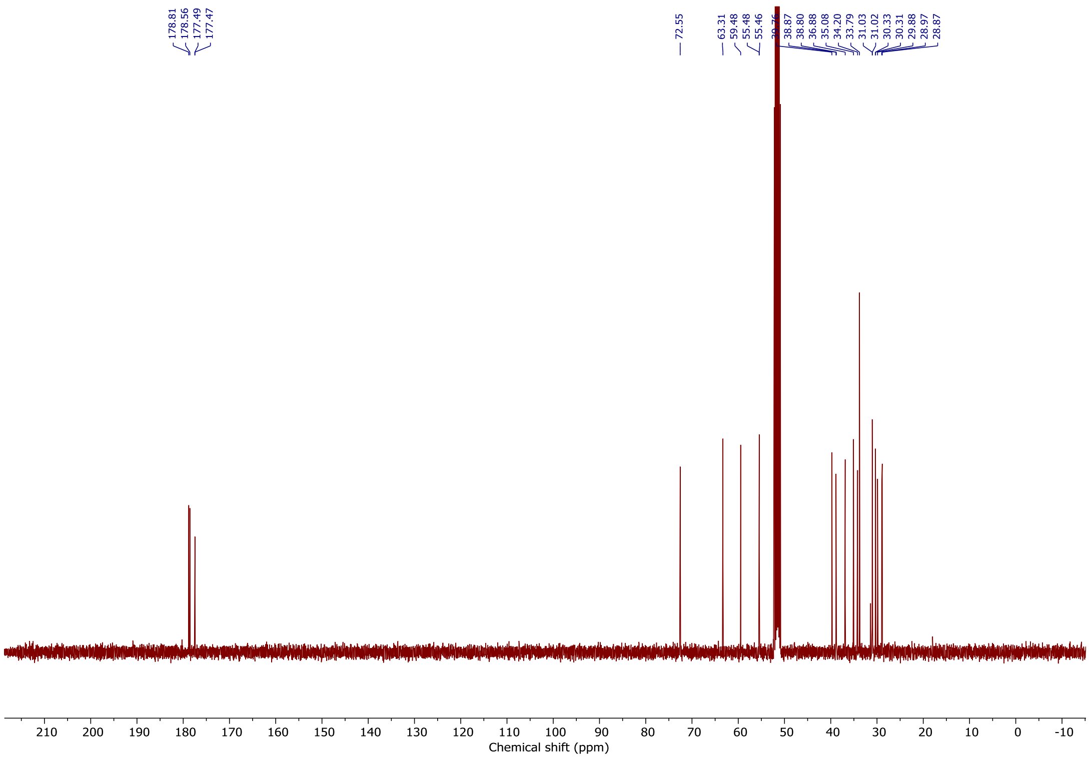

Fig. S92 ^{13}C NMR (100 MHz, $CDCl_3$) spectrum of **15**.

8. HPLC

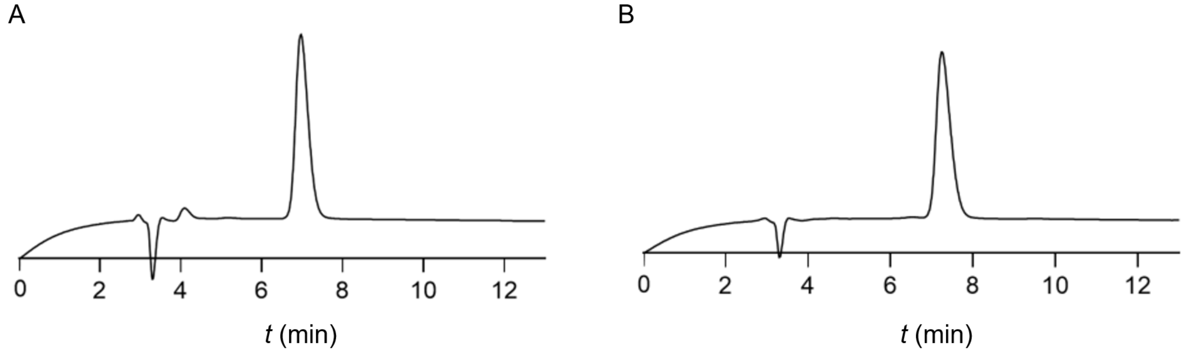

Fig. S93 HPLC of A) **18** (R_t = 6.97 min) and B) **19** (R_t = 7.24 min).
